# Single-frame deep-learning super-resolution microscopy for intracellular dynamics imaging

**DOI:** 10.1038/s41467-023-38452-2

**Published:** 2023-05-18

**Authors:** Rong Chen, Xiao Tang, Yuxuan Zhao, Zeyu Shen, Meng Zhang, Yusheng Shen, Tiantian Li, Casper Ho Yin Chung, Lijuan Zhang, Ji Wang, Binbin Cui, Peng Fei, Yusong Guo, Shengwang Du, Shuhuai Yao

**Affiliations:** 1 Department of Chemical and Biological Engineering, The Hong Kong University of Science and Technology, Hong Kong, China; 2 Division of Life Science, The Hong Kong University of Science and Technology, Hong Kong, China; 3 School of Optical and Electronic Information, Huazhong University of Science and Technology, 430074 Wuhan, China; 4 Department of Mechanical and Aerospace Engineering, The Hong Kong University of Science and Technology, Hong Kong, China; 5 School of Pharmaceutical Sciences, Guizhou University, 550025 Guizhou, China; 6 Department of Physics, The Hong Kong University of Science and Technology, Hong Kong, China; 7 Department of Physics, The University of Texas at Dallas, Richardson, TX 75080 USA

**Keywords:** Super-resolution microscopy, Fluorescence imaging

## Abstract

Single-molecule localization microscopy (SMLM) can be used to resolve subcellular structures and achieve a tenfold improvement in spatial resolution compared to that obtained by conventional fluorescence microscopy. However, the separation of single-molecule fluorescence events that requires thousands of frames dramatically increases the image acquisition time and phototoxicity, impeding the observation of instantaneous intracellular dynamics. Here we develop a deep-learning based single-frame super-resolution microscopy (SFSRM) method which utilizes a subpixel edge map and a multicomponent optimization strategy to guide the neural network to reconstruct a super-resolution image from a single frame of a diffraction-limited image. Under a tolerable signal density and an affordable signal-to-noise ratio, SFSRM enables high-fidelity live-cell imaging with spatiotemporal resolutions of 30 nm and 10 ms, allowing for prolonged monitoring of subcellular dynamics such as interplays between mitochondria and endoplasmic reticulum, the vesicle transport along microtubules, and the endosome fusion and fission. Moreover, its adaptability to different microscopes and spectra makes it a useful tool for various imaging systems.

## Abstract (web summary)

Multi-frame super-resolution microscopy is hampered by long acquisition times and phototoxicity, which hinder its use for live-cell imaging. Here, authors propose a deep-learning-based single-frame super-resolution approach to image cellular dynamics with high spatiotemporal resolution.

## Introduction

Live-cell fluorescence imaging, requiring both low phototoxic illumination and a high imaging speed, is usually performed with a wide-field (WF) fluorescence microscope^1^. The spatial resolution of a conventional fluorescence microscope is limited by diffraction and thus unable to resolve subcellular structures smaller than 200 nm. In the past two decades, various types of super-resolution microscopy surpassing the diffraction limit have been developed. For example, structured illumination microscopy (SIM)^2^ can be used for live-cell imaging with low invasiveness; however, it only improves the spatial resolution of images by a factor of up to 2. Although advanced SIM^3^ has improved the resolution to ~60 nm, multiple frames are still required to construct a single super-resolution (SR) image. Stimulated emission depletion (STED) microscopy^4^ can achieve an ~50 nm resolution using highly intense light pulses, but point-to-point scanning makes STED too slow for live-cell imaging. Single-molecule localization microscopy (SMLM)^5–8^, including photoactivated localization microscopy (PALM)^6,7^ and stochastic optical reconstruction microscopy (STORM)^5^, further enhances the spatial resolution by a factor of 10 (~20 nm) but typically requires more than thousands of frames with separated single-molecule fluorescence events to reconstruct one SR image; hence, in rare cases, SMLM has been applied to live cells at a second-scale temporal resolution^9–11^. To perform time-resolved and noninvasive super-resolution imaging, numerous advanced labeling strategies^12–14^, optical imaging systems^15,16^, and image reconstruction methods^17–19^ have been explored in recent decades. Nonetheless, inherent tradeoffs among spatial and temporal resolutions, the achievable signal intensity and cytotoxicity must be made due to the physical boundaries of optical systems^20^.

The rapid development of artificial intelligence has led to many traditional hardware limits being surpassed. Various deep learning networks have displayed excellent performance in the single-image super-resolution (SISR) task^21–23^ which usually transforms a single low-resolution (LR) photograph to a high-resolution (HR) photograph. The focus of the SISR task for realistic photographs is to enhance texture and improve visual quality^24,25^. In contrast, super-resolution tasks for microscopic images demand ultrastructure recovery from diffraction-limited images with high accuracy. Recently, popular neural networks in computer vision have been modified to enhance the resolution of microscopic images, for instance, from low magnification to high magnification^26,27^, confocal to STED^27,28^, and total internal reflection fluorescence (TIRF) or WF to SIM^27–29^; and also combined with PALM and STORM to accelerate the localization process of SMLM reconstruction^30^ and reduce the number of frames of single-molecule images required for SMLM reconstruction^31,32^. However, due to the large resolution gap between the LR images acquired by WF microscopes and HR images obtained from SMLM reconstructions, multiple frames of LR images with single-molecule fluorescence events are still required to reconstruct an SR image. Therefore, the fundamental problems of multi-frame super-resolution imaging, such as the long acquisition time and photobleaching-induced phototoxicity in localization microscopy, still hinder its application in the imaging of live-cell dynamics.

In this work, we first explore the possibility of using a neural network to directly transform a single diffraction-limited image to an SR image with a 10-fold higher resolution. By applying an enhanced super-resolution generative adversarial network (ESRGAN)^25^, multi-component loss function, and prior information regulation, we develop a super-resolution network (SRN) that can resolve a single diffraction-limited frame to an SR image with up to a 10-fold resolution improvement. Then, we investigate the challenges of implementing this SRN for real-time live-cell observations where the acquired images normally have an ultralow signal-to-noise ratio (SNR). By deploying a signal-enhancement network (SEN) in advance to progressively optimize the image SNR and resolution, we are able to reduce the requirement on the input SNR for satisfactory reconstruction quality, thus allowing for high-speed live-cell imaging without sacrificing the spatial resolution. Taken together, we propose a single-frame super-resolution microscopy (SFSRM) approach that allows us to reveal time-resolved intracellular events in live cells, for instance, the vesicle transport dynamics, the endosome fusion and fission process, and mitochondria-endoplasmic reticulum interactions. Moreover, we demonstrate that the well-trained SFSRM networks can be used in various imaging systems without further training, making super-resolution imaging possible for laboratories lacking training datasets.

## Results

### SFSRM based on joint-optimization-enhanced deep learning networks

The central goal of the deep-learning-based microscopic image SR tasks is to reconstruct the high-frequency structures with high accuracy from LR images. Therefore, in pursuit of high fidelity, the mostly used loss functions in microscopic image restoration are mean absolute error (MAE) loss, mean square error (MSE) loss, and structural similarity (SSIM) loss. These loss functions which focus on pixel-wise differences between the network output and the GT image can achieve a high peak signal-to-noise ratio and SSIM index, but suffer from oversmoothed reconstruction result and loss of high-frequency details^24^ (Supplementary Fig. 1). By contrast, perceptual loss and adversarial loss^25^, which have been extensively utilized in photograph SR tasks to restore the high-frequency details, are regarded inappropriate for microscopic image restoration because undesirable artifacts can be induced^24^.

**Fig. 1:**
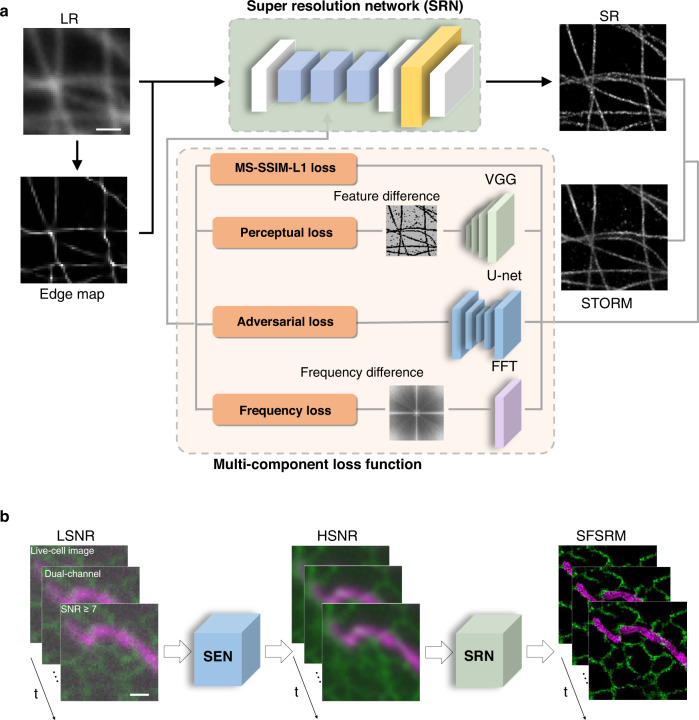
The architecture of SFSRM. **a** The super-resolution network (SRN) architecture. The SRN is trained with simulated low-resolution (LR) and ground-truth (GT) image pairs or experimental wide-field (WF) and STORM image pairs obtained from the STORM microscope. The LR/WF image is preprocessed by a sub-pixel edge-detector to extract the edge map, and both of them are then fed as inputs to the network. A multi-component loss function is adopted to train the network. The multi-component loss function includes the following parts: (i) MS-SSIM-L1 loss which measures the pixel-wise difference between the SR and GT/STORM images by multi-scale similarity and mean absolute error; (ii) perceptual loss which measures the difference between feature maps of SR and GT/STORM images extracted by the VGG network; (iii) adversarial loss returned by the U-net discriminator which distinguishes the GT/STORM image from the SR image; (iv) frequency loss which compares the frequency spectrum difference of the SR and GT/STORM image in a specified frequency region. **b** Workflow of SFSRM. To implement SFSRM in high frame-rate live-cell imaging. The low-SNR (LSNR) image sequence acquired from live cells first goes through the signal-enhancement network (SEN) to improve signal intensity (or SNR), and the intermediate result (HSNR image sequence) is input to the SRN for the reconstruction of the SR image sequence. Scale bar: 1 µm.

To achieve high-frequency detail reconstruction, here we investigated the possibility of using perceptual loss and adversarial loss for microscopic image restoration. We propose a multi-component loss function containing (i) the combination of multi-scale structure similarity loss and mean absolute error loss, noted as MS-SSIM-L1 loss, to improve the pixel-wise reconstruction accuracy, (ii) the perceptual loss to generate high-frequency structures, (iii) the adversarial loss from a U-net discriminator to provide pixel-wise feedback to the generator about whether the reconstructed image is true or fake, (iv) the frequency loss to suppress the high-frequency artifacts (Fig. 1a). To quantitatively assess the functionality of the proposed loss function, we simulated randomly distributed polymer lines in the GT images with a pixel size of 10 nm. The GT images were then blurred by a Gaussian kernel of 200-nm full-width-half-maximum (FWHM) size to generate the corresponding LR images. The result shows that the multi-component loss has notably improved the fine-structure reconstruction capability of the network compared to the conventional pixel-wise loss and effectively suppressed the artifacts in the original ESRGAN (Supplementary Fig. 2). We repeated the experiments on 30 images and constantly found that the network trained with the multi-component loss was able to restore fine structures from blurred LR images and achieved an MS-SSIM index of ~0.98 with respect to the GT, manifesting the capability of the network to transform a single diffraction-limited image to an SR image with a 10-fold resolution increase under the noise-free condition.

Unfortunately, the reconstruction quality quickly degrades if the image is corrupted by noise (Supplementary Fig. 3, without the edge map), which is reasonable since single-image super-resolution restoration is already an ill-posed problem, and noise will add further complexity to this task. Determining how to improve the reconstruction accuracy of noisy images remains a critical task. Here, we integrate the prior information (edge map) from an LR image into the network to aid in the reconstruction. Although edge priors have been considered in realistic photograph restoration^33^, edge detection operators that are well suited for realistic photographs cannot be directly applied to microscopic images since the diffraction effect is not considered. As shown in Supplementary Fig. 4a, the edges extracted from the microscopic LR image by these operators fail to indicate the high-resolution structures in the GT image. Instead, we extracted a subpixel edge map from a microscopic image based on the radial symmetry of imaged fluorophores (Supplementary Fig. 4b) which has been utilized in super-resolution microscopy methods^34–36^. Inspired by Gustafsson et al.^34^, who analyzed the temporal cumulates in the radiality maps of a sequence of images to reconstruct one SR image, we computed the edge map from a single LR image (Supplementary Fig. 4c) and used it as an additional input to the network, which is proven to effectively improve the fine-structure reconstruction accuracy of the network from the noisy LR image (Supplementary Fig. 3, with the edge map).

We then wonder to what extent the network can maintain its performance in the presence of different levels of noise. To test this, we set the background and noise at a certain level and decreased the signal intensity to different levels to simulate a set of images with different SNRs as shown in Supplementary Fig. 5. After comparing the reconstruction results from inputs with different SNRs by visually inspecting the reconstruction quality (Supplementary Fig. 5a) and quantitatively analyzing the reconstruction accuracy (Supplementary Fig. 5b), we found that the network has a prerequisite for input SNR at about 15, which poses a challenge for applications in live-cell imaging where the signal level could be quite low due to short exposure and low illuminance. To address this challenge, we tried to improve the image SNR in advance. There are plenty of networks that could be used for denoising, such as RCAN^28^ and CARE^37^. Because the ESRGAN generator works well for denoising tasks with registered low-SNR (LSNR) and high-SNR (HSNR) data when trained with MS-SSIM-L1 loss and perceptual loss, we adopted another ESRGAN generator as SEN prior to SRN to progressively optimize the image SNR and resolution, and thus an SR image can be exquisitely restored from a low-SNR LR image (Fig. 1b). With the aid of SEN, the minimum SNR requirement of SFSRM could be extended to SNR of 7 (Supplementary Fig. 5, HSNR-SR), making it accessible to most live-cell applications (see Supplementary Note 1 for the SNR estimation in fluorescent imaging).

### SFSRM reconstructs a super-resolution image from a single diffraction-limited image

**Fig. 2:**
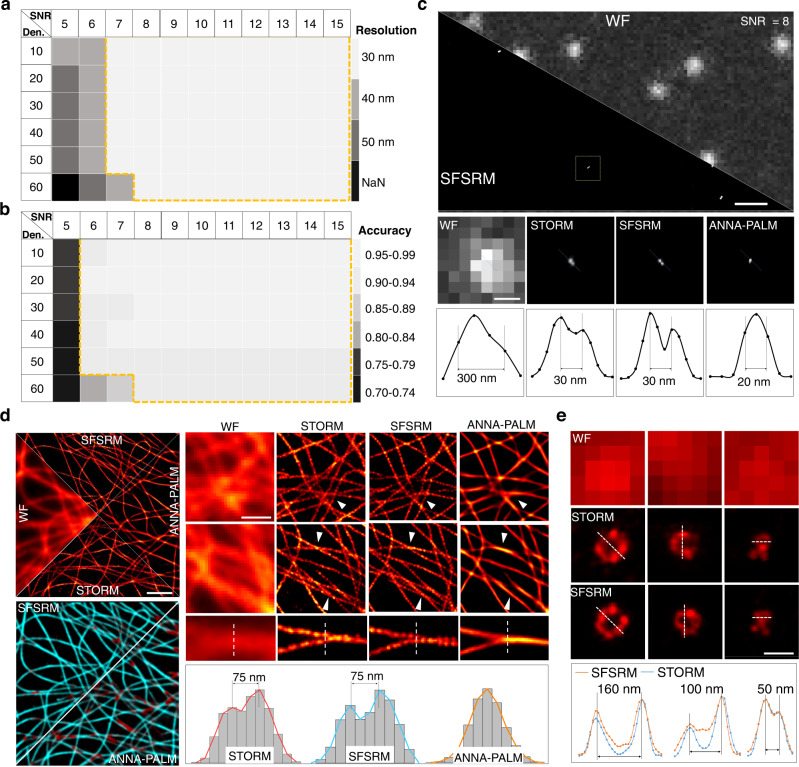
Overview performance of SFSRM. **a**, **b** The measured resolution and reconstruction accuracy of SFSRM on simulation line pairs at different signal densities (Den.) and SNRs. The orange dashed box indicates the applicable region of SFSRM. The corresponding reconstruction results in **a** and **b** can be found in Supplementary Figs. 7–11 and 13 respectively. **c** Validation of the SFSRM’s performance on standard DNA nanorulers with a 30-nm mark-to-mark distance. Top row: the WF image obtained from the Zeiss Elyra 7 microscope with HILO mode and the corresponding SFSRM reconstruction. Middle row: the zoom-in comparison of the WF, STORM, SFSRM reconstruction, and ANNA-PALM reconstruction of a single DNA nanoruler. The STORM image is obtained from 20,000 frames of single-molecule images. SFSRM and ANNA-PALM images are the reconstruction results from the WF image via SFSRM and ANNA-PALM network respectively. Both SFSRM and ANNAPALM networks and trained with the same simulation dataset. Bottom row: Intensity profiles along the lines indicated by the blue dashed lines in the images in the middle row. The measured FWHM PSF size in the WF image is ~300 nm, and the measured distances between the two spots in the STORM and SFSRM images are 30 nm. **d** Validation of the SFSRM’s performance on experimental images of immunostained microtubules. Top left: the WF image of microtubules in fixed Beas2B cells, the SFSRM reconstruction (top) the ANNAPALM reconstruction (right), and the corresponding STORM image (bottom). Bottom left: Confidence maps of the SFSRM and ANNA-PALM reconstruction measured by HAWKMAN analysis. The low-confidence region is marked in red and the high-confidence region in cyan. Top right: Zoom-in comparison of the WF, STORM, SFSRM, and ANNAPALM images. The white arrows indicate where the microtubules are missed in the ANNAPALM reconstruction. Bottom right: Intensity profiles along the lines indicated by the white dashed lines in the images in the middle row. **e** Representative SFSRM reconstruction results of clathrin-coated pits (CCPs) of different sizes. Scale bar: 1 µm (**c**, **d**), 200 nm (**e**, zoom-in view in **c**), 500 nm (zoom-in view in **d**).

Considering signal variations in density and intensity during the fluorescent imaging, we systemically evaluated the resolution and accuracy of SFSRM in a range of signal density and intensity on simulation line pairs. As shown in Supplementary Note 2 (Section I), we investigated the reconstruction accuracy of line-pairs with different interpair distances between 10 nm and 50 nm at different SNRs and signal densities and regarded the smallest interpair distance that can be resolved at an accuracy >0.85 as the best achievable resolution of the network (Supplementary Figs. 6–12). We summarized the achievable resolution of the network at different SNRs and signal densities in Fig. 2a which indicates that SFSRM can generally separate two lines that are 30 nm apart when the SNR of the LR image is above 7 and the signal density is <60%.

We further evaluated the network reconstruction accuracy considering more reconstruction errors such as missing/biased structures via HAWKMAN analysis^38^ which gives a HAWKMAN score indicating the overall structural cross-correlation between the SR and HR images, and a confidence map marking the low-confidence structures (Supplementary Note 2, Section II, Supplementary Figs. 13 and 14). We regarded the HAWKMAN score as the reconstruction accuracy of the network. Figure 2b shows that SFSRM can achieve an accuracy over 0.9 when the SNR of the LR image is above 7 and signal density is <60%; while for higher signal density over 60%, the network requires a higher input SNR to achieve comparable accuracy.

### SFSRM reconstructs a super-resolution image from a single experimental images of fixed cells

We then tried to validate the resolution of SFSRM on experimental images. DNA origami nanorulers are standard samples that have two fluorescent markers with a specified mark-to-mark distance. To test SFSRM on DNA origami nanorulers, we first simulated dot pairs with an interpair distance ranging from 20 nm to 50 nm randomly distributed in GT images. The GT images were then blurred by a Gaussian kernel of a 280-nm FWHM size and followed by applying Poisson noise and Gaussian noise to get the LR images. The results in Supplementary Fig. 15 show that SFSRM accurately reconstructs 46%, 80%, 82%, and 84% dot pairs of 20-nm, 30-nm, 40-nm, and 50-nm interpair distances from the indistinguishable spots in the LR image, the reconstruction bias within half of the interpair distance is 76%, 99%, 99%, and 99% respectively, suggesting a reliable highest resolution at ~30 nm, similar to our observation on the simulated line pairs. We then used the trained SFSRM network to process the experimental WF images of DNA origami nanorulers with a 30-nm mark-to-mark distance (Fig. 2c**)**. SFSRM clearly distinguished two spots and accurately reconstructed the distance between the two spots which is about 30 nm as measured from the STORM image. By contrast, a representative deep-learning-based super-resolution method called ANNA-PALM^31^ is only able to reduce the size of the spot while failing to reconstruct the dot pairs from the blurred spots in the WF image given the challenging SNR of the WF image (e.g., SNR ~8).

We then investigated the performance of SFSRM on the experimental images of subcellular structures. We first validated the effectiveness of the SFSRM method on experimental images of fixed microtubules. We collected training data (11 frames of STORM images with the corresponding WF images) of fixed microtubules stained with Alexa Fluor 647, and trained the network with different strategies. The results in Supplementary Fig. 16 suggest our approach can effectively improve the reconstruction resolution and reconstruction fidelity of fine structure compared to the basic ESRGAN generator trained with the pixel-wise loss (MS-SSIM-L1 loss). To test network robustness to different levels of experimental noise. A sequence of images of different SNRs was obtained and processed by the network. The reconstructed SR images were then compared with the corresponding STORM image by HAWKMAN analysis. The confidence maps indicate that the reconstruction errors increase as the SNR decreases (Supplementary Fig. 17, LSNR-SR confidence map). When only SRN is used, the HAWKMAN score falls below 0.8 for SNR < 15, indicating a less reliable reconstruction result (Supplementary Fig. 17b, LSNR-SR). By contrast, if SEN is used in combination with SRN, the input SNR limit can be extended to SNR > 7 (Supplementary Fig. 17b, HSNR-SR).

We compared the performance of SFSRM and ANNA-PALM on experimental images of microtubules in Fig. 2d. Although ANNA-PALM successfully reconstructs isolated microtubules, some of the microtubules are merged or lost in the reconstruction results where the microtubules are densely distributed (Fig. 2d, indicated by white arrows). In contrast, SFSRM correctly reconstructed most microtubules without losing or merging them even when they are close to each other. Quantitative assessment of the network reconstruction fidelity via HAWKMAN analysis demonstrates notably reduced local errors in the SFSRM reconstruction and on-average higher fidelity of the SFSRM reconstruction (HAWKMAN score: 0.95 vs. 0.90) (Fig. 2d, confidence maps). As depicted by the intensity profiles for the lines in Fig. 2d, two microtubules only 75 nm apart are indistinguishable in the ANNA-PALM reconstruction and are resolved in the SFSRM reconstruction result (Fig. 2d, plot), demonstrating a superior fine-structure reconstruction capability of SFSRM. In addition to our experimental data, SFSRM also achieves comparable reconstruction results to those via Deep-STORM^30^ on the public dataset from the EPFL SMLM challenge website^39^ (Supplementary Fig. 18). Unlike Deep-STORM which requires 300 frames of densely-distributed single-molecule images to reconstruct an SR image, SFSRM restores the SR image only from a single WF image, greatly reducing the photobleaching to the specimen as well as the data acquisition time. Apart from filaments, the performance of SFSRM on diverse subcellular structures is also promising. As shown in Fig. 2e, SFSRM resolves the ring-shaped clathrin-coated pits (CCPs) with diameters ranging from 50 nm to 160 nm from the noisy WF image. The estimated diameters of the CCPs from the SFSRM reconstructions show good consistency with that measured from the STORM images (Supplementary Fig. 19).

**Fig. 3:**
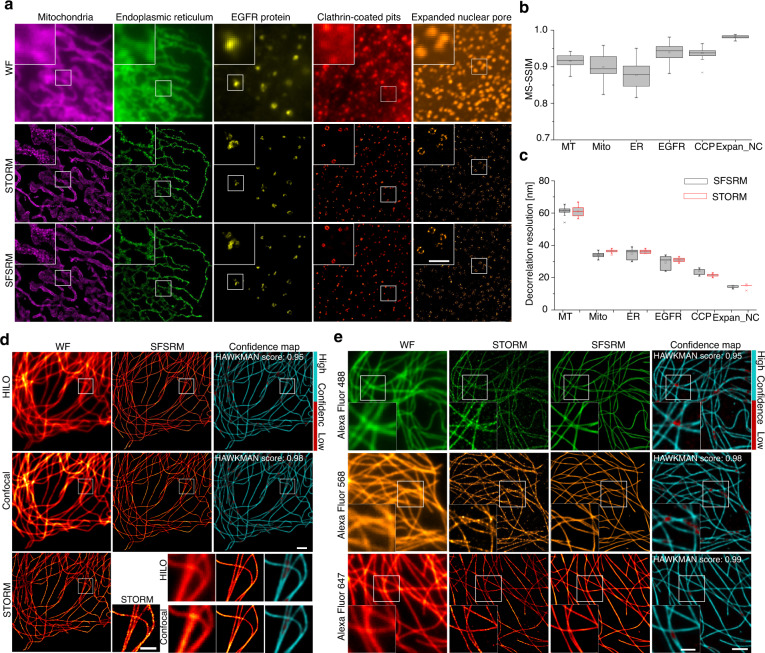
SFSRM applies to different subcellular structures, imaging systems, and spectra. **a** First row: representative WF images of mitochondria labeled with the mitochondrial membrane, endoplasmic reticulum (ER), EGFR protein after the EGF endocytosis, clathrin-coated pits after the EGF endocytosis, and expanded nuclear pore complex protein Nup133 (The specimen was expanded for 2.5 times with expansion microscopy after immunostaining). Second row: STORM images. Third row: corresponding SR images inferred from the WF images by SFSRM. **b** The reconstruction fidelity of SFSRM on different cellular structures measured by multi-scale structure similarity (MS-SSIM) index between the SFSRM and the corresponding STORM images. **c** The comparison of resolution (measured by decorrelation analysis) of SFSRM and the corresponding STORM images on different cellular structures. The error bars in **b** and **c** represent reconstruction experiments repeated on 25 images. All boxplots are drawn from the 25th to 75th percentile with the horizontal bar at the median and the whiskers extending to the minima and maxima. **d** The reconstruction results of WF images obtained from different imaging systems via SFSRM. The first column shows raw images obtained from a Zeiss Elyra 7 and the Zeiss sp8 confocal microscopes. Both WF images are processed by the SFSRM network to get the SR images in the second column. The SR images are compared with the STORM images and the differences are marked in the corresponding confidence maps in the third column. **e** The reconstruction results of WF images of microtubules separately labeled by dyes of different spectra. First column: WF images acquired from microtubules immunostained by Alexa Fluor 488, 568, and 647 separately. Second column: STORM images. Third column: SR images restored by the SFSRM network trained with images of microtubules stained by Alexa Fluor 647. Fourth column: confidence maps indicate the reconstruction errors in each SR image. The reconstruction results of the 488 and 568 channels have slightly lower HAWKMAN scores compared to that of the 647 channels, which might be caused by the inferior qualities of STORM images in the two channels. Scale bar, 2 µm (**a**, **d**, **e**), 1 µm (zoom-in view in **a**, **d**, **e**).

We further benchmarked the performance of SFSRM on more subcellular structures including mitochondrial outer membrane, endoplasmic reticulum (ER), epidermal growth factor receptor (EGFR) protein, and nuclear pore complex proteins post a 2.5-fold expansion (Fig. 3a). The reconstruction fidelity is measured by the MS-SSIM index of the SR images with respect to the STORM images, and the resolution is measured by decorrelation analysis^40^. SFSRM achieves in general an MS-SSIM score over 0.8 (Fig. 3b) and resolutions of different structures ranging from 15 nm to 40 nm, consistent with those obtained from the corresponding STORM images (Fig. 3c). In addition to the SR reconstruction of diverse organelles, SFSRM also demonstrates remarkable robustness to changes in imaging conditions including different imaging systems (Fig. 3d) and different spectra (Fig. 3e). Therefore, it serves as a versatile tool to transform different types of LR images to their SR counterparts by overcoming the limitations of SR microscopy such as requiring fluorophore blinking, long acquisition time, and high illuminance.

### SFSRM enables live-cell SR imaging at millisecond temporal resolution

**Fig. 4:**
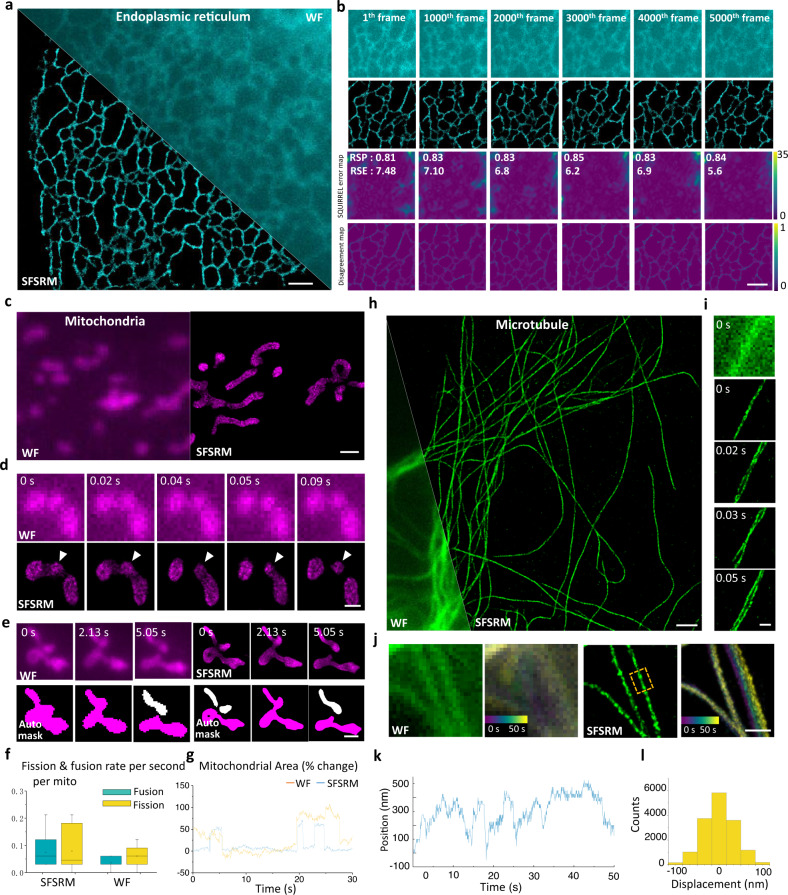
SFSRM enables noninvasive super-resolution imaging in live cells at millisecond temporal resolution for thousands of frames. **a** Representative WF and SFSRM images of endoplasmic reticulum in live Beas2B cells transfected with EGFP-Sec61β. **b** Time-lapse images of endoplasmic reticulum in live cells imaged with 15 W/cm^2^ intensity illumination for 5000 frames. First row: representative raw low-SNR images. Second row: the corresponding SFSRM reconstructions from the low-SNR images. Third row: Error maps of SFSRM reconstructions compared to the raw low-SNR images. The reconstruction errors are analyzed by SQUIRREL analysis, which gives an error map to indicate the possible local errors. Fourth row: disagreement maps measured by ensemble disagreement method, in which the regions with high disagreement score could be less trustworthy. **c** Representative WF and SFSRM images of mitochondria in live Beas2B cells transfected with Tom20-mcherry. **d** Comparison of the WF and corresponding SFSRM time-lapse images showing a mitochondrial “kiss-and-run” process. **e** Comparison of the mitochondrial segmentation results from the WF and SFSRM images. The target mitochondria in the segmentation results are marked in magenta while the other mitochondria are marked in white. **f** Comparison of the counted transient fusion and fission rates in the SFSRM and WF sequences. Boxplots are drawn from the 25th to 75th percentile with the horizontal bar at the median and the whiskers extending to the minima and maxima. Fusion and fission rates in 10 s were analyzed. **g** Comparison of measured mitochondrial area variation of the target mitochondrion in the SFSRM and WF sequences. **h** Representative WF and SFSRM images of microtubules in Beas2B cells expressing mEmerald-ensconsin. **i** Microtubule bundle instability caused by inconsonant fluctuations of microtubules. **j** Comparison of the WF and SFSRM images of the microtubules. The temporal-color-coded images, which are the maximum projected images of the time-lapse WF/SFSRM images with each frame rendered with different colors, indicate the microtubule transverse movement over time. **k** The transverse positions of a single microtubule over time recorded at 20 Hz. **l** Histogram of microtubule transverse displacement at a 50-ms interval. Scale bar, 2 μm (**a**–**c**, **e**, **h**), 500 nm (**d**, **i**, **j**).

Benefiting from its capability of reconstructing an SR image from a low-SNR LR image, SFSRM achieves SR imaging of ER in live cells at low illuminance (e.g., 15 W/cm^2^) (Fig. 4a), which allows long-term observation of ER dynamics for over 5000 frames without an apparent shrinking of the ER network or bleaching of fluorescent signals (Supplementary Movie 1). We assessed the reconstruction fidelity via resolution-scaled error analysis^41^ and network ensemble disagreement (see more details about disagreement analysis in Supplementary Note 2 (Section II). The resolution-scaled error analysis measures a resolution-scaled error and a resolution-scaled Pearson coefficient to indicate the correlation between the SR and WF images based on their intensity distribution. As shown in the error maps in Fig. 4b, no significant artifacts were found. Instead, we observed some errors in the upper corner of the error maps which gradually fade out. This might be caused by the non-linear mapping between the SR images and the WF images since STORM images which are regarded as the GT images of the network cannot preserve the intensity information in the WF images. In contrast to the error maps which indicates the apparent errors occurring in the upper corner, the disagreement maps suggest that some abnormally thin ER tubules in the reconstruction images could be problematic (Fig. 4b, disagreement map; Supplementary Fig. 20).

The enhancements in both spatial and temporal resolutions can promote the visualization of mitochondrial dynamics in live cells (Fig. 4c). The SR imaging of mitochondria at 100 Hz reveals frequent “kiss-and-run” interactions between mitochondria at the millisecond scale, which are indistinguishable in the LR time-lapse images (Fig. 4d and Supplementary Movie 2). Because of the high local signal density in mitochondria, it is necessary to check whether the observed fusion and fission events are true mitochondrial interactions or reconstruction artifacts. Therefore we investigated the network reconstruction consistency of the image sequence. We used the network to reconstruct a sequence of WF images of mitochondria in fixed cells and analyzed the consistency of the SR image sequence by calculating the pixel-wise agreement score (Supplementary Fig. 21a). Comparing the agreement map with the STORM image, no obvious reconstruction errors are detectable at the junctions where the mitochondrial connections are solid (Supplementary Fig. 21b, solid connections). However, reconstruction errors occur at junctions with ambiguous connections, which can further induce artificial fusion/fission events. Fortunately, these junctions can be detected by the agreement map (Supplementary Fig. 21b, ambiguous connections). This suggests that we can use the agreement map to detect the problematic junctions in the SR images. However, it is not feasible to acquire multiple WF images of the same sample to calculate the agreement map in live-cell imaging. Hence we used the five adjacent frames in the SR time-lapse images to check the temporal consistency (Supplementary Fig. 22a), which helps to filter out structures with low agreement scores in the five adjacent frames. From the filtered SR time-lapse images, we observed a higher transient fission and fusion frequency in the SR image sequence compared to those observed from the LR image sequence (Fig. 4f). Besides, the mitochondrial morphological changes can also be more precisely quantified with the aid of SFSRM. Figure 4e indicates the mitochondria undergo diverse morphological changes; however, these changes can barely be detected in the LR time-lapse images (Supplementary Movie 2). Clear tracking of the mitochondrial morphological changes enabled by SFSRM improves the segmentation accuracy and discloses a more rapid mitochondrial area change (Fig. 4g) that might be associated with the transient fusion which is reported to enhance the functional stability and plasticity of mitochondria^42^.

Real-time SFSRM imaging has also revealed some dynamics of microtubules that were unexplored in previous studies. Compared to the wide-field imaging result, the tangled microtubule’s network is more clearly resolved in the SR image with various morphologies such as bending, crossing, and bundles (Fig. 4h, SR; Supplementary Movie 3, part I). Besides, deformation dynamics of microtubules, such as bending (Supplementary Fig. 23a and Supplementary Movie 3, part II), growth and shrink instability (Supplementary Fig. 23c and Supplementary Movie 3, part II), are recorded at high temporal resolution (10-ms intervals), which allows us to capture high-frequency fluctuations including the time-varying bending (Supplementary Fig. 23b; 100 Hz) and the random-walk growth trajectory (Supplementary Fig. 23d; 100 Hz). These results suggest that the intracellular dynamics at the millisecond scale may be greatly underestimated at a low sampling frequency (Supplementary Figs. 23b,d; 2 Hz). Moreover, we also noticed the intracellular transverse fluctuation of microtubules from the temporal-coded image of the SR image sequence, which is undetectable in the LR counterpart (Fig. 4j). We observed the transverse position of the microtubule varies in an ~600 nm range (Fig. 4k and Supplementary Movie 3, part III), which are composed of displacements ranging from −100 nm to 100 nm at 50 ms intervals (Fig. 4l), far larger than the system drift (<10 nm in 50 s; Supplementary Fig. 24a). To figure out whether these displacements are true microtubule vibrations or noise-induced reconstruction misplacements, fixed microtubules were imaged at different illumination intensities to get time-lapse images at different SNRs. The statistical results in Supplementary Fig. 24b indicate that only less than 18% of microtubules present noise-induced reconstruction misplacements and these misplacements are within the ±25 nm range when SNR is >7. However, in the SFSRM reconstructed live-cell image sequences, we observed ~46% displacements in the ±25 nm range and ~18% displacements in ±25 ~ 100 nm. Besides, we also observed that the microtubule can rapidly move towards one direction in a short time as shown in Supplementary Fig. 24c and the distributions of the displacements shift toward that direction correspondingly, suggesting that true microtubule fluctuations rather than random reconstruction misplacements were present in our SFSRM live-cell imaging. The rapid and random fluctuations of microtubules could further cause the local microtubule’s network morphology changes such as bundle instability (Fig. 4i), which may be involved in multiple cellular functions, such as organizing and maintaining cell shape^43^, promoting cilia movement^44^, and modulating cargo transport^45^.

### SFSRM reveals the millisecond dynamics of cargo trafficking in live cells

Intracellular transport plays an essential role in maintaining cellular functions. Many cellular processes rely on the transport system to deliver proteins or organelles to a specific functional location. External cargos such as viruses and nanoparticles also utilize the transport system to deliver their genomes or drugs to specific compartments for function^46^. Considering that the cytoplasm of eukaryotic cells is highly crowded and dynamic, how cargo is delivered across the cytoplasm to specific positions remains largely unclear. Previous studies have reported that microtubules serve as highways to deliver cargo between the perinuclear region and the cell periphery in rapid and directed motions involving motor proteins^47^. Recently, facilitated by single-particle tracking techniques^48,49^, mounting dynamic behaviors during the cargo transport process, e.g., back-and-forth movement, rotation, pause, and switching direction, have been discovered, suggesting that rapid and directed motions are frequently interrupted. Some in vitro studies have suggested that the intersections of the microtubules are likely to interfere with cargo transport and form tethering points for cargo^45^. Single-particle tracking combined with confocal microscopy or STORM microscopy has also been employed to investigate vesicle behavior at microtubule intersections in live cells^48–50^. However, confocal microscopy fails to provide a high-resolution microtubule map, while STORM requires the sequential imaging of vesicles and microtubules. In addition, to register the vesicle trajectory along microtubules in STORM images, microtubule dynamics are stabilized by paclitaxel and nocodazole during live-cell imaging^50^. The lack of real-time high-resolution microtubule imaging has impeded the further exploration of cargo-microtubule interactions. Hence, the underlying mechanism of the complicated dynamics of vesicular trafficking along microtubules remains largely unknown.

**Fig. 5:**
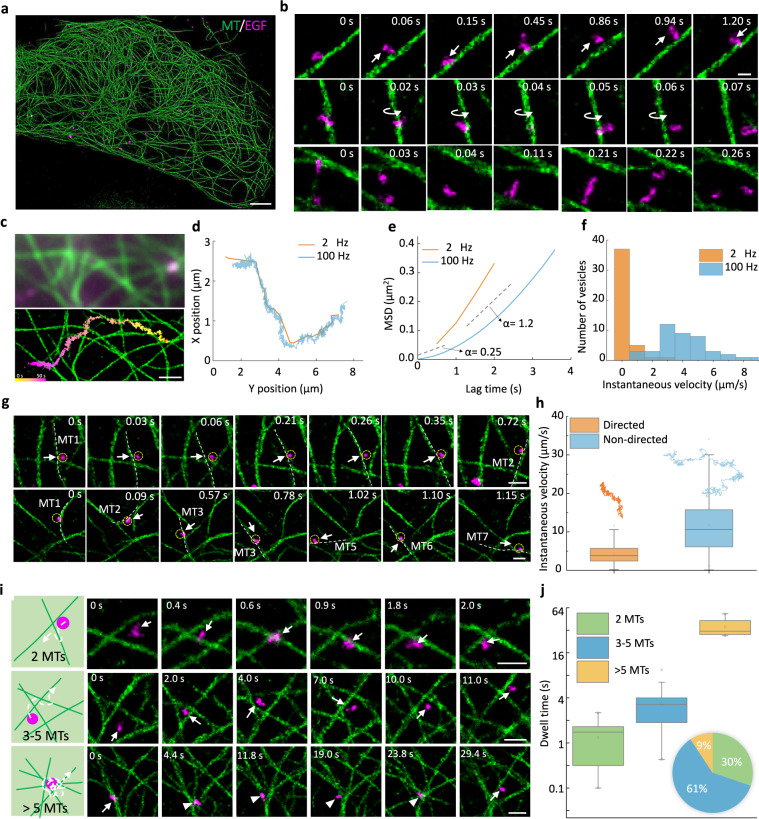
Dual-color real-time SFSRM imaging reveals the microtubule-vesicle interactions. **a** Representative dual-channel image of microtubules (green) and vesicles (magenta) in Beas2B cells after Epidermal Growth Factor (EGF) protein endocytosis. **b** Examples of vesicle transport dynamics. First row: moving back and forth along a microtubule; Second row: moving around a microtubule along a sinusoidal-like trajectory; Third row: colliding with another vesicle. **c** Comparison of the dual-color LR and SR images. The trajectory in SR image shows the motions of vesicle along microtubule at millisecond scale. **d** Comparison of trajectories recorded at 2 Hz and 100 Hz. **e** Mean-squared displacement (MSD) analysis of trajectories in **d**. MSD reflects the mean-squared-distance (∆*r*^2^ (*τ*)) of the vesicle traveled in a certain lag time *τ*, which typically follows the power-law trend 〈∆*r*^2^ (*τ*) 〉∝*τ*^α^. *α* indicates the characteristic of the motion. A smaller *α* indicates a more random or diffusive motion while a larger *α* indicates a more directed motion. **f** Statistical comparison of vesicle instantaneous velocity recorded at 2 Hz and 100 Hz. **g** Examples of microtubule dynamics resulting in nondirected transport of vesicles. First row: an example showing transverse movements of a microtubule deliver the vesicle to a nearby microtubule. Second row: example showing random fluctuations of surrounding microtubules facilitate the vesicle to switch to different microtubules. **h** The statistical comparison of the instantaneous velocity of the directed/nondirected transport and their representative trajectories. An image sequence containing 800 frames of images was used to analyze the instantaneous velocity. **i** Vesicle transport dynamics at different types of microtubule intersections. **j** The statistical comparison of the dwell time at different types of intersections and their percentages in cells (pie chart). 43 intersections were analyzed. Boxplots are drawn from the 25th to 75th percentile with the horizontal bar at the median and the whiskers extending to the minima and maxima. Scale bar, 2 µm (**a**); 200 nm (**b**); 1 µm (**c**); 500 nm (**g**, **i**).

Here, benefiting from the high spatiotemporal resolution offered by SFSRM, we can simultaneously monitor the cargo and microtubule dynamics, thereby investigating how the observed microtubule dynamics could affect cargo transport. As a demonstration, we imaged the intracellular transport of the endocytic trafficking of epidermal growth factor (EGF) protein in live cells. The internalization of EGF was recorded with dual-channel SFSRM (Fig. 5a and Supplementary Movie 4, part I). High-spatiotemporal-resolution videometry reveals the vesicle transport details, from which we noticed that slight fluctuations of a single microtubule do not interrupt the directed transport of vesicles, but the motions of the vesicles along the fluctuating microtubules are significantly more dynamic than expected. Figure 5b illustrates three examples of vesicle transport dynamics: (I) moving back and forth along a microtubule, (II) moving around a microtubule along a sinusoidal-like trajectory (Supplementary Fig. 25), and (III) colliding with other vesicles and then changing direction (Supplementary Movie 4, part II). These subtle and fast random walks are undetectable at low spatial (Fig. 5c) or temporal (Fig. 5d) resolutions (Supplementary Movie 4, part II), which implies that vesicle movement is scale-dependent. At the millisecond scale, thermal diffusion is dominant (Fig. 5e, 100 Hz, *α* = 0.25), and at the second scale, directed transport dominates (Fig. 5e, 100 Hz, *α* = 1.2)^51^. At inadequate imaging speeds, these diffusive motions would have been missed, as manifested by the distinct trajectories derived by the images taken at 2 Hz and 100 Hz shown in the MSD plot (Fig. 5e, 2 Hz vs. 100 Hz). Consequently, the actual instantaneous velocity of vesicles during transport, which is ~4 µm/s (Fig. 5f, 100 Hz), would have been substantially underestimated (estimated as ~0.5 µm/s in Fig. 5f at 2 Hz, which is in accordance with a previous report^49^).

In addition to these subtle diffusive motions, we also observed nondirected transport, which has been reported in previous studies using single-particle tracking (SPT) but not fully explained^49,52^. Compared to SPT, our SFSRM can not only precisely determine the vesicle positions following its moving trajectory (Supplementary Fig. 26), but also resolve the vesicle morphology and orientation, thus enabling the study of the interaction of vesicles with their surroundings in a dynamic process. From the dual-channel video via SFSRM (Supplementary Movie 4), we observed some microtubule dynamics that might contribute to nondirected vesicle transport. For example, the transverse movement of a microtubule can transfer the vesicles attached to it to a nearby microtubule (Fig. 5g, first row, and the fluctuations in the surrounding microtubules can cause vesicles to switch among different microtubules (Fig. 5g, second row), resulting in nondirected transport (Supplementary Movie 4, part III). We compared the instantaneous velocities of directed transport and nondirected transport in Fig. 5h and found that nondirected movements have an ~2-fold higher average instantaneous velocity and a four-fold broader range of distribution than directed movements, indicating that these displacements are likely related to microtubule fluctuations rather than motor-driven movement. This observation is in good agreement with the results of a previous study by Giannakakou et al.^53^, who reported that the suppression of microtubule dynamics enhanced nuclear-targeted cargo P53 accumulation near the cell nucleus. More interestingly, the movement of vesicles can in turn contribute to the microtubule morphology change (Supplementary Fig. 27).

Since microtubules are densely distributed, aside from providing tracks for vesicles, they also form intersections that may interrupt vesicle transport. Previous studies have shown that vesicles may pass, pause, switch, or reverse at an intersection^45,50^. In our experiments, we noticed that all vesicles eventually passed the observed intersections; however, the dwell time varied greatly and largely depended on the complexity of the intersection. To further quantify how the complexity of the intersection would affect the vesicle transport, we first assessed the accuracy of our network in reconstructing the microtubule network morphology. As shown in Supplementary Fig. 28, we quantitatively analyzed the reconstruction errors which may affect the identification of intersections, for example, reconstructing an artificial microtubule (false positive error) or missing a microtubule (false negative error) (Supplementary Fig. 28b), false positive or negative intersections, or wrong microtubule number at the intersection (Supplementary Fig. 28c), as a function of signal density. As expected, the false rate increases as the signal gets dense. To ensure the reconstruction error rate is smaller than 15% (corresponding to a HAWKMAN score >0.8), we select regions with signal density <50% for the following analysis. The intersections in the selected regions are classified into three groups based on the number of microtubules at each intersection (Fig. 5i). For the simplest intersections of two microtubules, the vesicle can easily pass through it by climbing over one microtubule, usually within two seconds; and the microtubule vibration is unlikely to interrupt vesicle transport. For intersections with 3–5 microtubules, the vesicles tended to interfere with the dynamics of nearby microtubules. Thus, if the surrounding microtubules fluctuate severely, the vesicles are hindered, and the time needed to pass through these intersections ranged from several to ten seconds. For intersections involving more than 5 microtubules tethered together, the vesicles were most likely to be trapped at the intersection until the fluctuations of the surrounding microtubules became coordinated and the stellate intersection loosened. However, coordinated fluctuations and intersection loosening are highly uncertain, and such processes may take tens of seconds to minutes Supplementary Movie 4, part IV). A statistical comparison of the dwell time at different kinds of intersections is shown in Fig. 5j. Generally, the more complex the intersection is, the longer the resulting dwell time. For intersections involving more than 5 microtubules, the dwell time could be longer than one minute. Fortunately, this kind of intersection only accounts for ~9% of all intersections in a cell, whereas more than half of the intersections consist of only 3–5 microtubules (Fig. 5j, pie chart).

### SFSRM is robust to different imaging systems and different samples

**Fig. 6:**
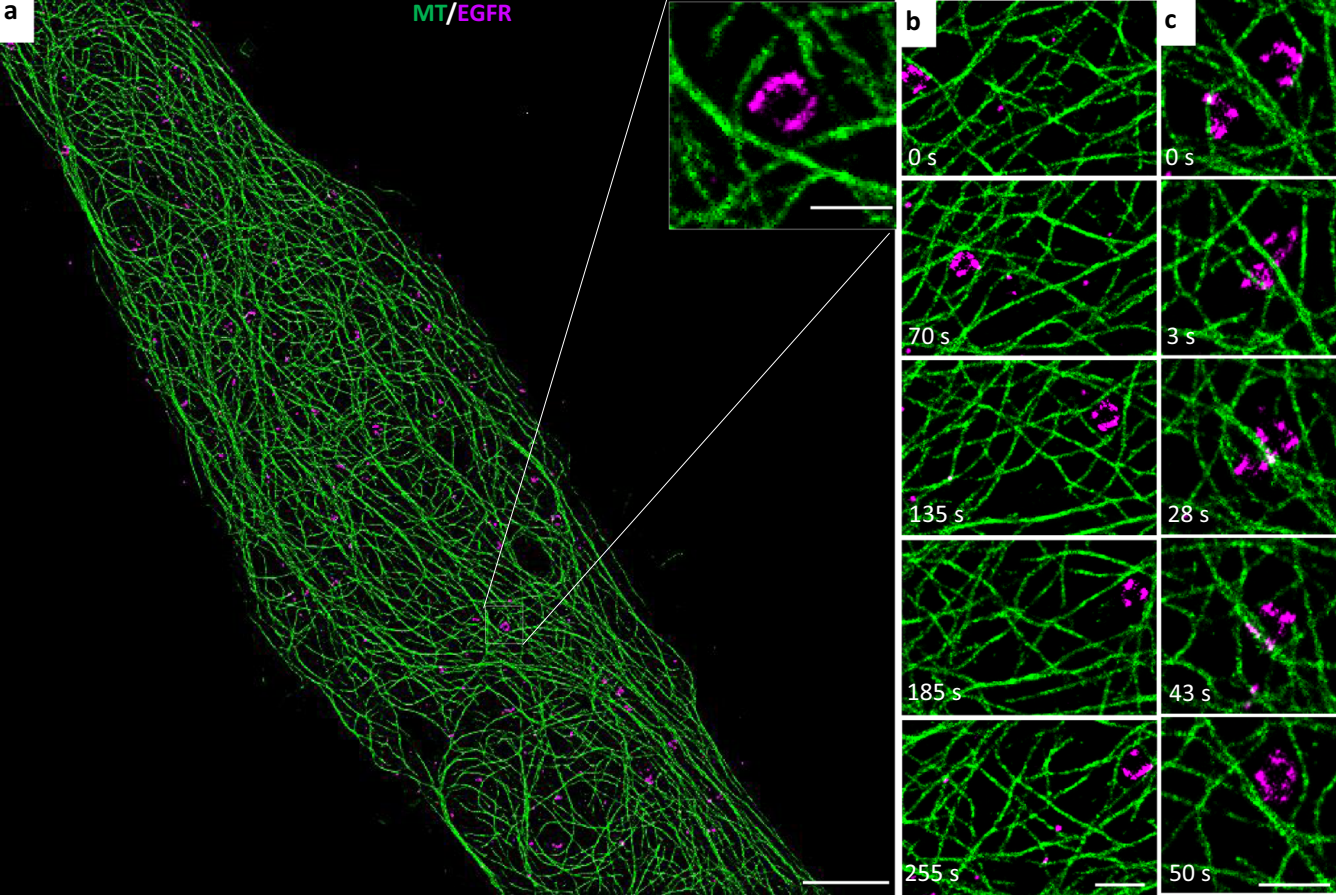
SFSRM imaging allows long-time-scale observation. **a** SFSRM reconstruction of a confocal image of microtubules (green) and EGFR-carrying vesicles (magenta) in Beas2B cells. Inset: an endosome accumulated with EGFR proteins is trapped in a local microtubule grid. **b** Example of the transport process of a trapped endosome. **c** Example of the fusion process of trapped endosomes. Scale bar, 5 µm (**a**), 500 nm for insets; 1 µm (**b**, **c**).

In the above demonstration, we used a Zeiss Elyra 7 microscope as the live-cell imaging system. Here, we demonstrate that SFSRM can also be applied in different live-cell imaging systems without retraining the networks. We validated the robustness of our network based on a commercial confocal microscope (Confocal sp8, Zeiss). Compared to that used for WF imaging, a confocal microscope requires a longer time (2.5 s/frame) to obtain a dual-channel image due to the point scanning strategy used. Here, we recorded the EGF receptor (EGFR) protein transport dynamics at 0.4 Hz for over 10 min after EGF treatment (Supplementary Movie 5, part I). Long-term observation allows us to discover some long-time-scale phenomena. For example, as shown in Fig. 6a, the EGFR protein gradually accumulates in endosomes, which appear as ring structures in the image. We noticed that the microtubules tended to generate local grids to trap the endosomes, as shown in the zoomed-in view in Fig. 6a. These traps will actively participate in the transport (Fig. 6b) and fusion processes of the endosomes (Fig. 6c), and the morphology of these grids will dynamically change in response to the endosome shape (Supplementary Movie 5, part II).

**Fig. 7:**
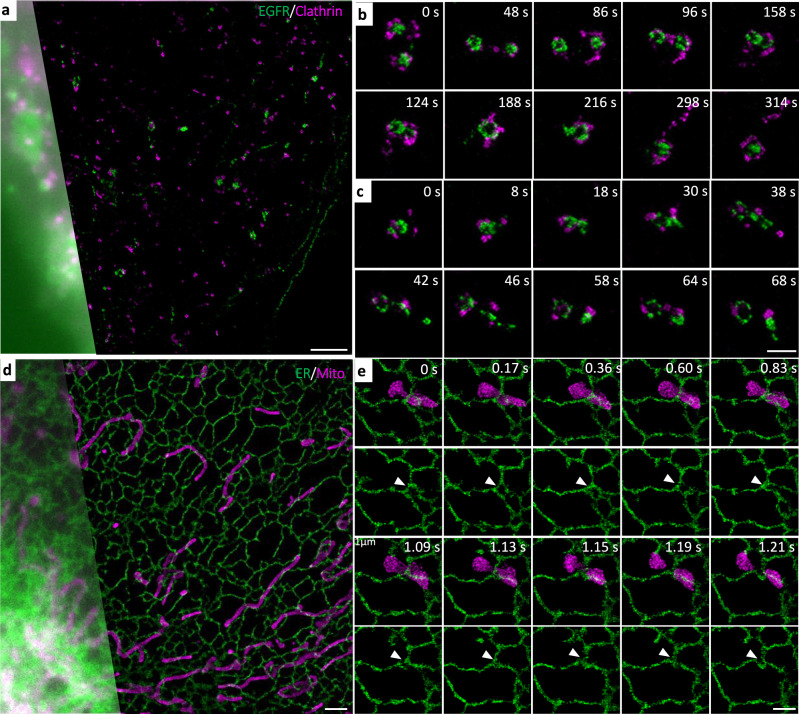
Dual-color real-time SFSRM imaging reveals subcellular dynamics of diverse organelles. **a** Representative dual-channel image of EGFR protein (green) and clathrin protein (magenta) in Beas2B cells expressing EGFR-EGFP and Halo-clathrin. **b** Example of the fusion process of two clathrin-coated endosomes. **c** Example of the endosome fission process. **d** Representative dual-channel image of ER (green) and mitochondria (magenta) from cells expressing Tomm20-mCherry, EGFP-Sec61β. **e** Example of the mitochondrial fission at the ER-mito contact site. White arrows indicate the ER-mito contact site. Scale bar, 2 µm (**a**, **d**), 1 µm (**b**, **c**, **e**).

SFSRM can be also applied in monitoring different live-cell dynamic processes involving various subcellular structures. For example, Fig. 7a shows the colocalization of clathrin protein and EGFR protein after EGF treatment, indicating the role of clathrin protein during EGF endocytosis. In addition to mediating the endocytosis process of EGF by generating ring-shaped CCPs (Supplementary Fig. 29; Supplementary Movie 6), clathrin protein is also recruited to endosomes that are larger than endocytic vesicles (Fig. 7b). During the fusion processof two adjacent endosomes, the membranes of endosomes that are uncoated with clathrin fused with each other. After the two endosomes are fully-fused, the extra clathrin protein is released from the fused endosomes (Supplementary Movie 6). The function of the clathrin protein on endosomes has been reported in cargo sorting^54^. After being delivered to the early endosomes, some of the endocytic EGFR will be sorted to tubular structures to be retrieved back to the cell surface^55^. As demonstrated in Fig. 7c, we detected that the endocytic EGFR in endosomes was sorted to the tubular membranes on endosomes and then vesicles enriched with EGFR were generated via the fission of tubular membranes (Supplementary Movie 6).

Besides, SRSFM also reveals the intensive contact between mitochondria and endoplasmic reticulum (ER) (Fig. 7d and Supplementary Movie 7) such as mitochondrial fission at the ER-mitochondria contact site^56^ (Fig. 7e), mitochondrial growth, and branch along the ER tubules, as well as the ER tubule hitchhiking on a moving mitochondria^57^ (Supplementary Fig. 30 and Supplementary Movie 7). These subtle yet fast interplays between different organelles, which could happen within a second, can be revealed by SFSRM, manifesting the high spatiotemporal resolution of SFSRM will make vital contributions to biological sciences involving live-cell dynamic processes.

## Discussion

We developed an SFSRM method for single-frame SR reconstruction from the LR images acquired from live cells with up to 10-fold resolution improvement. We have demonstrated that SFSRM restores subtle structures from LR images owing to the adoption of multicomponent loss and shows superior robustness to the corruption of noise with the assistance of the edge map. Besides, by employing a dual subnet framework that progressively improves the image SNR and resolution, the single-frame image conversion of SFSRM circumvents the possible tradeoffs among the spatial resolution, imaging speed, and light dose in the SR microscopies and allows long-term SR imaging in live cells without inducing noticeable photodamage to the cells. The coupling of SFSRM with different live-cell imaging systems improves the spatiotemporal resolution of fluorescence microscopes with a limited photon budget, thus serving as a powerful tool combining merits of the super-resolution microscopy and real-time live-cell imaging.

Nonetheless, as with all other learning-based methods, SFSRM faces the accuracy concern. Although it achieves an on-average higher accuracy than previous methods (Fig. 2 and Supplementary Figs. 16 and 18), SFSRM still has local reconstruction errors. Therefore, we have performed careful inspections of SFSRM reconstructions comparing to their corresponding GT images on the fixed samples based on multiple metrics including reconstruction bias, MS-SSIM, and HAWKMAN score. From our observations, the reconstruction accuracy of SFSRM shows dependency on the SNR and signal density of the input image (Supplementary Note 2, Supplementary Figs. 5, 7–14, 17). It achieves a generally satisfactory accuracy (mean reconstruction bias <1 pixel, HAWKMAN score >0.8, error rate <0.15) when the input SNR is >7 and the signal density is ≤0.5. While in live-cell applications where the GT images are inaccessible, we have tried assessments based on the similarity with the LR images (Fig. 4b) and the reconstruction uncertainty analysis of noise ensemble images of the same sample, network ensembles, and adjacent frames in live-cell imaging (Supplementary Note 2 and Supplementary Figs. 20–22). Considering SFSRM is robust enough for different spectra and imaging systems (Fig. 3d, e), we believe our prior validation of SFSRM on different SNRs, signal densities, and structures on fixed samples, combined with the quality check methods in live-cell imaging, can make it a practical tool in live-cell super-resolution imaging. In addition to these on-average accuracy evaluations, we also suggest custom-designed evaluations for specific applications. For example, when we were investigating the microtubule’s transversal fluctuations, we validated the pixel-level reconstruction consistency of SFSRM under different SNRs on the fixed cells in advance (Supplementary Fig. 24); and when we were studying the influence of the microtubule intersections on the vesicle transport, we quantified the precision of SFSRM for detecting the correct number of microtubules at each intersection beforehand (Supplementary Fig. 28).

The implementations of SFSRM with common fluorescent microscopes have allowed high-frequency transverse vibration of microtubules and surprisingly dynamic behaviors of vesicles such as diffusive motions along microtubules, swinging on microtubules, and switching between microtubules to be clearly resolved. Many of these processes, which have not been seen before, enhance our understanding of the real intracellular transport environment. Moreover, other subcellular processes revealed by SFSRM such as clathrin-endosome colocalization and ER-mitochondria interactions, suggest the potential of SFSRM in promoting the investigation of subcellular processes that necessitate interpreting temporal dynamics in the context of ultrastructural information, which may open doors to discoveries in live-cell imaging involving organelle dynamics^58^ and interactions^59^.

Overall, we consider SFSRM a useful alternative to traditional SR microscopies in challenging conditions such as low illuminance, short acquisition time, or multi-channel SR imaging. Its success lies in the use of adequate training data obtained for advanced SR microscopies. Considering these high-cost SR imaging systems are not ubiquitous in most biological laboratories, we hope SFSRM can make the most of the available SR datasets and serve more researchers. Nevertheless, in a realistic experimental setting, the risks that structural features presented in the experimental images do not match the training dataset range cannot be precluded. For example, the curvatures of the microtubules or the diameters of the clathrin-coated-pits are outside the curvature/diameter range of the training dataset, or cruder mismatch such as the network trained by endoplasmic reticulum being used to process the images of microtubules. From our observation in Supplementary Note 3**(**Supplementary Figs. 31–33**)** and Supplementary Fig. 34, the SFSRM network is not able to handle such scenarios. A convenient way to address this problem is to fine-tune the trained network using matched training dataset, which helps the network quickly adjust to a different structure feature space.

Beyond our approach, our observation that the network can reconstruct the SR image from a 10-fold blurred LR image without any difficulty while reconstructing a noise-corrupted image will need much more effort such as the edge map assistance and the multicomponent loss function, suggests improving the SNR of the network input and introducing prior regulations (e.g. total variation prior^60^, sparse prior^61^) are useful strategies to improve the reconstruction quality and could be further investigated to improve the performance of other networks.

## Methods

### SFSRM network

#### Network architecture

The networks of our SFSRM, including the SEN and SRN, are based on the ESRGAN generator^25^, which includes 23 residual-in-residual dense blocks used to map low-resolution images to super-resolution images. By inheriting the basic architecture of SRGAN^24^, this network performs most computations in the LR feature space, hence reducing complexity and achieving high stability without requiring batch normalization (BN) layers^25^. The original ESRGAN is designed for a single RGB image. When it was applied to a grayscale image, we found that the network will easily crash at the beginning of or during the training process if we duplicate the grayscale image three times to generate a fake RGB input. Therefore, we adopted a single-channel ESRGAN generator. To incorporate the prior information provided by the edge map, we added another input channel to the network. The input LR image and the corresponding edge map are initially concatenated to generate a two-channel input to the generator. Similarly, duplicated grayscale images are used as fake RGB inputs to the well-trained VGG network^62^ for feature map extraction.

#### Loss functions

To generate high-resolution details while maintaining high fidelity, the network is trained with a multicomponent loss function, as follows:

1. Content loss evaluates the *L*_1_-norm distance between an estimated SR image ${{{{{\rm{G}}}}}}\left(x\right)$ and a GT image $y$. *L*_1_-norm loss focuses on pixel differences, thus allowing the network to quickly converge but often resulting in a blurred image.
  1 $${L}_{1}={{{{{\rm{||G}}}}}}\left(x\right)-y{{{{{\rm{||}}}}}}$$
  MS-SSIM measures the structural similarity of SR and GT images based on luminance, contrast, and structure at different scales. The computation of MS-SSIM is detailed in the assessment metrics section. Here, we focus on the construction of the loss function. MS-SSIM loss is defined as:
  2 $${L}_{{{{{{\rm{MS}}}}}}-{{{{{\rm{SSIM}}}}}}}=1-{{{{{\rm{MS}}}}}}-{{{{{\rm{SSIM}}}}}}\left({{{{{\rm{G}}}}}}\left(x\right),y\right)$$
  Content loss is a hybrid of MS-SSIM loss and *L*_1_-norm loss and is noted as MS-SSIM-L1 loss:
  3 $${L}_{{{{{{\rm{MS}}}}}}-{{{{{\rm{SSIM}}}}}}-{{{{{\rm{L}}}}}}1}=\alpha \cdot {L}_{{{{{{\rm{MS}}}}}}-{{{{{\rm{SSIM}}}}}}}+(1-\alpha )\cdot {L}_{1}$$
  where $\alpha$ is used to balance the contributions of MS-SSIM loss and ${L}_{1}$-norm loss and is empirically set as $\alpha=0.84$^63^.
2. Perceptual loss ${L}_{{{{{{\rm{Percep}}}}}}}$ is used to measure feature distance differences in the estimated SR image and corresponding GT image. Features are extracted by a VGG network^62^ pretrained for material recognition and that is good at texture extraction.
  4 $${L}_{{{{{{\rm{Percep}}}}}}}={{{{{\rm{||F}}}}}}\left({{{{{\rm{G}}}}}}(x)\right)-{{{{{\rm{F}}}}}}(y){{{{{\rm{||}}}}}}$$
  where F represents the feature extraction network.
3. Adversarial loss estimates the probability that the discriminator input $x$ is real or fake. Here we use U-net as the discriminator, which has an encoder and a decoder. The discriminator is trained to provide both global and pixelwise decisions on whether the input image is real or fake^64^. Specifically, an input real image $y$ or fake image ${{{{{\rm{G}}}}}}(x)$ will be first gradually convolved by the encoder to one pixel to get a global decision on whether this image is real or fake, then the input will be gradually deconvolved by the decoder to its original size to get a per-pixel decision on whether this pixel is real or fake. The encoder and decoder are trained by the following losses:
  5 $${L}_{{{{{\rm{enc}}}}}}=-{{{{{\rm{E}}}}}}\left[{\log }{D}_{{{{{\rm{enc}}}}}}\left( y \right)\right]-{{{{{\rm{E}}}}}}\left[{\log }(1-{D}_{{{{{{\rm{enc}}}}}}}\left({{{{{\rm{G}}}}}}(x)\right))\right]$$
  6 $${L}_{{{{{{\rm{dec}}}}}}}=-{{{{{\rm{E}}}}}}\left[\mathop{\sum}\limits_{i,j}{\log }{\left[{D}_{{{{{{\rm{dec}}}}}}}\left(y\right)\right]}_{i,j}\right]-{{{{{\rm{E}}}}}}\left[\mathop{\sum}\limits_{i,j}{\log }(1-{\left[{D}_{{{{{{\rm{dec}}}}}}}\left({{{{{\rm{G}}}}}}(x)\right)\right]}_{i,j})\right]$$
  where ${D}_{{{{{{\rm{enc}}}}}}}\left( \cdot \right)$ is the encoder decision of the whole input and ${\left[{D}_{{{{{{\rm{dec}}}}}}}\left(\cdot\right)\right]}_{i,j}$ is the decoder decision at pixel $\left(i,\, j\right)$; E[·] represents taking the average for all data in the minibatch. The discriminator is trained by both encoder loss and decoder loss.
  7 $${L}_{D}={L}_{{{{{{\rm{enc}}}}}}}+{L}_{{{{{{\rm{dec}}}}}}}$$
  Correspondingly, the discriminator feedback to the generator, i.e., the adversarial loss is formulated as
  8 $${L}_{{{{{{\rm{Adv}}}}}}}=-{{{{{\rm{E}}}}}}[{\log }{D}_{{{{{{\rm{enc}}}}}}}\left({{{{{\rm{G}}}}}}(x)\right)]-{{{{{\rm{E}}}}}}\left[\mathop{\sum}\limits_{i,j}{\log }{\left[{D}_{{{{{{\rm{dec}}}}}}}\left({{{{{\rm{G}}}}}}(x)\right)\right]}_{i,j}\right]$$
4. Frequency loss compares the frequency difference between an estimated SR and the original GT image:

9 $${L}_{{{{{{\rm{Freq}}}}}}}={{{{{\rm{||FFT}}}}}}\left({{{{{\rm{G}}}}}}(x)\right)-{{{{{\rm{FFT}}}}}}(y){{{{{\rm{||}}}}}}$$

where FFT is the fast Fourier transformation function. We compared all frequency components when the GT images do not contain noise and 75% of frequency components when the GT images contain noise, for experimental images as well as some simulation images.

When using the ESRGAN as SEN for signal enhancement, the network only uses LR images as single-channel inputs. The training of the SEN includes two steps:

1. Training with MS-SSIM-L1 loss for ~100,000 minibatch iterations at a 3 × 10^−4^ learning rate
  10 $${L}_{{{{{{\rm{G}}}}}}}={L}_{{{{{{\rm{MS}}}}}}-{{{{{\rm{SSIM}}}}}}-{{{{{\rm{L}}}}}}1}$$
2. Training with MS-SSIM-L1 loss and perceptual loss for 20,000 to 50,000 minibatch iterations at a 1 × 10^−4^ learning rate.

11 $${L}_{{{{{{\rm{G}}}}}}}={L}_{{{{{{\rm{MS}}}}}}-{{{{{\rm{SSIM}}}}}}-{{{{{\rm{L}}}}}}1}+{\delta \cdot L}_{{{{{{\rm{percep}}}}}}}$$

where $\delta$ is the coefficient to balance different loss components and we empirically set $\delta=0.1$.

When using the ESRGAN as SRN for super-resolution restoration, the network uses both LR images and edge maps as inputs. The training process also includes two stages. The first stage uses the same loss function as the SEN, and the second stage uses the following loss function with a 5 × 10^−5^ learning rate for ~10,000 minibatch iterations.

12 $${L}_{G}={L}_{{{{{{\rm{MS}}}}}}-{{{{{\rm{SSIM}}}}}}-{{{{{\rm{L}}}}}}1}+{\delta \cdot L}_{{{{{{\rm{Percep}}}}}}}+\beta \cdot {L}_{{{{{{\rm{Adv}}}}}}}+\gamma \cdot {L}_{{{{{{\rm{Freq}}}}}}}$$

In our experiments, we empirically set $\delta$, $\beta$, $\gamma$ and to $\delta=0.1$, $\beta=0.001$, and $\gamma=0.01$, respectively.

#### Assessment metrics

Multiscale structure similarity (MS-SSIM)^65^ quantifies the similarity of two images and is an improvement of SSIM^66^, which assesses the similarity between two images, $x$ and $y$, based on three factors: luminance $l\left(x,\, y\right)$, contrast $c\left(x,\, y\right)$, and structure $s\left(x,y\right)$.

13 $$l\left(x,y\right)=\frac{2{u}_{{{{{{\rm{x}}}}}}}{u}_{{{{{{\rm{y}}}}}}}+{C}_{1}}{{{u}_{{{{{{\rm{x}}}}}}}}^{2}{{u}_{{{{{{\rm{y}}}}}}}}^{2}+{C}_{1}}$$

14 $$c\left(x,y\right)=\frac{2{\sigma }_{{{{{{\rm{x}}}}}}}{\sigma }_{{{{{{\rm{y}}}}}}}+{C}_{2}}{{{\sigma }_{{{{{{\rm{x}}}}}}}}^{2}{{\sigma }_{{{{{{\rm{y}}}}}}}}^{2}+{C}_{2}}$$

15 $$s\left(x,y\right)=\frac{2{\sigma }_{{{{{{\rm{xy}}}}}}}+{C}_{3}}{{\sigma }_{{{{{{\rm{x}}}}}}}{\sigma }_{{{{{{\rm{y}}}}}}}+{C}_{3}}$$

where ${u}_{{{{{{\rm{x}}}}}}}{,\, u}_{{{{{{\rm{y}}}}}}}$ represent the average of $x,\, y$; ${\sigma }_{{{{{{\rm{x}}}}}}},\, {\sigma }_{{{{{{\rm{y}}}}}}}$ represent the variance of $x,y$; ${C}_{1}$, ${C}_{2}$ and ${C}_{3}$ are small constants given by ${C}_{1}={({K}_{1}L)}^{2}$, ${C}_{2}={({K}_{2}L)}^{2}$, and ${C}_{3}={C}_{2}/2$. Here $L$ is the dynamic range of pixel values, and ${K}_{1}$ and ${K}_{2}$ are two scalar constants.

The general form of SSIM is defined as:

16 $${{{{{\rm{SSIM}}}}}}\left(x,\, y\right)={\left[l\left(x,\, y\right)\right]}^{\alpha }{\left[c\left(x,\, y\right)\right]}^{\beta }{\left[s\left(x,\, y\right)\right]}^{\gamma }$$

where $\alpha$, $\beta$, and $\gamma$ are parameters used to define the relative importance of the three components and are set to 1 in most cases^66^.

MS-SSIM is calculated by iteratively applying low-pass filters, down sampling the filtered image result by a factor M and then calculating the SSIM index of the scaled images. The overall MS-SSIM evaluation is based on combining the measurements at different scales:

17 $${{{{{\rm{MS}}}}}}-{{{{{\rm{SSIM}}}}}}\left(x,\, y\right)={\left[l\left(x,\, y\right)\right]}^{{\alpha }_{{{{{{\rm{j}}}}}}}M}.\mathop{\prod }\limits_{j=1}^{M}{\left[{c}_{{{{{{\rm{j}}}}}}}\left(x,\, y\right)\right]}^{{\beta }_{{{{{{\rm{j}}}}}}}}{\left[{s}_{{{{{{\rm{j}}}}}}}\left(x,\, y\right)\right]}^{{\gamma }_{{{{{{\rm{j}}}}}}}}$$

where ${\alpha }_{{{{{{\rm{j}}}}}}}$, ${\beta }_{{{{{{\rm{j}}}}}}}$, and ${\gamma }_{{{{{{\rm{j}}}}}}}$ are used to adjust the relative importance of different components^65^.

HAWKMAN analysis^38^ assesses the similarity of two images based on their structures rather than their intensity, making it suitable for SMLM images whose intensity is not linearly related to the labeling density. In HAWKMAN analysis, two images are first normalized and blurred by Gaussian kernels with successive sizes up to a user-specified maximum, and the blurred images are then normalized by the maximum intensity of one. Next, the images are binarised based on the local threshold to extract the feature signals. The obtained images are regarded as sharpening images. The sharpening images are further blurred and flattened, re-binarised at a higher threshold, and then skeletonized to get skeletonized images. The skeletonized images are re-blurred with a Gaussian kernel of FWHM equal to the original scale to get the structure images. Finally, the cross-correlations of the sharpening images and the structure images are calculated to yield a confidence score of the test image (here we note as HAWKMAN score). And a confidence map is produced and a local confidence score below 0.85 indicates that the structures are less trustable.

18 $${{{{{\rm{HAWKMAN}}}}}}\; {{{{{\rm{score}}}}}}=\frac{1}{2}{\min }\left(1,\frac{{{{{{{\rm{PCC}}}}}}}^{{{{{{\rm{sharp}}}}}}}}{0.85}\right)+\frac{1}{2}{\min }\left(1,\frac{{{{{{{\rm{PCC}}}}}}}^{{{{{{\rm{str}}}}}}}}{0.85}\right)$$

where PCC^sharp^ and PCC^str^ are the Pearson correlation coefficients for the sharpening and structure images.

Signal density is computed from a GT image by first conducting binarization for the image to extract the signal-containing pixels and then calculating the ratio of the number of signal-containing pixels to the total number of pixels in the image.

19 $${{{{{\rm{signal\; density}}}}}}=\frac{{{{{{{\rm{Pixel}}}}}}}_{{{{{{\rm{signal}}}}}}}}{{{{{{{\rm{Pixel}}}}}}}_{{{{{{\rm{total}}}}}}}}$$

#### Simulation image generation

For the simulation of polymer lines, simulated polymer chains in a 10 × 10 µm^2^ region were generated in MATLAB. The polymer density was set to 50 polymers per image to mimic a densely distributed microtubule network. The GT image was created by fitting the fluorophore positions to an image with a pixel size of 10 nm and convolved with a Gaussian kernel of a 20-nm FWHM size. For the GT image, no noise and a uniform background were used. Similar to the process of generating the GT image, the corresponding LR image was generated by fitting the fluorophore positions to images with a pixel size of 100 nm and then performing convolution with a Gaussian kernel of a 200-nm FWHM size. In addition to the background, Poisson noise and read noise were added to the LR image.

For the simulation of dot pairs and line pairs, simulated line/dot pairs with a distance randomly decided in the range of 10 nm/20 nm to 50 nm, and randomly distributed in a 10 × 10 µm^2^ region were first generated. The GT image was created by fitting the signal positions to an image with a pixel size of 10 nm and convolved with a Gaussian kernel of a 20-nm FWHM size. The GT images were then blurred by a Gaussian kernel of a 280-nm FWHM size and followed by applying Poisson noise and Gaussian noise to get the LR images.

### Sample preparation

#### Cell culture and transfection

The Beas2B cell line was bought from ATCC (CRL-9609) and was grown in Dulbecco’s Modified Eagle Medium (DMEM) (Gibco) supplemented with 10% fetal bovine serum (Gibco) and 1% penicillin/streptomycin at 37 °C. The plasmid constructs used in this study included EGFR-mCherry, EGFR-EGFP (the cDNA encoding human EGFR were ordered from BGI (Beijing, China). The plasmids Str-KDEL_SBP-mCherry-EGFR and Str-KDEL_SBP-EGFP-EGFR were generated by standard molecular cloning procedures. The N-terminus of SBP-EGFP tag, SBP-mCherry tag are followed by a signal sequence derived from IL-2^67^), Tomm20-EGFP (artificially constructed based on EGFP-N1 backbone), 3XmEmerald-ensconsin (a gift from Prof. Dong Li (University of Chinese Academy of Sciences), Tomm20-mCherry (artificially constructed based on mCherry-N1 backbone), EGFP-Sec61β and Halo-clathrin (gifts from Prof. Yuhui Zhang (Huazhong University of Science and Technology)). The day before transfection, cells were seeded into the wells of a 24-well plate with 500 μL culture medium. The indicated plasmid was transfected into cells by the Lipofectamine LTX (Invitrogen) according to the standard protocol. The cells were digested with 0.25% trypsin (Thermo Fisher Scientific) 6–8 h after transfection, seeded onto confocal dishes, and cultured at 37 °C with 5% CO_2_ for another 24 h.

#### Staining organelles in fixed cells

For labeling microtubules in fixed cells, Beas2B cells cultured on coverslips after 24 h were stained according to the approach in^68^. Briefly, cells were first washed with cytoskeleton buffer (CB buffer: 10 mM MES of pH 6.1, 150 mM NaCl, 5 mM EGTA, 5 mM D-glucose, and 5 mM MgCl2) three times, prefixed with 0.6% paraformaldehyde with 0.1% glutaraldehyde and 0.25% Triton in CB buffer for 1 min. Then, cells were fixed with 4% paraformaldehyde and 0.2% glutaraldehyde in CB buffer for 15 min. After washing three times with 1× PBS, cells were incubated for 10 min in 0.1% NaBH4 to reduce background fluorescence due to glutaraldehyde, and another washing step with PBS was performed. To quench reactive cross-linkers, cells were incubated in 10 mM Tris for 10 min, followed by 2 washes with PBS. Then, cells were permeabilized in 5% BSA and 0.05% Triton X-100, diluted in PBS for 15 min, and then incubated with 1:500 mouse anti-*α*-tubulin antibody (Sigma, T6199) for 1 h, followed by three washes with PBS. Cells were then incubated with 1:500 Alexa Fluor 647 goat anti-mouse IgG (Invitrogen, A-21236) for 1 h. Finally, the cells were washed with PBS three times.

For labeling EGFR and CCP in fixed cells, Beas2B cells were cultured on coverslips after 24 h and treated with 5 ng/ml EGF in the culture medium for 3 min. Then, cells were incubated with 0.25% Triton, and 0.1% Glutaraldehyde in PEM buffer (80 mm PIPES, 5 mm EGTA, 2 mm MgCl2, pH 6.8) for 30 s. Next, cells were fixed with 0.25% Triton, and 0.5% GA in PEM for 10 min. After washing three times with 1× PBS, cells were incubated for 7 min with 0.1% NaBH4. After another washing step, cells were incubated with blocking buffer (5% normal goat serum, 0.05% Triton X-100 in PBS) for 1 h which increased to 3 h for labeling clathrin^69^. Then cells were incubated overnight with primary antibodies (1:200 Anti-EGFR antibody (R-1) (SCBT, sc-101) for EGFR and 1:200 anti-clathrin heavy chain antibody (Abcam, ab2731) for clathrin) in blocking buffer. After incubation with primary antibodies, the coverslips were rinsed using the blocking buffer (3 × 10 min). Then, cells were incubated with 1:500 corresponding secondary antibodies in the blocking buffer for 1 h.

For labeling ER and mitochondria in fixed cells, Beas2B cells were transfected with EGFP-Sec61β, Tomm20-EGFP. After being transfected for 24 h, cells were first fixed with 3% paraformaldehyde and 0.1% glutaraldehyde in PBS for 10 min, then incubated with 0.1% NaBH4 for 7 min. After a washing step with PBS, cells were blocked with blocking buffer (5% normal goat serum, 0.05% Triton X-100 in PBS) for 1 h. Then cells were incubated with 1:500 anti-GFP primary antibody (Proteintech, 50430-2-AP) in the blocking buffer for 1 h and then incubated with the secondary antibody in the blocking buffer for another hour.

For labeling nuclear pore complex in fixed cells, Beas2B cells were fixed with 4% paraformaldehyde in PBS for 10 min, then incubated with 0.2% Triton X-100 for 10 min, next blocked with blocking buffer (2.5% BSA and 0.1% Triton X-100 in PBS) for 15 min. After that cells were incubated with 1:100 anti-Nup133 antibody (Sigma-Aldrich, HPA059767) in blocking buffer at 4 °C for 12 h, and then washed four times for 30 min with PBS. Next, cells were incubated with 1:500 goat anti-rabbit Alexa Fluor 647 (Sigma-Aldrich, SAB4600184) in the blocking buffer for 2–3 h. Finally, cells were anchored with MA-NHS (Sigma-Aldrich, 730300) for 1 h. Then a gelation solution of monomers was cast across the sample and polymerized at 37 °C for 2 h. The gelation solution was prepared according to the previous method^70^. Next, cells were homogenized by proteinase K (New England Biolabs, #P8107) at 50 °C for 2 h. After homogenization, the gel was expanded with ddH2O.

For single-molecule imaging, we used the standard photoswitching buffer that contained 50 mM Tris of pH 7.5, 10 mM NaCl, 0.5 mg/mL glucose oxidase, 40 μg/mL catalase, 10% (w/v) glucose, and 1% (v/v) β-mercaptoethanol.

#### Labeling organelles in live cells

For labeling microtubules and EGF in live cells, Beas2B cells were transfected with 3XmEmerald-ensconsin plasmid. After 24 h post-transfection, cells were incubated with Qdot 655 (ThermoFisher, Q10123MP) conjugated EGF (5 ng/ml) in the culture medium for 30 min at 37 °C with 5% CO2. Then, the EGF solution is replaced by the culture medium for the following live-cell imaging.

For labeling microtubules and EGFR in live cells, Beas2B cells were co-transfected with plasmids encoding 3XmEmerald-ensconsin and EGFR-mCherry. After 24 h post-transfection, cells were prepared for live-cell imaging.

For labeling clathrin and EGFR in live cells, Beas2B cells were co-transfected with plasmids encoding Halo-clathrin and EGFR-EGFP and cultured for 24 h. Then cells were incubated with Halo-SiR in the culture medium at 37 °C for 1 h. After washing three times with the prewarmed culture medium, cells were incubated with EGF (5 ng/ml) in the culture medium for 3 min at 37 °C with 5% CO2 to induce endocytosis. Then, the EGF solution was replaced by the culture medium for the following live-cell imaging.

For labeling ER and mitochondria in live cells, Beas2B cells were transfected with Tomm20-mCherry, EGFP-Sec61β plasmids, and cultured for 24 h. After 24 h post-transfection, cells were prepared for live-cell imaging.

### Experimental data acquisition

#### Data acquisition from fixed cells

The experimental training data for fixed cells were obtained from a home-built super-resolution localization microscope^71^ based on an inverted microscope (Nikon Ti Eclipse) equipped with a 100 × 1.49 NA TIRF objective (Nikon Apo TIRF). Excitation was provided by a 500 mW 656 nm laser (CNI, MRL-N-656.5–5500 mW), and images were acquired by EMCCD (Andor, IXon-Ultra) with a 16 μm pixel size. When performing single-molecule imaging, a 1.5× telescope was used, resulting in a 106 nm effective pixel size. For training data acquisition, a WF image of every field of view was first acquired at low illuminance, and then the laser intensity was increased to the maximum to obtain single-molecule images. For super-resolution imaging, an optimal focus system and a home-built drift-correction system were used to correct system drift^71^. The software was provided by NanoBioImaging Ltd. The frame rate was set to 30 frames per second, and 20,000 frames were acquired per super-resolution image.

#### Data acquisition from live cells

The live-cell data were acquired from different systems, and the image’s effective pixel size was adjusted to ~100 nm. Specifically, the data shown in Figs. 4, 5, 7, and the corresponding supplementary figures were acquired from a commercial Zeiss Elyra 7 microscope in HILO mode with a 60×/1.46 oil objective. For a FOV size of 25.6 × 25.6 µm^2^, we recorded dual-color live-cell images at 100 Hz with 15 W/cm^2^ illuminance for 5000 time points (Figs. 4, 5, and 7, and the corresponding supplementary figures) except for the data in Fig. 7a which is recorded at 0.5 Hz for 200 time points and Supplementary Fig. 29 which is recorded at 1 Hz and for 250 time points. And for whole-cell imaging with a FOV size of 60 × 50 µm^2^, due to the data transmission limitation of the system, we used a 20 Hz imaging speed for 5000 time point recordings (Fig. 5a); in this process, the illumination intensity was reduced to 3 W/cm^2^. The data in Fig. 6 was acquired with a Zeiss SP8 confocal microscope at 3 W/cm^2^ illuminance with a 63×/1.4 oil objective. We recorded 300 time points at 0.4 Hz for a FOV of 51.2 × 51.2 µm^2^.

#### Image processing

The single-molecule image sequences were analyzed with the ThunderSTORM^72^ plug-in in FIJI. The super-resolution reconstructed images were obtained at 5× magnification for images of microtubules and 10× magnification for vesicle images. To generate the training data, the LR images were processed by a custom code to extract the edge map. To generate the training pairs of LR images, edge maps, and GT images, the LR images and edge maps were interpolated at a scale of 1.25× based on bicubic interpolation. The intensity of all images was normalized to the range of 0–255. Then, the images were split into small blocks of size 256 × 256 to correspond to the size of the GT images (64 × 64 for LR images and edge maps). Finally, ~1000 training pairs were used to train the network for the simulated polymer images; ~300 training pairs were used to train the network for the experimental images of microtubules; ~600 training pairs were used to train the network for the experimental vesicle images.

### Statistics and reproducibility

Except for network ensembles, all networks for different simulation/subcellular structures mentioned in this work were trained once per set of hyper-parameters and input dataset. For network inference results, using the same network parameters, repetition of the inference on the same input should always produce identical results.

Experiments on DNA origami (Fig. 2c) were repeated on 2 WF images of 256 × 256 pixels. Experiments for testing the effectiveness on experimental images were performed on 50 WF images of 64 × 64 pixels (Supplementary Fig. 16). Experiments for network performance evaluation on different subcellular structures in fixed cells were repeated 4 WF images of 256 × 256 pixels (Figs. 2d, e and 3a, and Supplementary Fig. 19). Experiments for testing the network robustness to different microscopies and fluorescent dyes were performed on 4 WF images of 256 × 256 pixels (Fig. 3d, e) Experiments on live-cell imaging were performed on 2 ~ 3 similar image sequences containing 2000–5000 frames (Figs. 4, 5, and 7, Supplementary Figs. 29 and 30) or 300 frames. All the simulation images were randomly generated. All the experimental images for the same experiment were acquired under the same experimental condition. No data were excluded from the analyses. Similar results were observed for the multiple incidences examined.

### Reporting summary

Further information on research design is available in the Nature Portfolio Reporting Summary linked to this article.

## Supplementary information

Supplementary Information

Reporting Summary

Description of Additional Supplementary Files

Supplementary Movie 1

Supplementary Movie 2

Supplementary Movie 3

Supplementary Movie 4

Supplementary Movie 5

Supplementary Movie 6

Supplementary Movie 7

Supplementary Software 1

## Data Availability

The source images generated in this study are publicly accessible at 10.5281/zenodo.7805563. The source data supporting the findings in this study are provided with this paper. Source data are provided with this paper.

## Source data

Source Data

## Code availability

The codes of the SFSRM network, trained models, as well as some example images for testing is publicly available at https://github.com/crrayna/SFSRM.
