# Supplementary material for: Single-frame deep-learning super-resolution microscopy for intracellular dynamics imaging
Source: Nat Commun. 2023 May 18;14:2854. doi: 10.1038/s41467-023-38452-2 (PMC10195829; doi:10.1038/s41467-023-38452-2)
Supplement: Supplementary file 1 — Supplementary Information [file 41467_2023_38452_MOESM1_ESM.pdf]

Supplementary Information for

Single-Frame Deep-Learning Super-Resolution Microscopy for Intracellular Dynamics Imaging

Rong Chen[1], Xiao Tang[2], Yuxuan Zhao[3], Zeyu Shen[2], Meng Zhang[3], Yusheng Shen[2], Tiantian Li[2], Casper Ho Yin Chung[4], Lijuan Zhang[5], Ji Wang[4], Binbin Cui[1], Peng Fei[3], Yusong Guo[2*], Shengwang Du[1,6,7*], Shuhuai Yao[1,4*]

[1]Department of Chemical and Biological Engineering, The Hong Kong University of Science and Technology, Hong Kong, China

[2]Division of Life Science, The Hong Kong University of Science and Technology, Hong Kong, China

[3]School of Optical and Electronic Information, Huazhong University of Science and Technology, Wuhan, 430074, China

[4]Department of Mechanical and Aerospace Engineering, The Hong Kong University of Science and Technology, Hong Kong, China

[5]School of Pharmaceutical Sciences, Guizhou University, Guizhou, 550025, China

[6]Department of Physics, The Hong Kong University of Science and Technology, Hong Kong, China

[7]Present address: Department of Physics, The University of Texas at Dallas, Richardson, Texas 75080, USA

*Correspondence and requests for materials should be addressed to Y.G. (email: guoyusong@ust.hk), S.D. (email: dusw@utdallas.edu), or to S.Y. (email: meshyao@ust.hk)

Content

Supplementary Note 1: Signal-to-noise ratio (SNR) estimation and measurement

Supplementary Note 2: Evaluation of the network performance on simulation images

Supplementary Note 3: Evaluation of the network performance on mismatched data

Supplementary Figure 1. Examples showing the pixelwise loss results in over-smoothed reconstruction results.

Supplementary Figure 2. Comparison of the performance of different loss functions on simulation filaments under noise-free conditions.

Supplementary Figure 3. Comparison of reconstruction results with/without the edge-map assistance under noise conditions.

Supplementary Figure 4. Subpixel edge map of a microscopic image based on the radial symmetry of the imaged fluorophore.

Supplementary Figure 5. Validation of the network reconstruction accuracy at different SNRs on simulation data.

Supplementary Figure 6. Simulation line pairs of different SNRs, interpair distances, and signal densities.

Supplementary Figure 7. Representative reconstruction results of line pairs with 10-nm interpair distance at different SNRs and densities.

Supplementary Figure 8. Representative reconstruction results of line pairs with 20-nm interpair distance at different SNRs and densities.

Supplementary Figure 9. Representative reconstruction results of line pairs with 30-nm interpair distance at different SNRs and densities.

Supplementary Figure 10. Representative reconstruction results of line pairs with 40-nm interpair distance at different SNRs and densities.

Supplementary Figure 11. Representative reconstruction results of line pairs with 50-nm interpair distance at different SNRs and densities.

Supplementary Figure 12. Average reconstruction accuracy of line pairs with different interpair distances at different SNRs and densities.

Supplementary Figure 13. Representative reconstruction results of line pairs at different SNRs and signal densities.

Supplementary Figure 14. Comparison of the HAWKMAN analysis with the network uncertainty method.

Supplementary Figure 15. Quantitative analysis of the network reconstruction accuracy on simulated dot pairs with different interpair distances.

Supplementary Figure 16. Validation of SFSRM on experimental images of microtubules in fixed cells.

Supplementary Figure 17. Evaluation of SFSRM on experimental images of different SNRs.

Supplementary Figure 18. Comparison of SFSRM with representative methods on a public dataset.

Supplementary Figure 19. Comparison of the diameters of the clathrin-coated pits measured from the STORM and the SFSRM images.

Supplementary Figure 20. Evaluation of SFSRM reconstruction uncertainty via ensemble disagreement

Supplementary Figure 21. Analysis of reconstruction artifacts at the mitochondrial junctions in fixed cells.

Supplementary Figure 22. Analysis of reconstruction artifacts at the mitochondrial junctions in live cells.

Supplementary Figure 23. Microtubule bending dynamics and microtubule tip growth and shrink instability.

Supplementary Figure 24. System drift, noise-induced reconstruction misplacements, and microtubule transverse displacements.

Supplementary Figure 25. Examples of sinusoidal-like and non-sinusoidal trajectories of vesicles.

Supplementary Figure 26. Comparison of the tracking results via SFSRM and single particle tracking.

Supplementary Figure 27. Examples of microtubule morphology changes due to vesicle transport.

Supplementary Figure 28. Quantitative analysis of the reconstruction errors at different signal densities.

Supplementary Figure 29. SFSRM reconstruction of the clathrin-coated pits (CCPs) in live cells.

Supplementary Figure 30. SFSRM imaging of interactions between the mitochondria and endoplasmic reticulum (ER) in live cells.

Supplementary Figure 31. Test network performance on data outside the training range using simulated dot pairs.

Supplementary Figure 32. Test network performance on data outside the training range using simulated clusters.

Supplementary Figure 33. Test network performance on data outside the training range using simulated curves.

Supplementary Figure 34. Test network performance on mismatched subcellular structures.

Supplementary Note 1: Signal-to-noise ratio (SNR) estimation and measurement

Theoretical estimation of SNR for live-cell images

Here we discuss how our experimental conditions including the exposure time (t), illumination density (P), and fluorescent probes for which we mainly consider its quantum yield (Φ) and duty cycle (η), will affect the acquired image signal-to-noise ratio (SNR) in live-cell experiments. Generally, the photons emitted by fluorescent proteins or probes, after passing through the optical system, are captured by a detector (e.g., a camera). For a single fluorescent molecule, its emission photon is determined by the absorbed photons Q_{ab} and quantum yield Φ of the detector:

$$Q_{\mathrm{em}} = Q_{\mathrm{ab}} \cdot \Phi \tag{1}$$

where Q_{ab} is proportional to the amount of the photons Q_{I} arrived at the sample plane and determined by the absorbance A and the on-off duty cycle η of the fluorescent molecule.

$$Q_{\mathrm{ab}} = \eta(1 - 10^{-A})Q_{\mathrm{I}} \tag{2}$$

When A is very close to zero which can be normally satisfied since the absorbance of a molecule is very small, the equation above can be reformulated as:

$$Q_{\mathrm{ab}} = (\ln 10)\eta Q_{\mathrm{I}} A \tag{3}$$

According to the Beer-Lambert law, the absorbance A of a molecule can be described by its molar attenuation coefficient ε as:

$$A = \varepsilon c L \tag{4}$$

where c is the molar concentration of the fluorescent molecule and L is the path length. Here we consider L as the depth of focus (DOF) of the objective:

$$L = \frac{\lambda}{2NA^2} \tag{5}$$

where λ is the excitation wavelength and NA is the numerical aperture of the objective.
Hence Q_{ab} can be denoted by

$$Q_{\mathrm{ab}} = (\ln 10)\eta\varepsilon c L Q_{\mathrm{I}} \tag{6}$$

Given the illumination density P, region of interest S, exposure time t, and photon energy of the excitation laser $h\upsilon$, Q_{I} can be further denoted as

$$Q_{\mathrm{I}} = PSt/h\upsilon \tag{7}$$

Taken together, the emission photon number can be calculated as follows:

$$Q_{\mathrm{em}} = (\ln 10)PSt\eta\varepsilon c L\Phi/h\upsilon \tag{8}$$

If the collected photons, further go through the dichroic mirror and bandpass filter of the detection path with a transmission coefficient T_{D} and T_{B}, The emission photons collected by the objective with a capture efficiency $\gamma = \frac{1}{2}(1 - \sqrt{1 - (NA/n)^2})$ [1], where n is the refractive index of the immersion medium, then the detected photon number Q_{det} can be denoted by:

$$Q_{\mathrm{det}} = \gamma Q_{\mathrm{em}} T_{\mathrm{D}} T_{\mathrm{B}} \tag{9}$$

Due to the inherent shot noise which is generated by the photon variation, and readout noise, dark current noise resulting from the internal process of the camera when converting photon to current, the SNR of the generated can be denoted as:

$$\mathrm{SNR} = \frac{QE.Q_{\mathrm{det}}}{\sqrt{QE.Q_{\mathrm{det}}+N_{\mathrm{r}}+I_{\mathrm{d}}}} \qquad (10)$$

where QE is the quantum efficiency; I_{d} is the dark current noise; N_{r} is the readout noise.

Here we give an example of using mEmerald as the fluorescent probe and PCO.edge 4.2 sCMOS camera as the detector for calculating the emission photon number and the relative SNR. The parameters used are listed in Supplementary Table 1.

Supplementary Table 1. The parameters and the corresponding values used in the SNR estimation.

Parameter	Value	Definition
Φ	0.68	Quantum yield of fluorophore
η	1/3	Duty cycle of fluorophore [$T_{\mathrm{on}}/(T_{\mathrm{on}}+T_{\mathrm{off}})$]
ε	$57500\ \mathrm{cm}^{-1}\mathrm{M}^{-1}$	Fluorophore extinction coefficient
c	10 µM	Fluorophore concentration
L	0.1 µm	Depth of focus of the objective
S	$0.1\times0.1\ \mu\mathrm{m}^2$	Sample area corresponding to one pixel on the detector
$h\upsilon$	4.4×10^{-19} J	Photon energy [λ_{ex} =488 nm]
P	$3\ \mathrm{W/cm}^2$	Illumination density
T	10 ms	Exposure time
γ	0.37	Capture efficiency of the objective
NA	1.46	Numerical aperture of the objective
n	1.51	Refractive index of the immersion medium
T_{D}	0.9	Transmission coefficient of the dichroic
T_{B}	0.95	Transmission coefficient of the bandpass filter
QE	0.82	Quantum efficiency of the detector
I_{d}	0.3 e-	Dark current noise
N_{r}	0.9 e-	Readout noise

The detected photon can be calculated following equation (9):

$$Q_{\text{det}} = \frac{\ln 10 \times 3\frac{W}{\text{cm}^2} \times 10\text{ms} \times (0.1\mu\text{m} \times 0.1\mu\text{m}) \times \frac{1}{3} \times 57500\frac{\text{L}}{\text{mol.cm}} \times \frac{10\text{umol}}{\text{L}} \times 0.1\mu\text{m} \times 0.68 \times 0.37 \times 0.9 \times 0.95}{4.4 \times 10^{-19}\ \text{J}} = 9.69$$

$$\text{SNR} = \frac{0.82 \times 9.69}{\sqrt{0.82 \times 9.69 + 0.3 + 0.9}} = 2.63$$

We further estimate the photon emission of three fluorescent proteins used in our experiments listed in Supplementary Table 2.

Supplementary Table 2. Typical parameters of three probes and the estimated SNRs

	λ_{ex} (nm)	λ_{em} (nm)	Φ	ε (cm^{-1} M^{-1})	Q_{det}	SNR
mEmerald[2]	482	509	0.68	57500	9.69	2.63
mCherry[3]	587	610	0.22	72000	4.78	1.71
RFP[3]	555	584	0.41	98000	11.46	2.89

The experimental conditions can be adjusted to get a certain image quality for different experiment goals. For example, for long-term observation, we maintained a low illumination intensity of 3 W/cm^2 to reduce photobleaching while increasing the exposure time to 50 ms to meet the SNR requirement; while for ultrafast imaging, we increased the illumination intensity to 15 W/cm^2 to allow for ultrahigh temporal resolution. However the hardware limitation, for instance, the data transfer speed, as well as photodamage[4] shall also be taken into consideration to optimize the photon budget.

Experimental measurement of SNR for acquired images

In our work, we use SNR as a metric for quantifying the signal level of the image, which can be obtained as follows:

a. The input image I_{m} is blurred with a Gaussian kernel with a standard deviation of five pixels to generate I_{mbur}.

b. The signal mask S_{mask} is extracted by a manually-set threshold of 0.2; and the background mask B_{mask} is extracted by a manually-set threshold of 0.1.

c. The signal region S_{pixel} and background region B_{pixel} of the input image Im are extracted by applying the signal mask S_{mask} and background mask S_{mask} corresponding to I_{m};

d. The signal μ is obtained by subtracting the averaged signal S by the camera offset $offset$, estimated by calculating the average of the background pixels, while the noise δ is composed of the signal variance $\sqrt{S - offset}$ and noise variance N, which is N the standard deviation of the background pixels.

e. The SNR of I_{m} is computed according to the following equation:

$$\text{SNR} = \frac{\mu}{\delta} = \frac{S - offset}{\sqrt{S - offset + N^2}} \tag{11}$$

Supplementary Note 2: Evaluation of the network performance on simulation images

Section I. Evaluation of the network reconstruction resolution

Considering signal variations in density and intensity during the fluorescent imaging, we systemically evaluated the resolution and accuracy of SFSRM in a range of signal density and intensity on simulation line pairs with specified interpair distances. To quantitatively assess the resolution, we simulated HR images of line pairs with random interpair distances ranging from 10 nm to 50 nm at different signal densities. The images were blurred with a Gaussian kernel of a 280-nm FWHM size, followed by applying different levels of Poisson noise and Gaussian noise to get a series of LR images with SNRs ranging from 5 to 30 (**Supplementary Figure 6**). We then trained the SRN with the simulated HR and LR image pairs and the SEN with corresponding low-SNR and high-SNR LR image pairs. To test the achievable resolution of SFSRM, we used the trained SFSRM network to reconstruct simulation line pairs with specified interpair distances (i.e., 10 nm, 20 nm, and up to 50 nm) at different signal densities and SNRs (**Supplementary Figures 7-11**) and then assessed the reconstruction accuracy at different interpair distances, signal densities, and SNRs by calculating the percentage of line pairs with correct reconstruction interpair distance to total line pairs. The statistical results in **Supplementary Figure 12** show that (i) the reconstruction accuracy is quite low (about 0.4 – 0.5) when the input SNR is about 5, and it quickly increases as the input SNR increases from 5 to 7 and then plateaus when the input SNR further increases from 7 to 30; (ii) the reconstruction accuracy of line pairs with interpair distances of 10 nm and 20 nm (< 0.8) is lower than that of 30 nm, 40 nm, and 50 nm (about 0.9); (iii) the reconstruction accuracy of line pairs with a 30-nm, 40-nm, and 50-nm interpair distance is about 0.92 when the signal density is lower than 40%, and decreases as the signal density increases from 40% to 60%. We regarded the minimum interpair distance of the line pairs that can be resolved at an accuracy higher than 0.85 as the best achievable resolution of the network,which indicates that SFSRM can generally separate two lines that are 30 nm apart when the SNR of the LR image is above 7 and the signal density is lower than 60%.

Section II. Evaluation of the network reconstruction accuracy

To further consider other potential reconstruction errors such as missing/biased structures, we assessed the overall reconstruction accuracy of the network at different signal densities and SNRs. We tested the SFSRM network with LR images of line pairs with random interpair distances between 10 nm and 50 nm at different signal densities and SNRs, and then compared the SR images with the corresponding HR images by HAWKMAN analysis [5] which gives a HAWKMAN score indicating the overall structural cross-correlation between the SR and HR images, and a confidence map marking the low-confidence structures in red (**Supplementary Figure 13**). Generally we observed more errors when the SNR is about 5, and much less as the SNR increase to 6, and almost no obvious errors when SNR is higher than 7. For different signal densities, there are generally more reconstruction errors when the signal density is higher than 50%. We then quantitatively analyzed the reconstruction accuracy by investigating the average HAWKMAN score versus the input SNRs for different signal densities. As shown in **Supplementary Figure 14b**, the HAWKMAN score quickly increases from ~0.68 to above 0.9 when the input SNR increases from 5 to 7 and then plateaus as the input SNR further increases, similar to the trends observed in **Supplementary Figure 12**. Besides, when the input SNR is higher than 7, most HAWKMAN score is ~0.97 when the signal density is lower than 40%, and it decreases as the signal density from 40% to 60%, consistent to the reconstruction accuracy decrease caused by the signal density increase (**Supplementary Figure 12**). Generally, SFSRM can achieve an accuracy over 0.9 when the SNR of the LR image is above 7 and signal density is lower than 60%.

The HAWKMAN analysis can detect the local reconstruction errors in the SR image, however it is not applicable when applying the SFSRM method to live-cell imaging where the reference HR image in unavailable. To evaluate the network reliability without the assistance of HR images, we considered two types of uncertainties in the network reconstruction that are aleatoric uncertainty and epistemic uncertainty[6, 7]. Aleatoric uncertainty considers the uncertainty caused by the image noise, while epistemic uncertainty is the uncertainty related to the model parameters. We investigated the aleatoric uncertainty by applying the SFSRM network to 10 LR images that are degraded from the same HR images by independently applying the same level of noise and then assessing the agreement of 10 SR images; and epistemic uncertainty by the ensemble disagreement method[7] which measures the reconstruction consistency of 5 independently-trained network ensembles. The results suggest the regions that are highly different in the ensemble reconstructions could be problematic. The overall reconstruction uncertainty of each pixel is calculated by averaging the aleatoric uncertainty and the epistemic uncertainty. For better comparison with the HAWKMAN analysis, we converted the uncertainty score to the agreement score and output the agreement map. As shown in **Supplementary Figure 14**, we compared the less-confident regions identified by the HAWKMAN analysis and the reconstruction uncertainty method. We noticed that the reconstruction uncertainty method can detect some errors consistent with those detected by the HAWKMAN analysis (marked by white arrows).

However, some errors in the confidence map are not detected in the agreement map (marked by red arrows). In rare cases, the agreement map misidentifies correct structures as problematic (marked by yellow arrows). The average agreement score versus the SNR at different signal densities **Supplementary Figure 14c** shows similar trends with the HAWKMAN score, indicating that the reconstruction uncertainty analysis could serve as an alternative strategy to detect some reconstruction errors in live-cell super-resolution imaging.

Supplementary Note 3: Evaluation of the network performance on mismatched data

The success of deep learning network lies in the use of adequate training data. SFSRM achevies a satisfactory reconstruction accuracy when the test data matchs the training data. However, there are risks that structural features in the test data are outside the training dataset range, which is also known as data drift in deep learning. Here we assessed the performance of SFSRM on mismatched data in three simulation scenarios: (a) we trained the network with simulated dot pairs with distances between 40 and 60 nm and tested the network with dot pairs with distances between 20 and 100 nm; (b) we trained the network with simulated clusters with diameters between 40 and 80 nm and tested the network with clusters with diameters between 20 and 150 nm; (c) we trained the network with curves with curvatures between 150 nm and 450 nm and tested the network with curves with curvatures between 30 nm and 1500 nm.

From the results in **Supplementary Figures 31-33**, we noticed that our network can hardly recognize the data drift especially when the drift is relatively small. As shown in **Supplementary Figure 31** (w/o transfer learning), when the network is trained by dot pairs of an interpair distance between 40 nm to 60 nm while tested by dot pairs of an interpair distance between 20 nm and 100 nm, the network output range is inside the range of 40 nm – 60 nm. While for larger data drift, as shown in Supplementary Figures SN11, when the network is trained by clusters with diameters between 40 nm and 80 nm and tested by clusters with diameters between 20 nm to 160 nm, we notice that when the diameter is smaller than 140 nm, the network can not detect the input data drift and will output clusters with diameter in 40 nm to 80 nm range; however, when the diameter of the cluster further increases from 140 nm to 160 nm, the network can recognize the input data drift while abnormal results are produced, which also appears as higher standard variations in the reconstructed diameter distribution of the clusters (**Supplementary Figure 32**, w/o transfer learning). And for even larger data drift as shown **Supplementary Figure 33**, when the network is trained by curves with curvature between 150 nm and 450 nm and tested with curvature between 30 nm and 1500 nm, the network can generally reconstruct curves with right curvature, but the shape is not as perfect as those inside the training range.

Based on these experimental results, we conclude that currently SFSRM can not be used to reconstruct data that is outside the training data range. For small data drift, the network may produce biased results, and for large data drift, the network can recognize the drift but output abnormal results. A convenient way to improve the network performance on datasets outside the training data range is to fine-tune the trained network on a dataset that matches the test dataset range. **Supplementary Figures 31-33** (w/ transfer learning) show that the network can generate unbiased output only after 10 epochs of training.

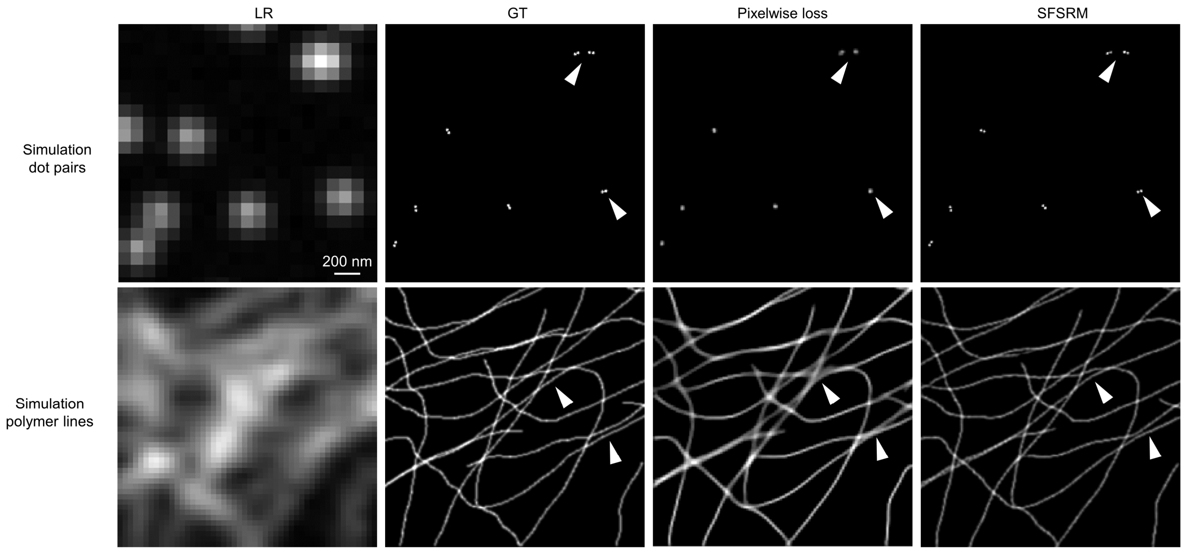

Supplementary Figure 1. **Examples showing the pixelwise loss results in over-smoothed reconstruction results.** The generator of ESRGAN is first trained by the pixelwise loss (MS-SSIM-L1 loss) and the multicomponent loss used in SFSRM separately with the same training dataset. Then the trained generators are used to reconstruct the SR images from the same LR image. The white arrows indicate where the fine structures in the GT images are blurred by the network trained with pixelwise loss while correctly reconstructed by the network trained with multicomponent loss.

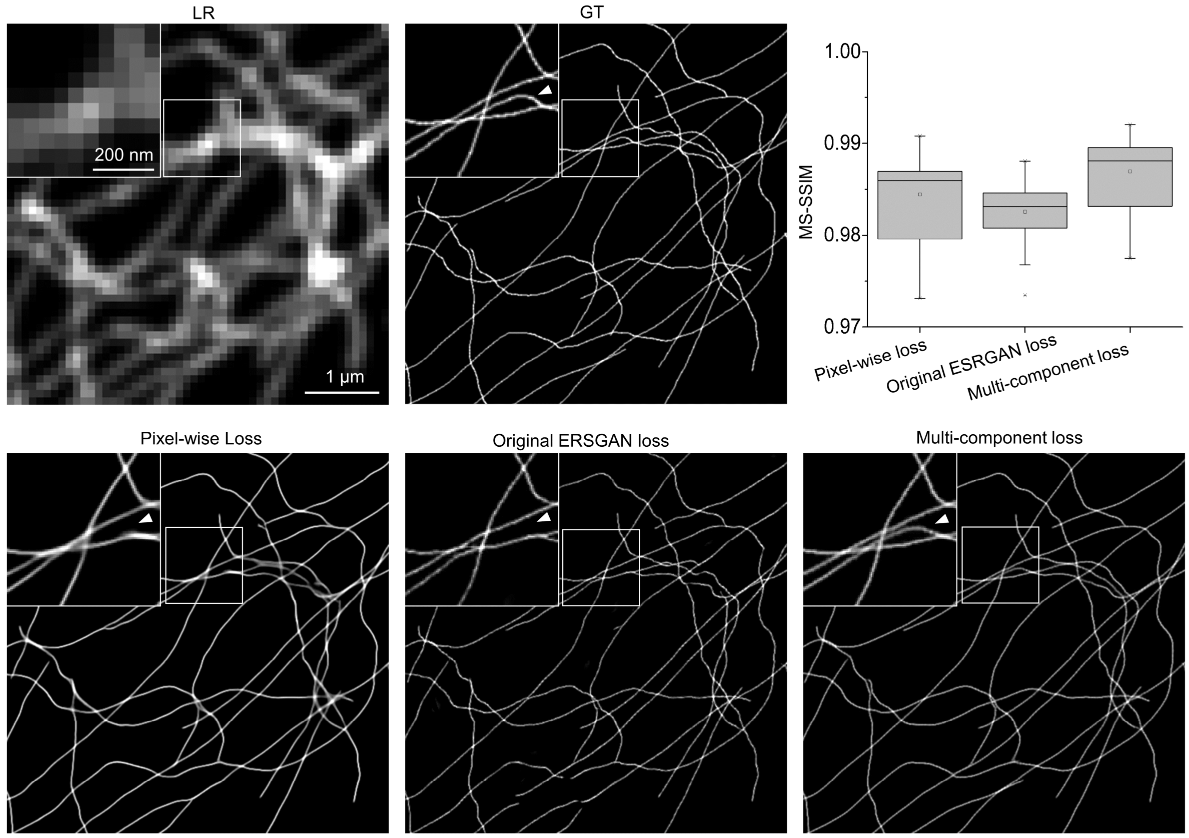

Supplementary Figure 2. Comparison of the performance of different loss functions on simulation filaments under noise-free conditions. The generator of ESRGAN is first trained by the pixelwise loss (MS-SSIM-L1 loss), the original loss used in ESRGAN, and the multicomponent loss used in SFSRM separately with the same training dataset. Then the trained generators are used to reconstruct the SR image from the same LR image. The white arrows indicate where the fine structures in the GT images are blurred by the network trained with pixelwise loss, mis-reconstructed by the network trained with the original ESRGAN loss, while correctly reconstructed by the network trained with multicomponent loss. The network reconstruction fidelity is measured by multi-scale structure similarity (MS-SSIM) between the reconstructed images and the GT image. The error bar represents reconstruction experiments repeated on 30 images. Boxplots are drawn from the 25th to 75th percentile with the horizontal bar at the median and the whiskers extending to the minima and maxima.

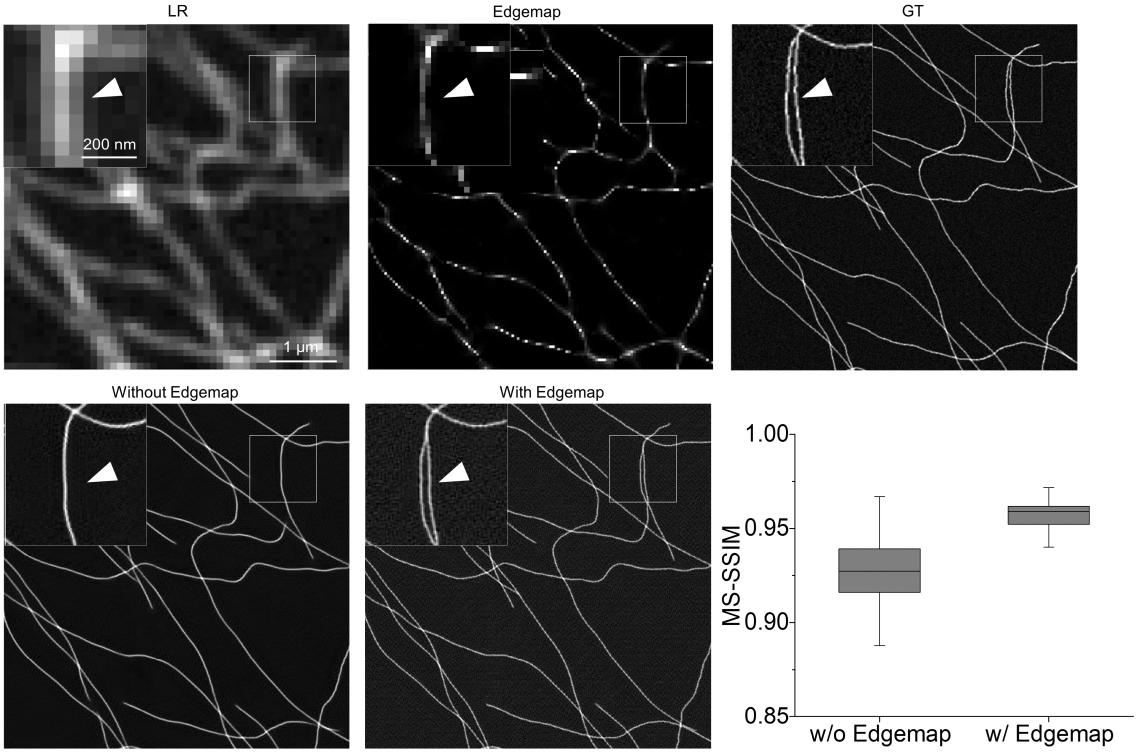

Supplementary Figure 3. Comparison of reconstruction results with/without the edge-map assistance under noise conditions. The network is first trained with single LR input, and LR together with the corresponding edge map as inputs, separately using the same training dataset. Then the trained networks are used to reconstruct the SR images from the same LR image. Compared to the network trained with single LR input, the network trained with LR as well as its edge map demonstrates higher reconstruction accuracy as shown in the zoom-in region. Quantitative analysis of the reconstruction fidelity via multi-scale structure similarity (MS-SSIM) between the reconstructed images and the GT image also shows the network with the edge map as input achieves a higher MS-SSIM index. The error bar represents reconstruction experiments repeated on 30 images. Boxplots are drawn from the 25th to 75th percentile with the horizontal bar at the median and the whiskers extending to the minima and maxima.

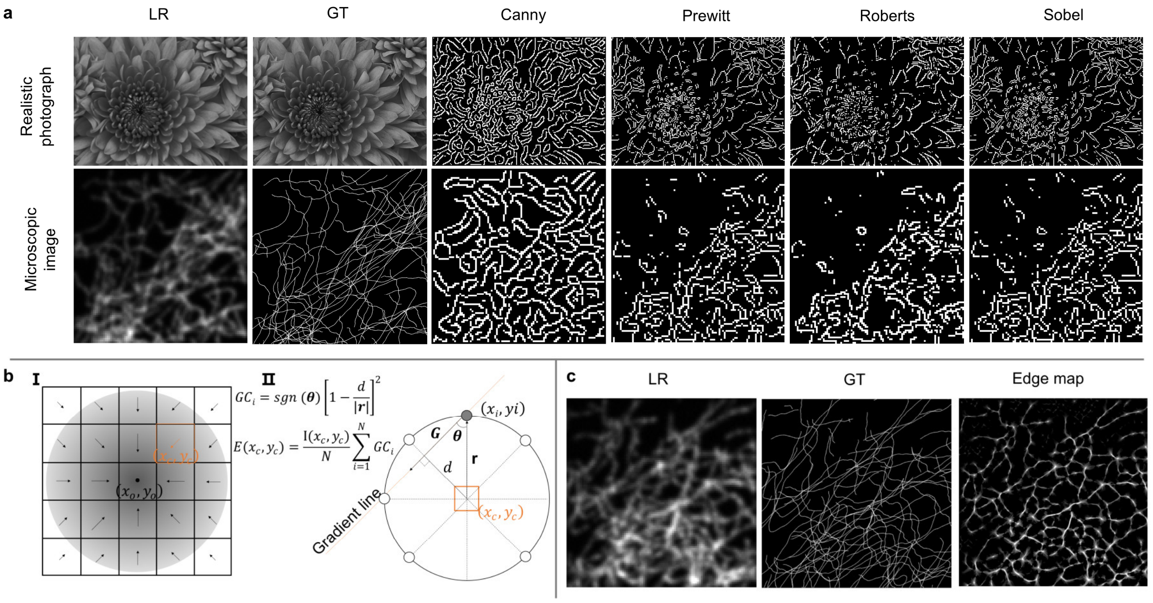

Supplementary Figure 4. **Subpixel edge map of a microscopic image based on the radial symmetry of imaged fluorophores.** **a**, Comparison of the low-resolution (LR) image, ground-truth (GT) image, and edge maps extracted by Canny, Prewitt, Roberts, and Sobel edge detection operator from the realistic photograph and microscopic images. These common edge detectors work well for the realistic photograph however cannot indicate the real edges of the microscopic GT image. **b**, Illustration of the calculation of the edge map based on the fluorophore's radial symmetry. (Ⅰ) The gradient map of a fluorophore located in (x_o, y_o). Since the gradient map is symmetrically distributed and converges to the location of the fluorophore, the local gradient convergence can be utilized to measure the fluorophore signal distribution. (Ⅱ) For an arbitrary coordinate (x_c, y_c), the edge map value $E(x_c, y_c)$ is determined by the gradient convergence (GC_i) index of N coordinates (x_i, y_i) evenly distributed surrounding it with a distance of r , where GC_i indicates the angle between the gradient vector $\boldsymbol{G}$ and radical vector $\boldsymbol{r}$. The final edge map $E(x_c, y_c)$ is further weighted by the local fluorescent signal intensity. **c**, An example of the edge map extracted from a microscopic LR image by our subpixel edge detector, which fits well with the true edges in the GT image.

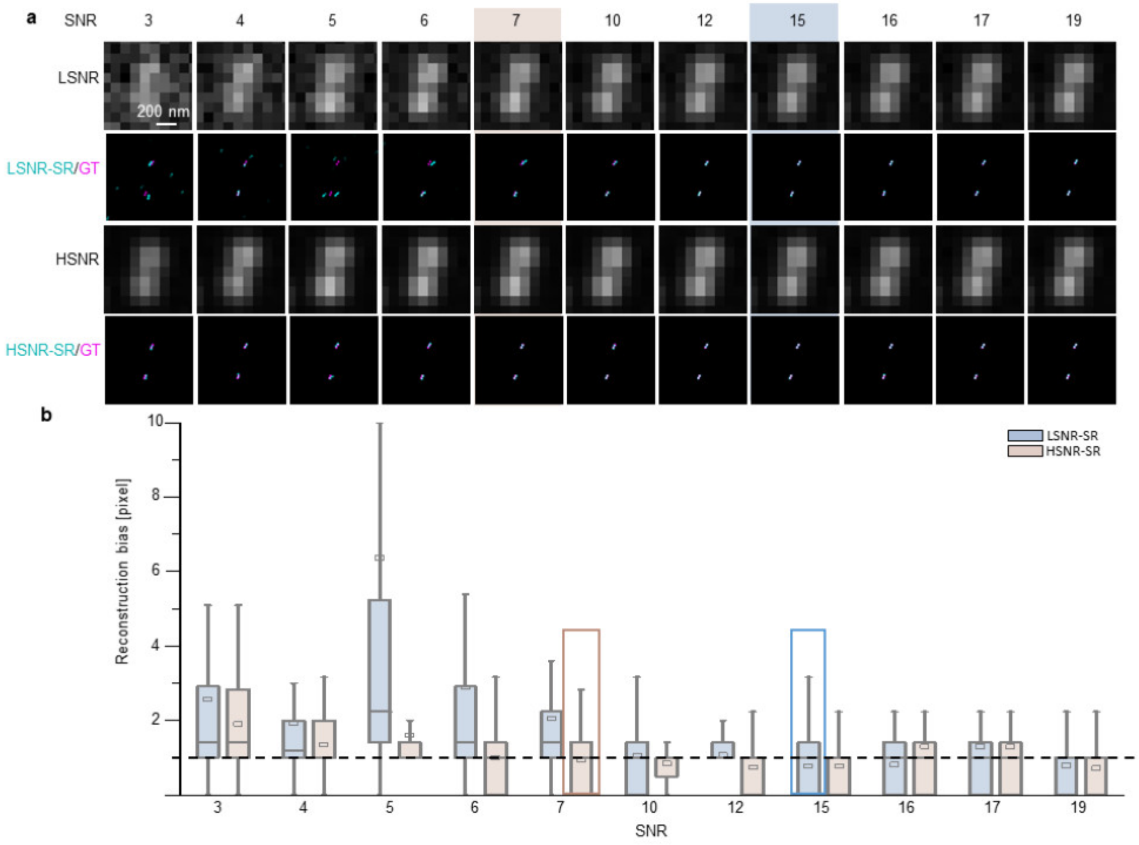

Supplementary Figure 5. **Validation of the network reconstruction accuracy at different SNRs on simulation data.** **a**, The first row shows the original low-SNR images noted as LSNR; the second row shows the dual-color merged images of the super-resolution network (SRN) reconstruction results from the original LSNR images (noted as LSNR-SR) rendered in green and the GT image rendered in red; the third row shows the intermediate results from the signal- enhancement network (SEN), noted as high-SNR (HSNR); the fourth row shows the dual-color merged images of the SRN reconstruction results from the HSNR images (noted as HSNR-SR) and the GT image. **b**, Comparison of the reconstruction bias of the SR reconstructions from inputs with (HSNR, light-red) and without (LSNR, light-blue) denoising at different SNR levels. The reconstruction bias indicates the spot location difference between the SR and GT images. If we require an average reconstruction bias within one pixel, the suggested SNR of the input image should be higher than 15 without SEN and higher than 7 with SEN. Boxplots are drawn from the 25th to 75th percentile with the horizontal bar at the median and the whiskers extending to the minima and maxima.

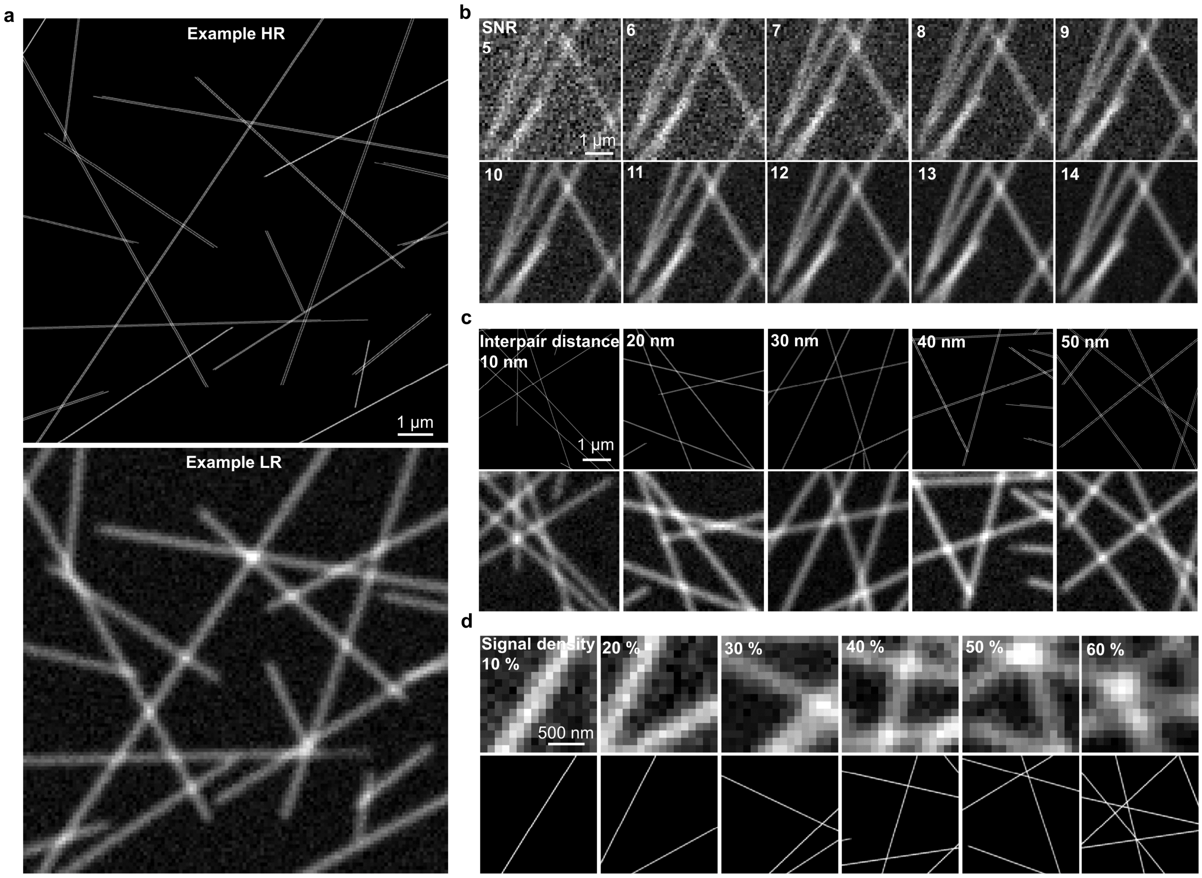

Supplementary Figure 6. Simulation line pairs of different SNRs, interpair distances, and signal densities. **a**, Example images from the training dataset showing the HR and corresponding LR images of line pairs. **b**, Examples showing the LR images of different SNRs. **c**, Examples showing the HR and LR images of line pairs with different interpair distances. **d**, Examples showing the HR and LR images of line pairs of different densities.

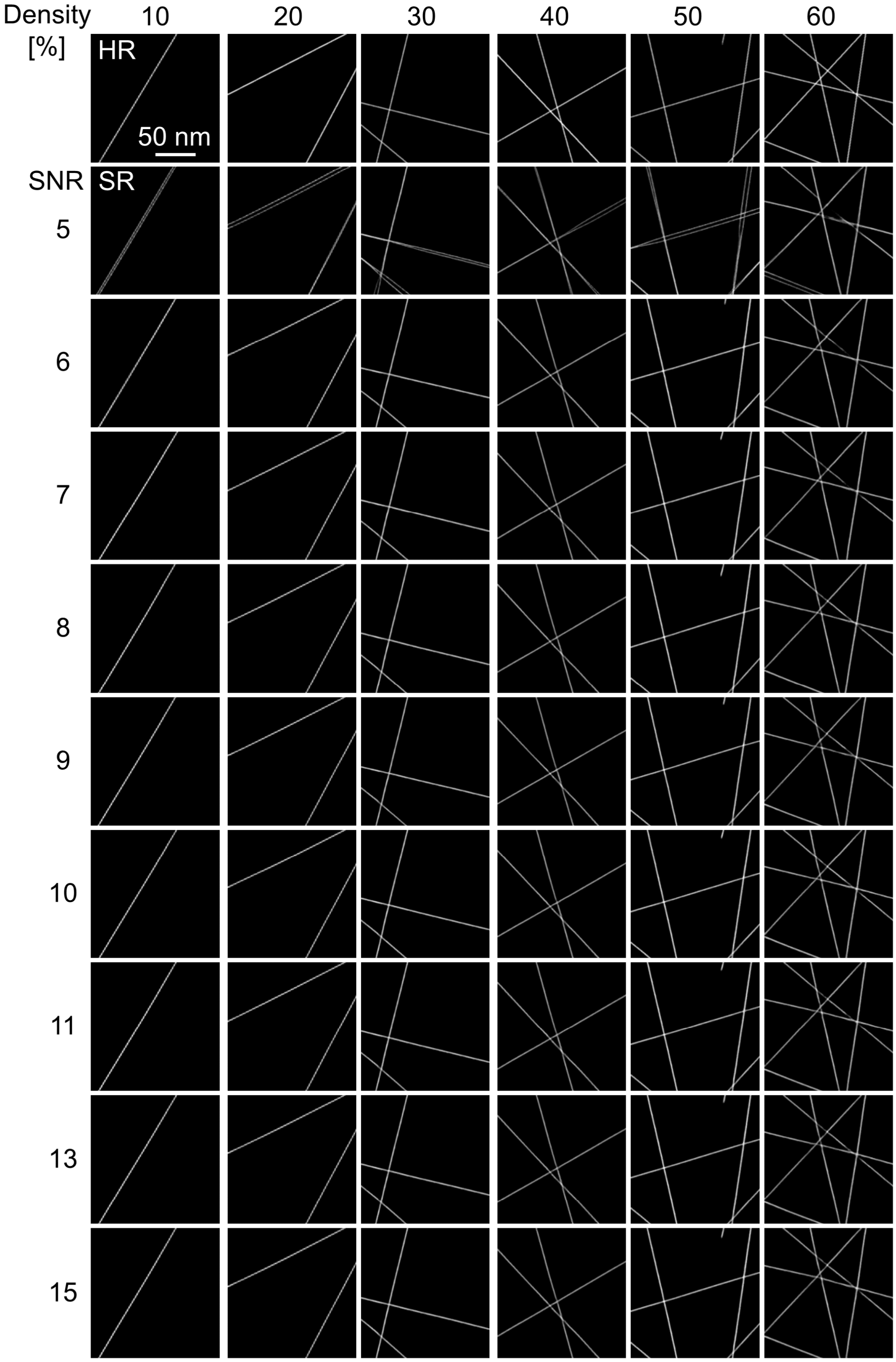

Supplementary Figure 7. Representative reconstruction results of line pairs with 10-nm interpair distance at different SNRs and densities.

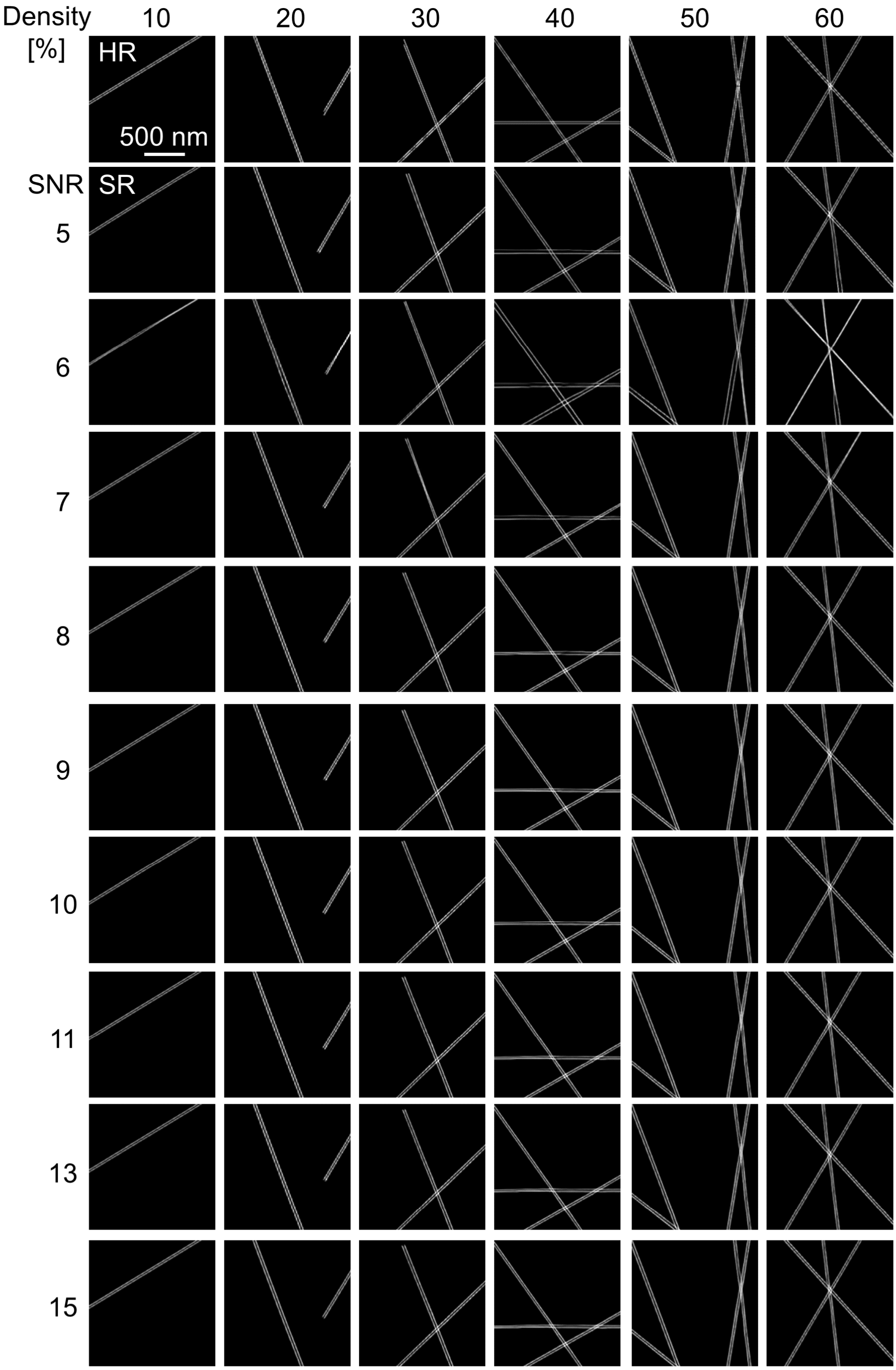

Supplementary Figure 8. Representative reconstruction results of line pairs with 20-nm interpair distance at different SNRs and densities.

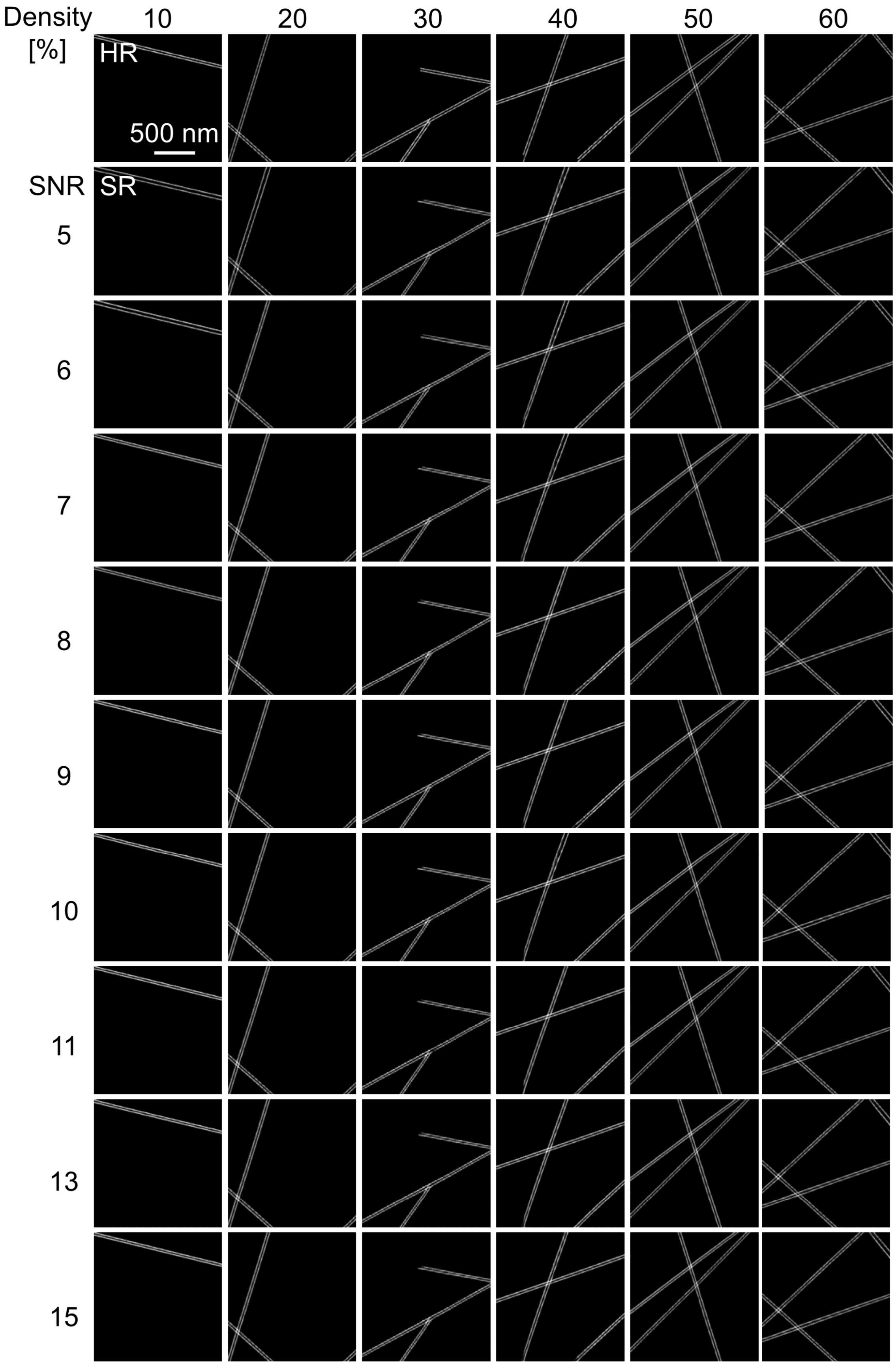

Supplementary Figure 9. Representative reconstruction results of line pairs with 30-nm interpair distance at different SNRs and densities.

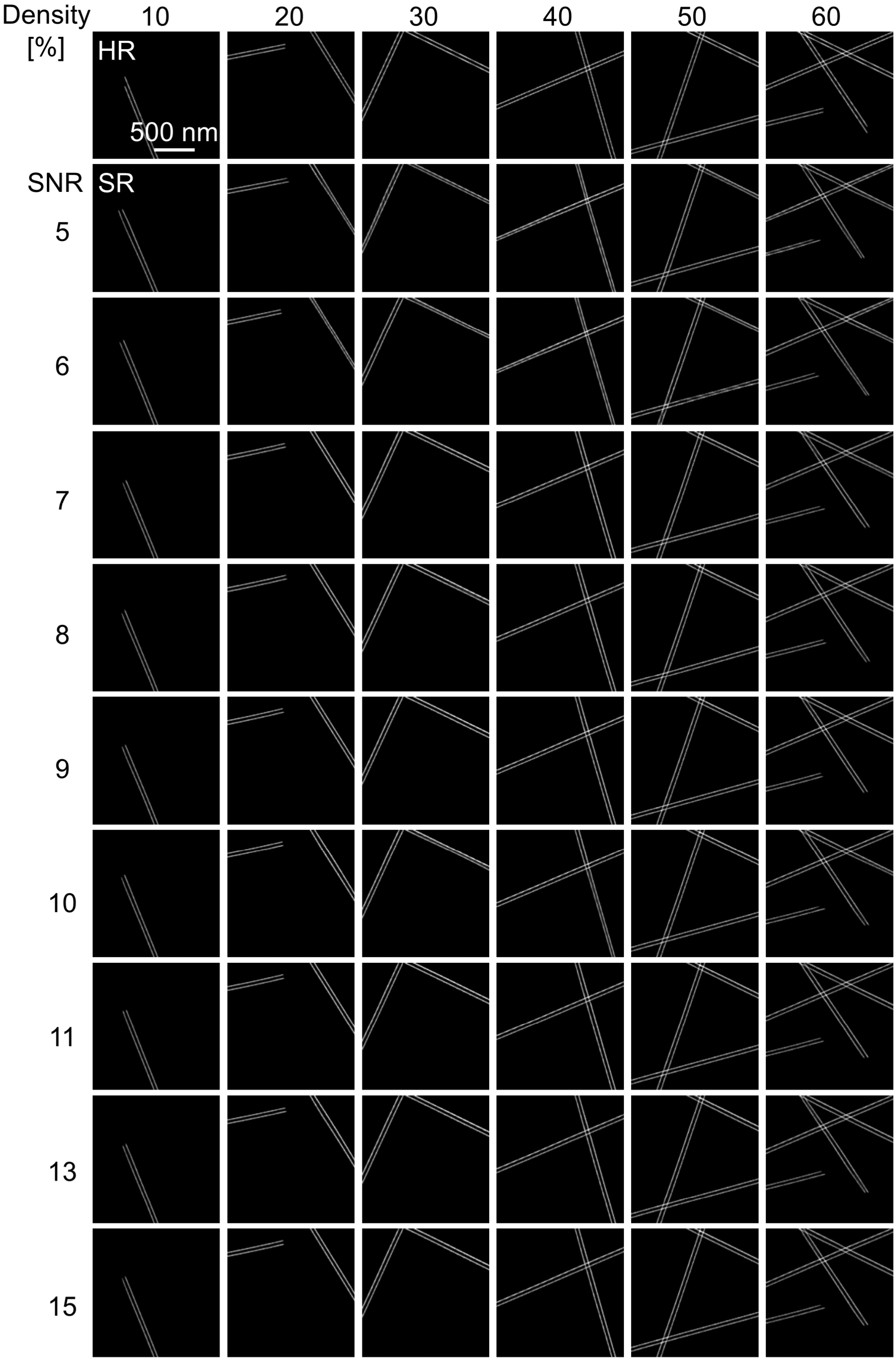

Supplementary Figure 10. Representative reconstruction results of line pairs with 40-nm interpair distance at different SNRs and densities.

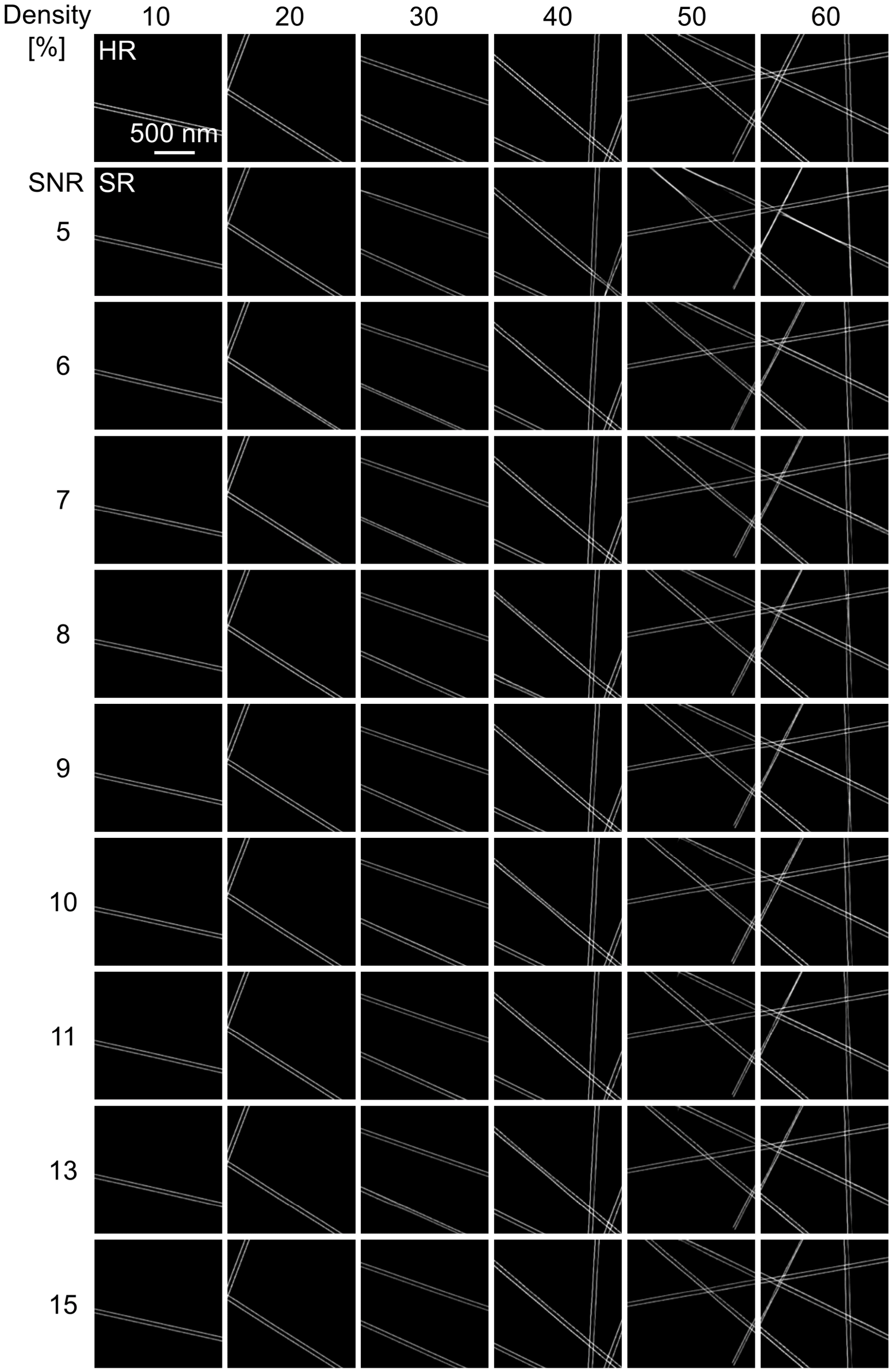

Supplementary Figure 11. **Representative reconstruction results of line pairs with 50-nm interpair distance at different SNRs and densities**.

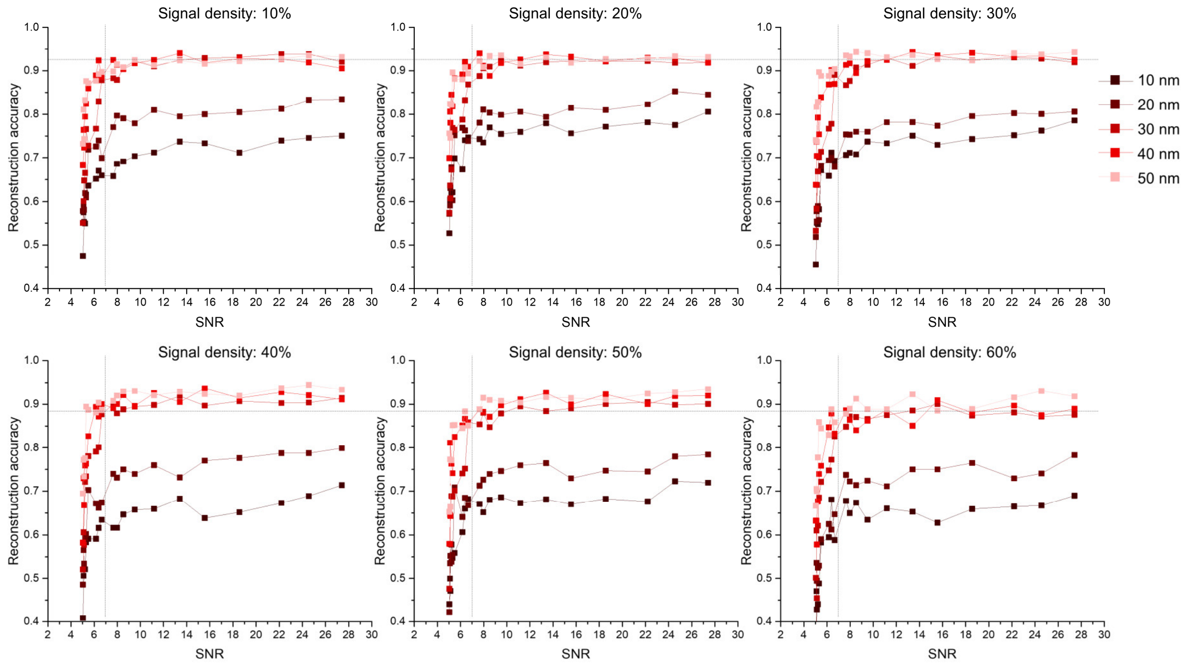

Supplementary Figure 12. Average reconstruction accuracy of line pairs with different interpair distances at different SNRs and densities.

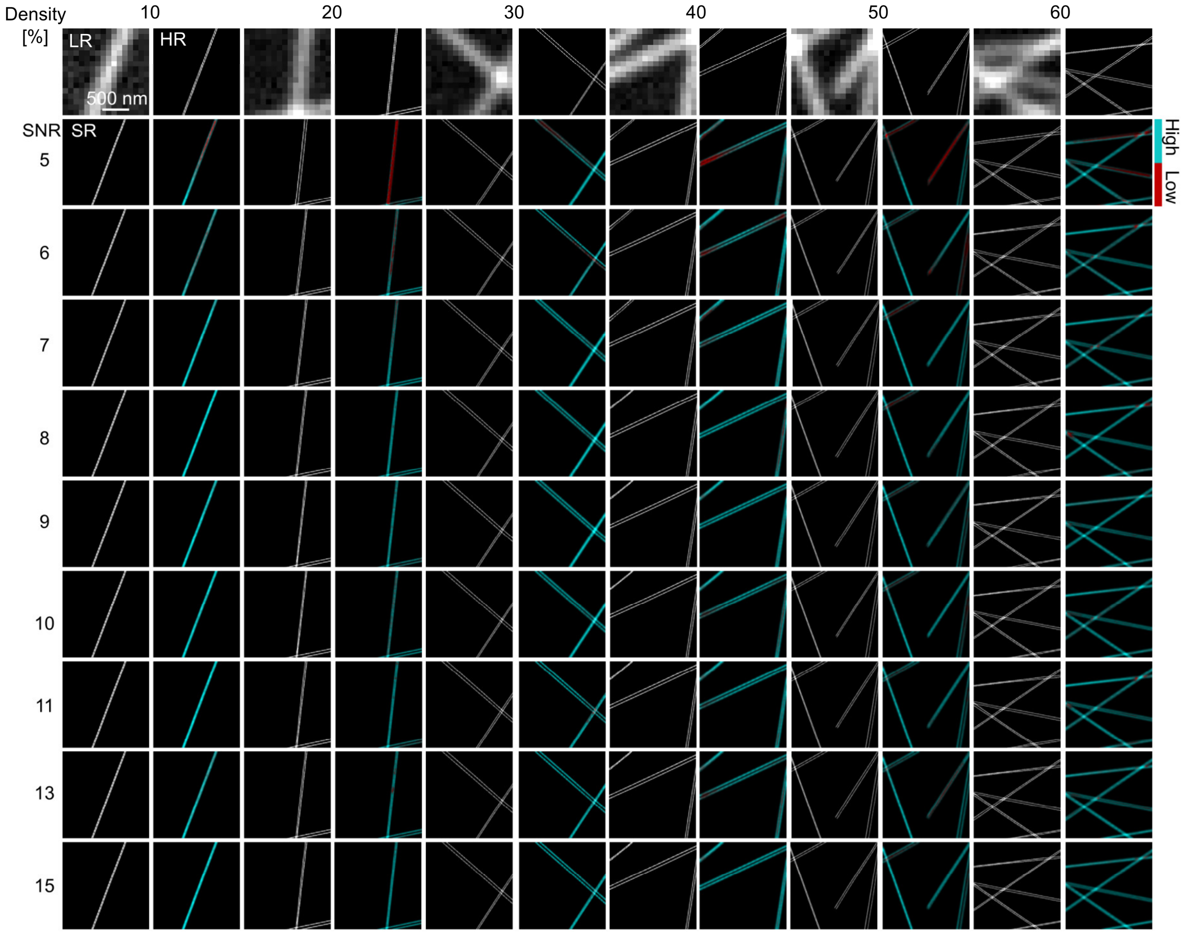

Supplementary Figure 13. Representative reconstruction results of line pairs at different SNRs and signal densities. The confidence map shows the reliable region in cyan and the less reliable region in red.

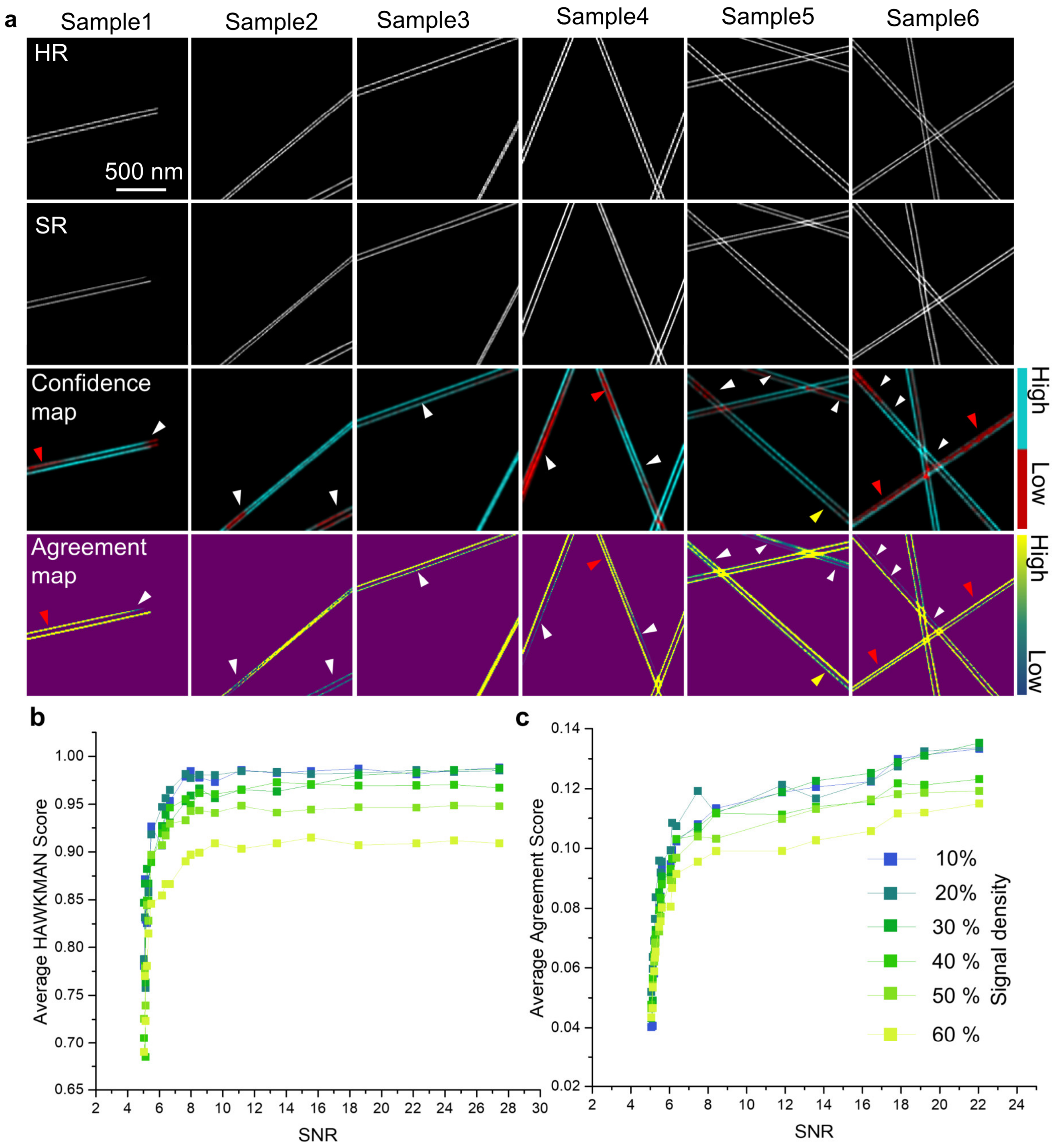

Supplementary Figure 14. Comparison of the HAWKMAN analysis with the network uncertainty method. **a**, Comparison of the confidence maps and the agreement maps. The white arrows indicate the errors that are consistent both in the confidence map and the agreement map; the red arrows indicate the error undetected in the agreement map; and the yellow arrows indicate the artificial errors detected by the agreement map. **b**, Average HAWKMAN scores of SFSRM reconstruction results at different SNRs and signal densities. **c**, Average agreement scores of SFSRM reconstruction results at different SNRs and signal densities.

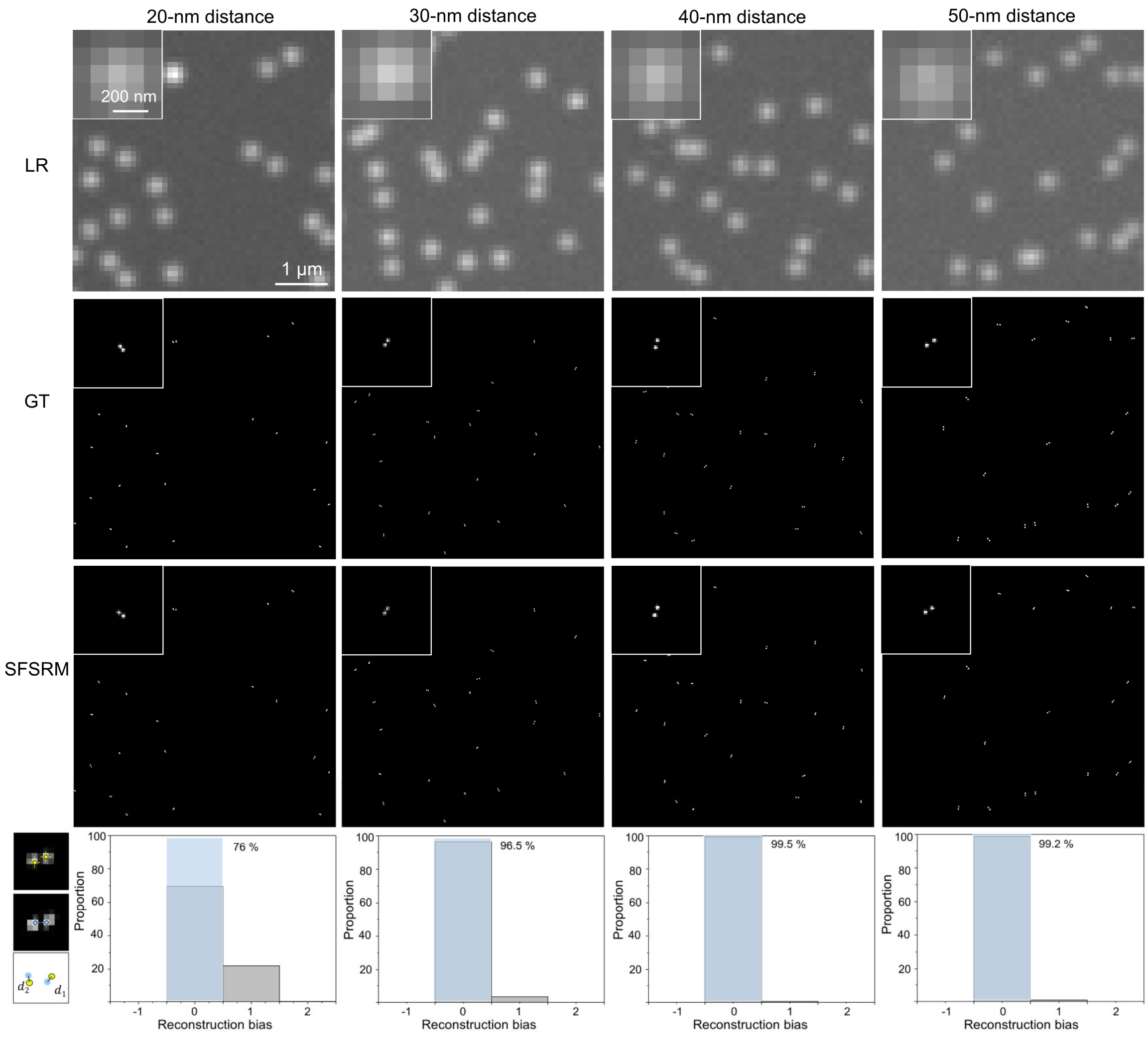

Supplementary Figure 15. **Quantitative analysis of the network reconstruction accuracy on simulated dot pairs with different interpair distances.** The network is first trained by the simulated images with dot pairs of random interpair distances ranging from 20 nm to 50 nm. Then the trained network is used to reconstruct the SR images from the LR images with dot-pairs of uniform interpair distance set as 20 nm, 30 nm, 40 nm, and 50 nm respectively. The reconstruction accuracy is quantified by the reconstruction bias calculated as follows: first, the maxima of the spots both in the GT and SR images are determined as the location of the spots; then the distance between the locations of the same spot in the GT and SR image (i.e. d_1,d_2) is calculated and normalized to half of the interpair distance as the reconstruction bias. The histograms indicate the distribution of the reconstruction bias of the dot pairs with different interpair distances.

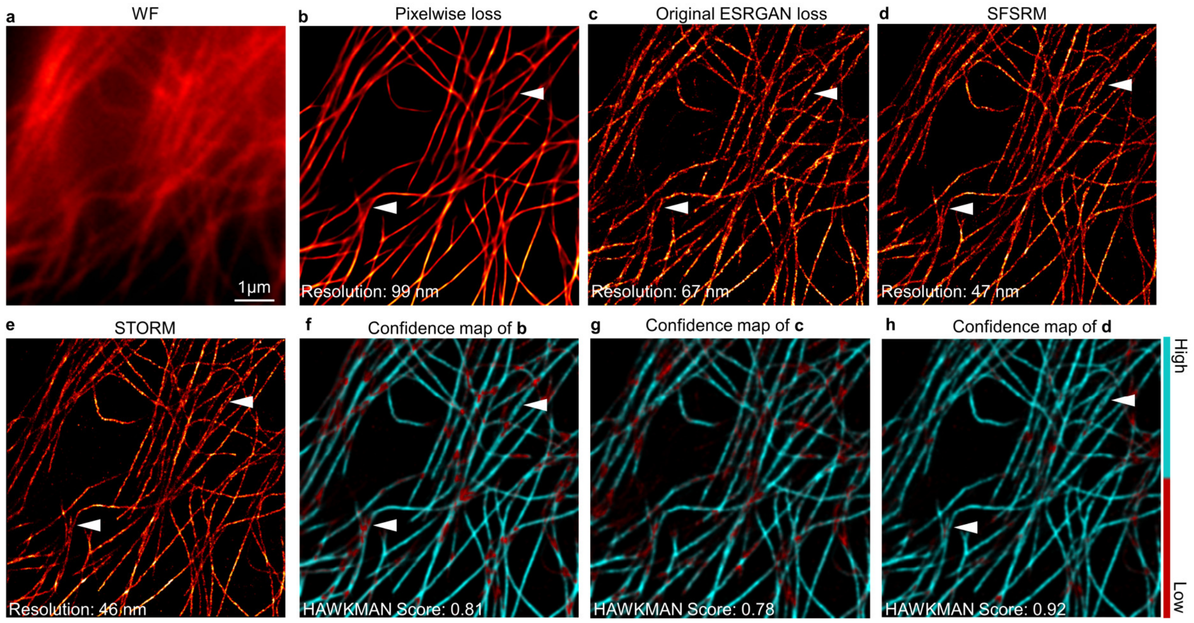

Supplementary Figure 16. **Validation of SFSRM on experimental images of microtubules in fixed cells. a**, The WF image of microtubules in Beas2B cells immunostained by Alexa Fluor 647. **b**, Reconstruction result of the ESRGAN generator trained by MS-SSIM-L1 loss. **c,** Reconstruction results of the ESRGAN generator trained by its original loss. **d**, Reconstruction result of the ESRGAN generator trained with multicomponent loss and using both the WF and the edge map as inputs. **e**, STORM reconstruction from 20,000 frames of single-molecule images, regarded as the GT image. **f-h**, The corresponding confidence maps of the reconstruction images **b-d** calculated by HAWKMAN analysis[5] in which the high-confidence structures are rendered in cyan and the low-confidence structures are rendered in red. The HAWKMAN scores indicate the overall cross-correlation of the reconstruction images with the STORM image. The reconstruction resolution is measured by image decorrelation analysis[8].

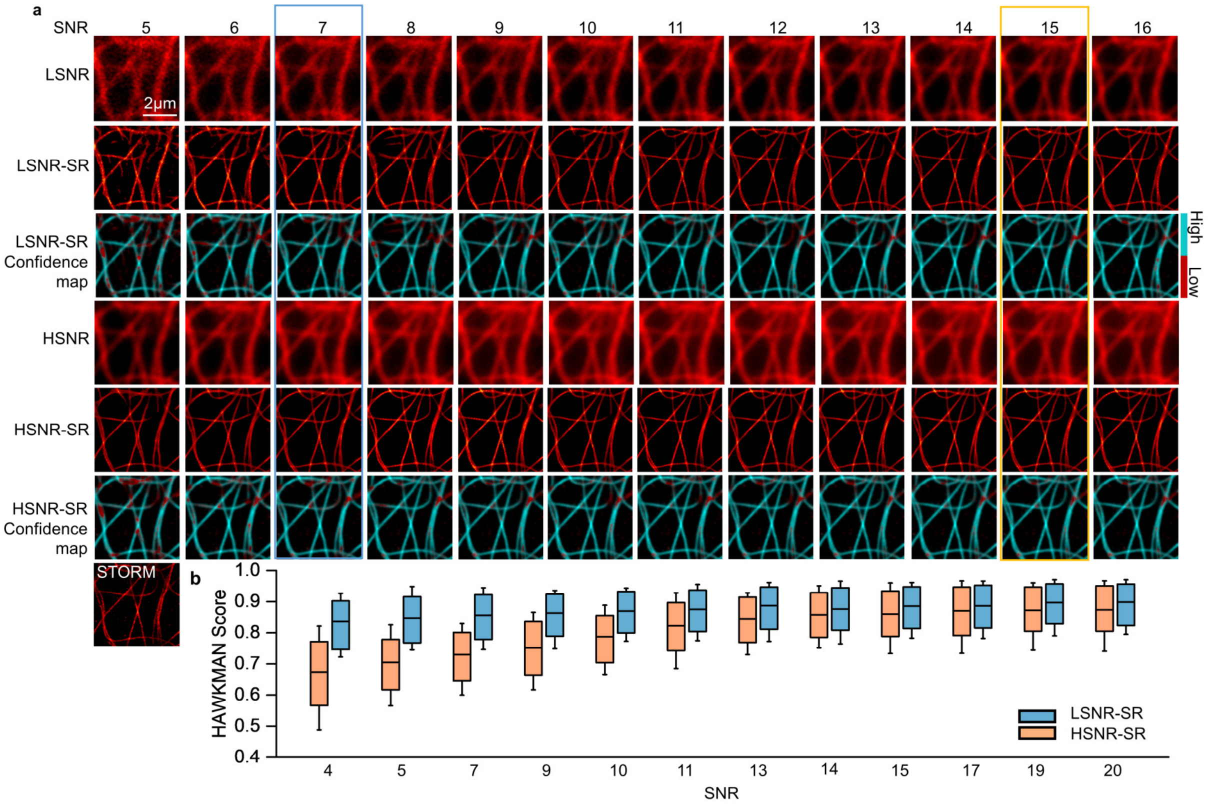

Supplementary Figure 17. Evaluation of SFSRM on experimental images of different SNRs. a, The first row shows the original low-SNR images noted as LSNR; the second row shows the super-resolution network (SRN) reconstruction results from the original LSNR images, noted as LSNR-SR; the third row shows the confidence maps of the LSNR-SR images in which the high-confidence structures are rendered in cyan and the low-confidence structures are rendered in red; the fourth row shows the intermediate results from the signal-enhancement network (SEN), noted as high-SNR (HSNR); the fifth row shows the SRN reconstruction results from the HSNR images, noted as HSNR-SR; the sixth row shows the confidence maps of the HSNR-SR images. The last row shows the STORM image reconstructed from 20,000 frames of single-molecule images, regarded as the GT images. **b**, The HAWKMAN scores of LSNR-SR and HSNR-SR images at different SNRs. The error bar represents measurements from 15 regions. Generally, the HSNR-SR images have higher HAWKMAN scores than the corresponding LSNR-SR images especially when the SNR is lower than 15. However, when SNR is extremely low (SNR < 7), even though the HSNR-SR image could have a higher HAWKMAN score than the LSNR-SR image, its accuracy may not be enough for some quantitative analysis. Hence we suggest the SNR of the input image should be higher than 15 without SEN and higher than 7 with SEN to ensure a reliable reconstruction result (HAWKMAN score higher than 0.8). Boxplots are drawn from the 25th to 75th percentile with the horizontal bar at the median and the whiskers extending to the minima and maxima.

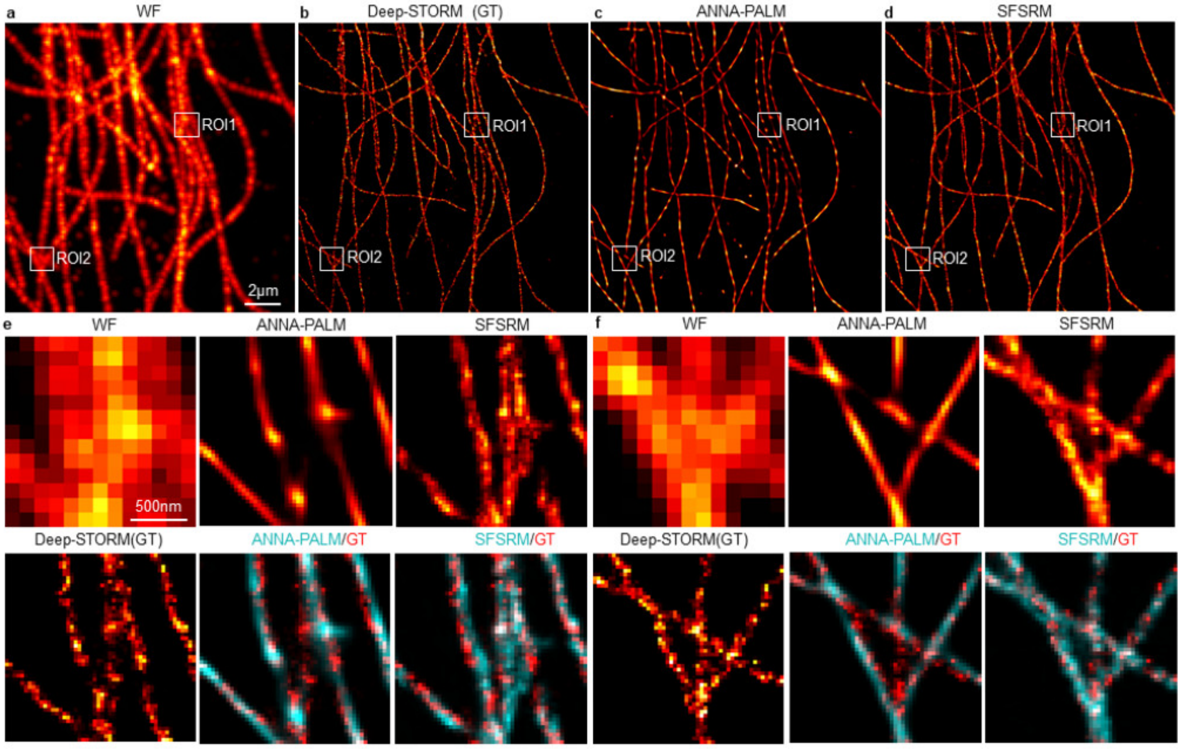

Supplementary Figure 18. Comparison of SFSRM with representative methods on a public dataset. **a**, Experimental WF image of microtubules generated by projecting the acquisition stack. **b**, Reconstruction by Deep-STORM from 300 frames of densely-distributed single-molecule images regarded as the GT image. **c**, Reconstruction by ANNA-PALM from the WF image. **d**, Reconstruction by SFSRM from the WF image. **e-f**, Zoom-in views of ROI1 and ROI2 in **a-d** and the merged images of the reconstructions **c-d (**in cyan) with Deep-STORM (GT) image **b** (in red).

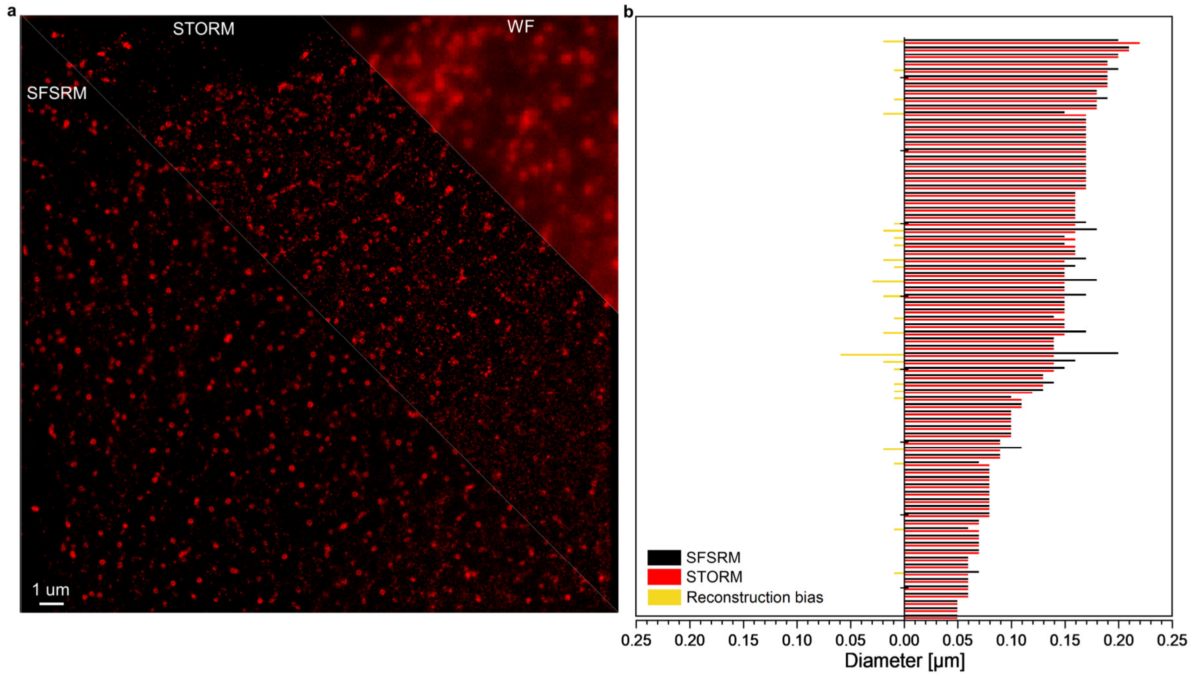

Supplementary Figure 19. Comparison of the diameters of the clathrin-coated pits measured from the STORM and SFSRM images. **a**, Representative WF, STORM, and SFSRM images of the clathrin-coated pits (CCPs) in Beas2B cells. **b**, Relative diameters of the CCPs in the STORM and SFSRM images, and the corresponding reconstruction errors.

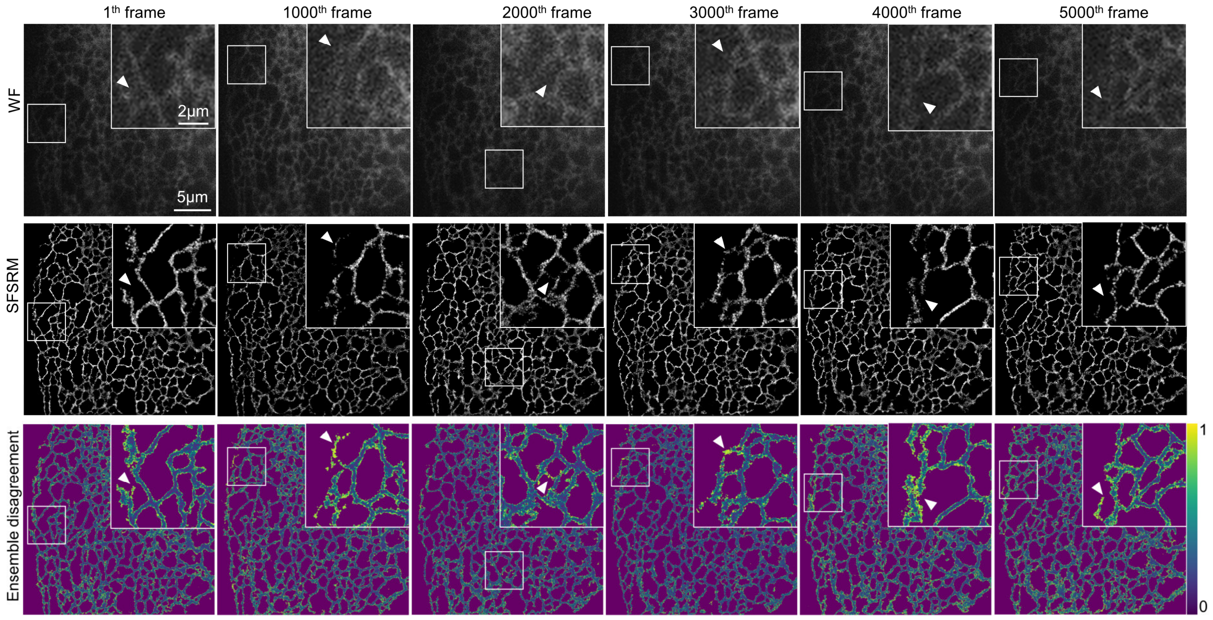

Supplementary Figure 20. Evaluation of SFSRM reconstruction uncertainty via ensemble disagreement. Top row: Time-lapse images of endoplasmic reticulum in live cells imaged with 15 W/cm^2 intensity illumination for 5000 frames. Middle row: the corresponding SFSRM reconstructions from the WF images. Bottom row: ensemble disagreement of six networks independently trained with the same dataset. The regions with high disagreement scores (highlighted by white arrows) indicate the reconstruction results of network ensembles are highly different in these regions thereby they may be less trustworthy.

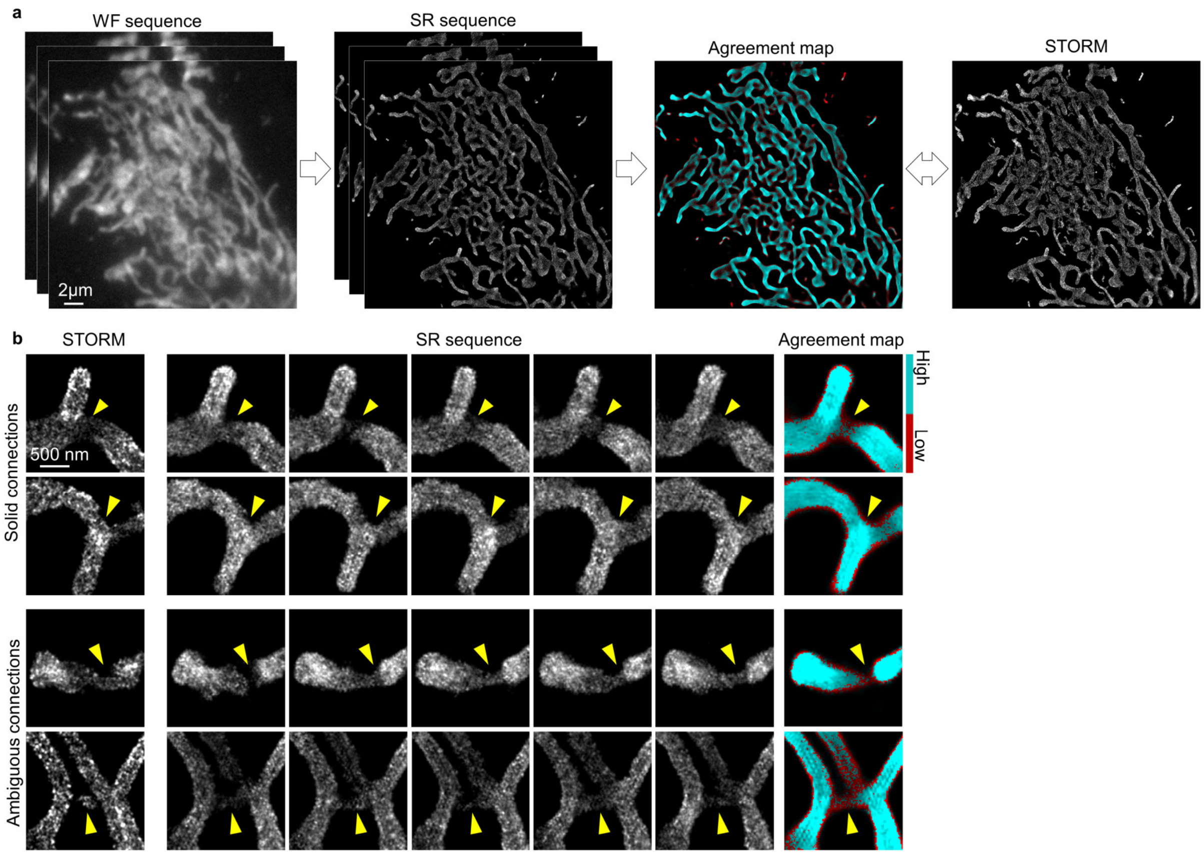

Supplementary Figure 21. Analysis of reconstruction artifacts at the mitochondrial junctions in fixed cells. **a**, The flowchart of agreement analysis in fixed cells. 100 frames of widefield images of mitochondria in fixed cells are used to reconstruct the corresponding SR images. The 100 frames of SR images are then used to calculate pixelwise agreement score. The structures with an agreement score higher than 0.85 are labeled in cyan and otherwise in red to generate the agreement map. The agreement map is then compared with the STORM image to check whether the agreement map can detect reconstruction artifacts at the mitochondrial junctions. **b**, Examples showing two kinds of mitochondrial junctions. The first one is junctions with solid connections. There are fewer reconstruction errors at such junctions and the agreement scores at the junction are high. The second one is junctions with ambiguous connections. There are 10% - 35% reconstruction errors at such junctions and the agreement scores at such junctions are low.

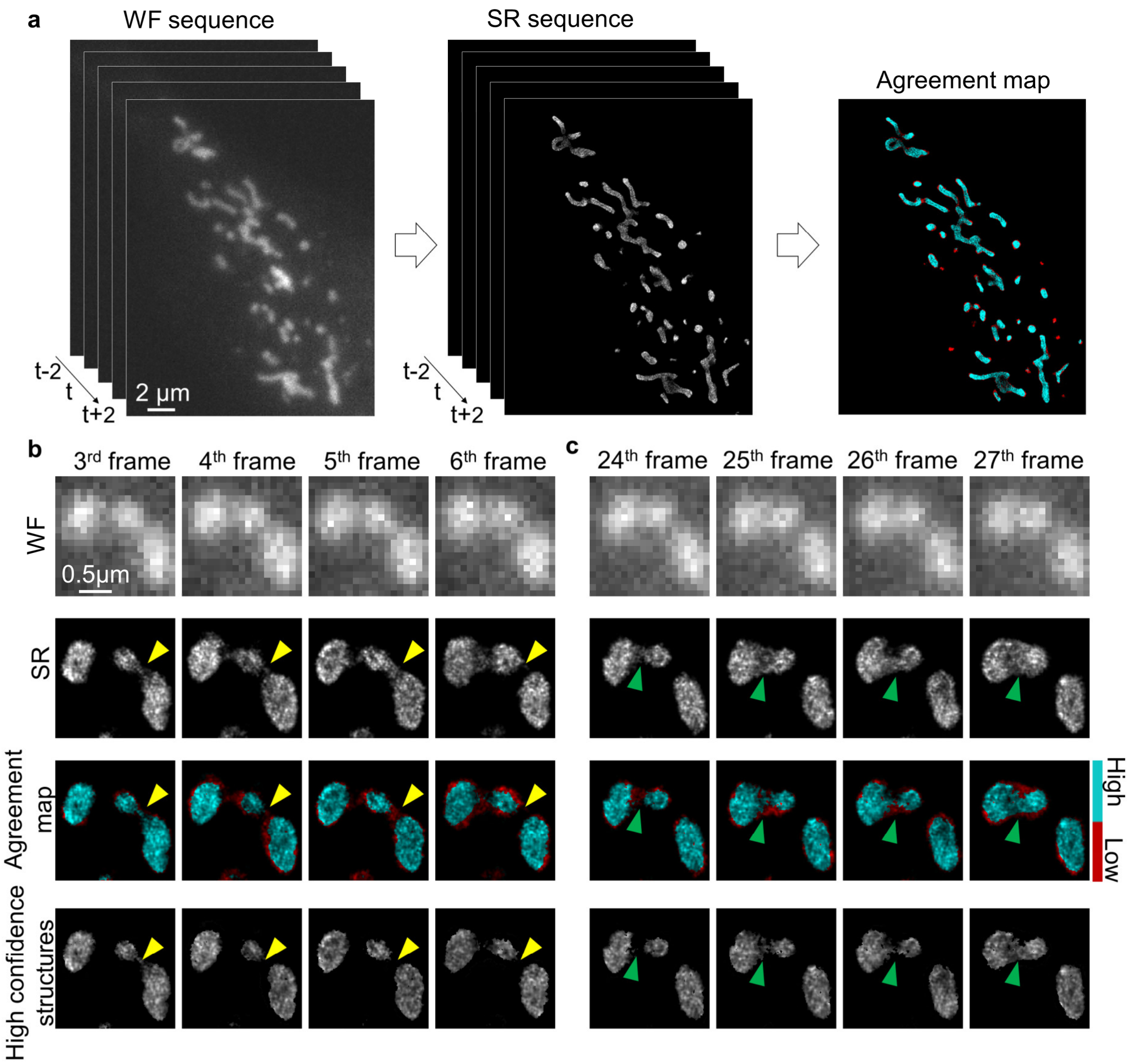

Supplementary Figure 22. Analysis of reconstruction artifacts at the mitochondrial junctions in live cells. a, The flowchart of agreement analysis in live cells. Five adjacent frames of widefield images of mitochondria in live cells are used to reconstruct the corresponding SR images. The 5 frames of SR images are then used to calculate pixelwise agreement score. The structures with an agreement score higher than 0.85 are labeled in cyan and otherwise in red to generate the agreement map. **b**, Example showing an artificial fusion event detected by the agreement map. The connections in the 4th and 5th frames of SR images (indicated by yellow arrows) have low agreement scores in the agreement map. Thus, they are regarded as artificial connections and are filtered out in high-confidence images. **c**, Example showing a fusion event with a high agreement score. The connection in the 24th frame of SR images (marked by green arrows) is detected as an artificial connection in the agreement map. While the connections in the following frames of SR images are detected as connections with a high agreement score. Thus, there is a fusion event in the high-confidence image sequence.

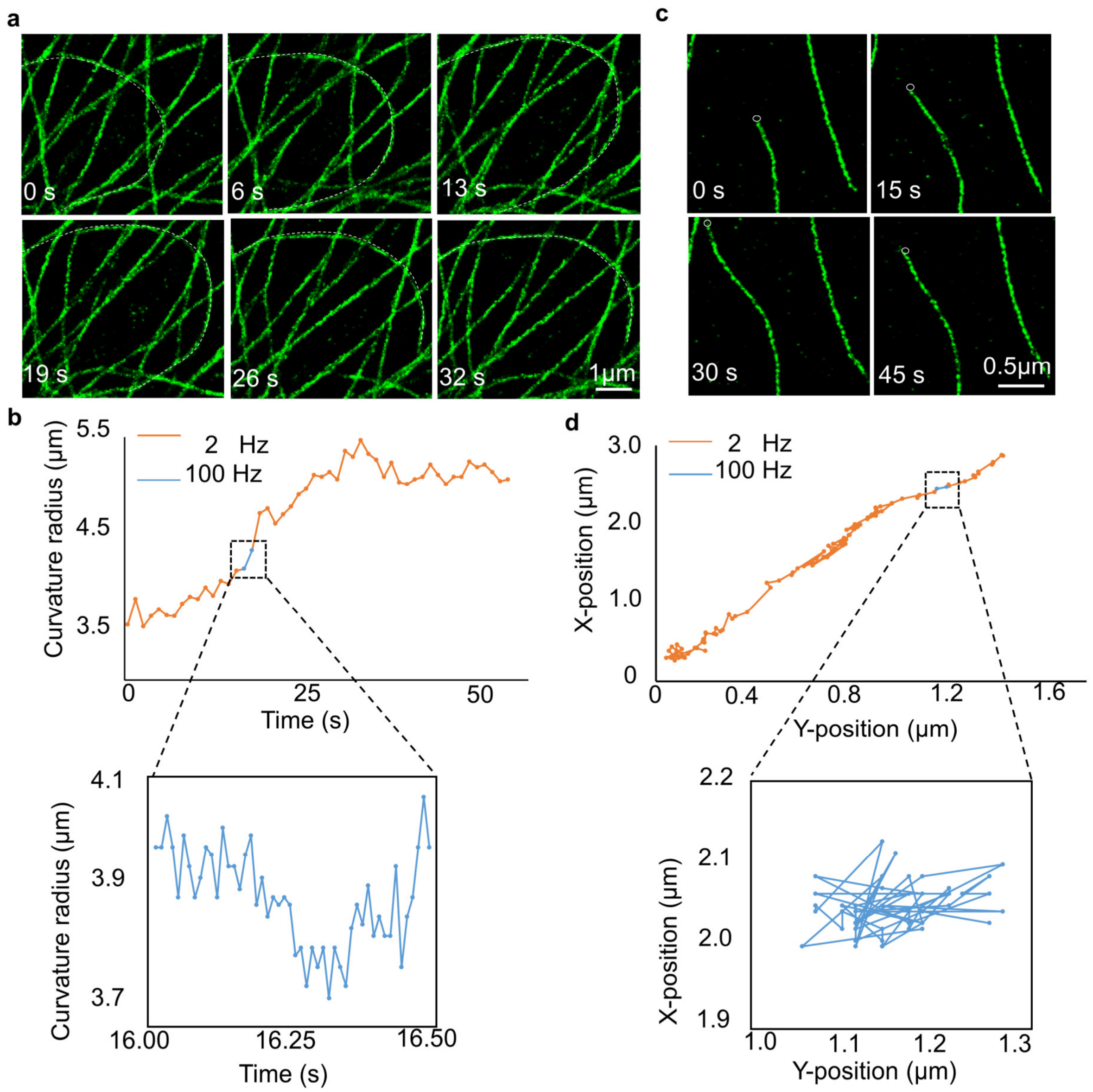

Supplementary Figure 23. Microtubule bending dynamics and microtubule tip growth and shrink instability. a, Microtubule bending dynamics. **b**, The curvature radius change of the microtubule marked by the white dashed line in **a** recorded at 2 Hz and 100 Hz. **c**, Microtubule tip growth and shrink instability. **d**, the microtubule tip displacement recorded at 2 Hz and 100 Hz.

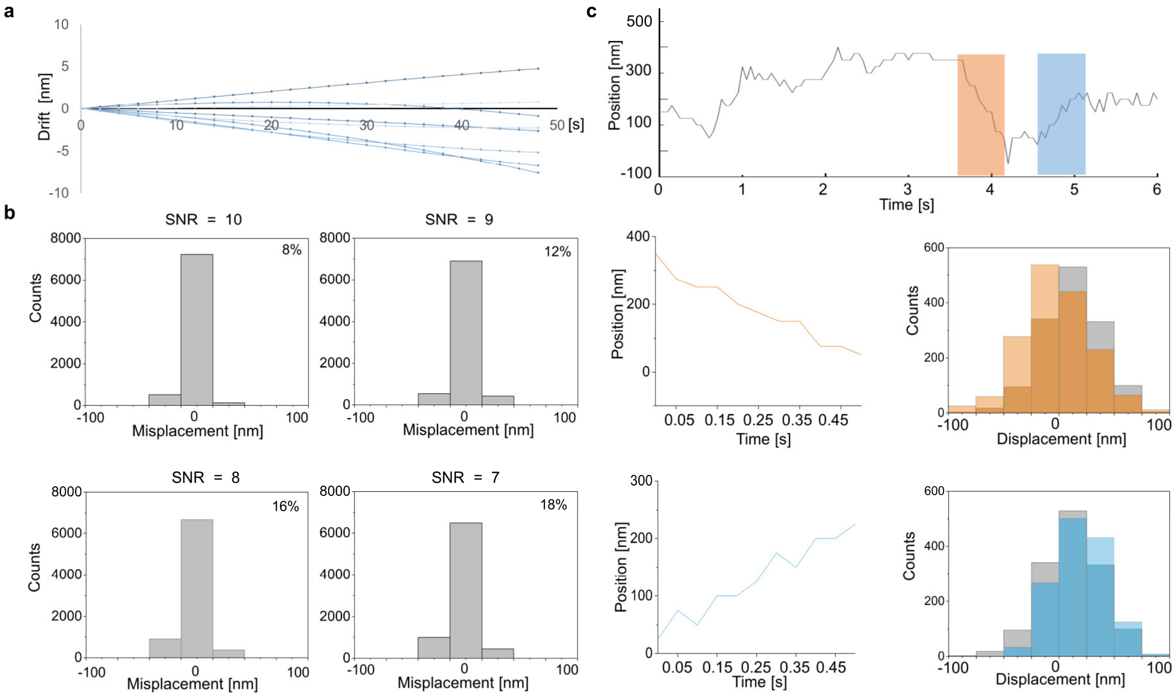

Supplementary Figure 24. System drift, noise-induced reconstruction misplacements, and microtubule transverse displacements. a, The system drift in 50 seconds tested using fluorescent beads coated on a coverslip. The experiment was repeated 8 times, showing that the drift was consistently smaller than 10 nm in 50 seconds which was the duration of our high-speed live-cell imaging experiment. **b**, The reconstruction misplacements induced by noise. Fixed microtubules were imaged at different SNRs to get WF image sequences. The image sequences are then processed by the SFSRM network to reconstruct the SR image sequences. The microtubule positions in different frames are compared with the original position in the first frame, and the differences in their positions with respect to the original are recorded as misplacements. The result shows that the misplacements are within the ± 25 nm range, and the percentage increases as the SNR decreases. For SNR of 7, the observed misplacement percentage is about 18%. **c**, The transverse displacements of the microtubule in live cells. The top plot shows the transverse position change of the microtubule within six seconds. The blue and orange region indicate the rapid movement of the microtubule towards one direction which are enlarged in the second and third row, and the distributions of the displacements of the microtubule in 50-ms interval shift towards that direction accordingly.

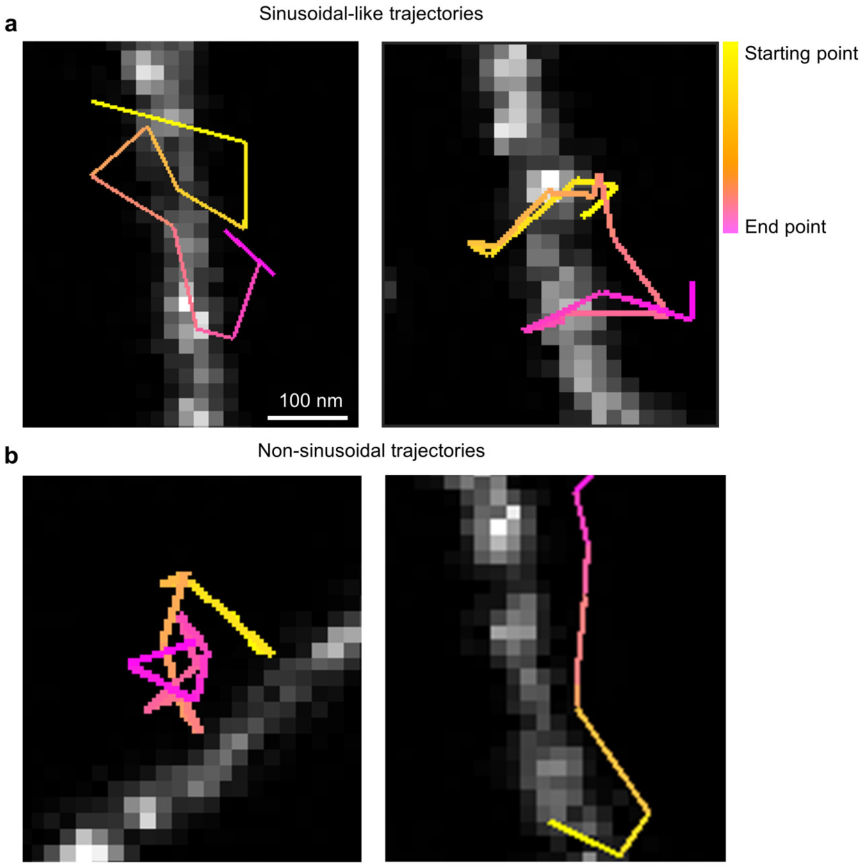

Supplementary Figure 25. Examples of sinusoidal-like and non-sinusoidal trajectories of vesicles. The trajectories of the vesicles are visualized by applying temporal color code and mapped to the corresponding images of the microtubules. **a**, Examples of the sinusoidal-like trajectories around the microtubules which cross the microtubule regularly. **b**, Examples of the non-sinusoidal trajectories which appear on one side of the microtubule or along the microtubule. We suspect the sinusoidal-like motions of the vesicles suggest plausible rotations around the microtubule rather than completely random motions.

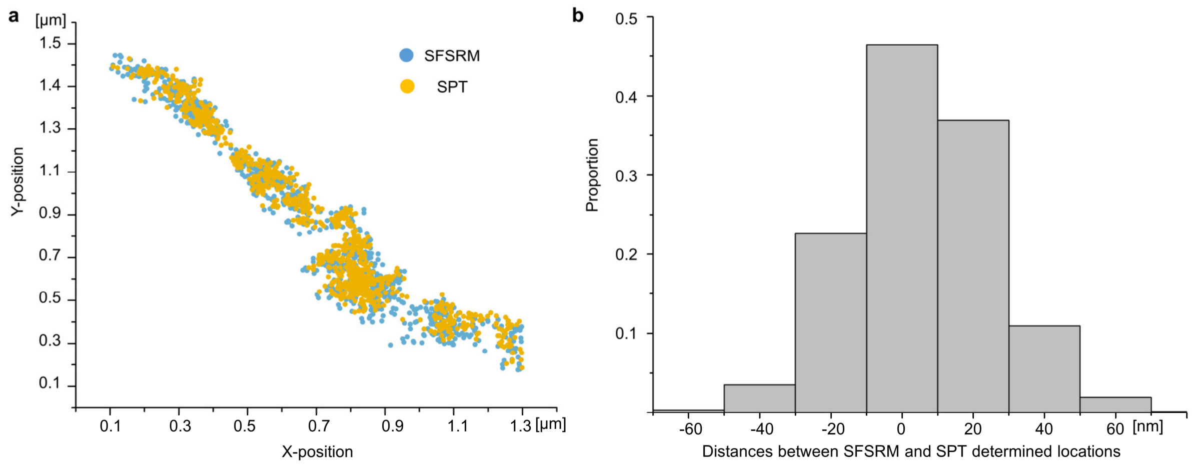

Supplementary Figure 26. Comparison of the tracking results via SFSRM and single particle tracking. a, Vesicle tracking results via SFSRM and single particle tracking (SPT). The locations of the vesicle are determined as the centroid of the vesicle from the SR image in the SFSRM method and as the center of the particles in the WF image in the SPT method. **b**, The distribution of the distances between the SFSRM and SPT determined locations.

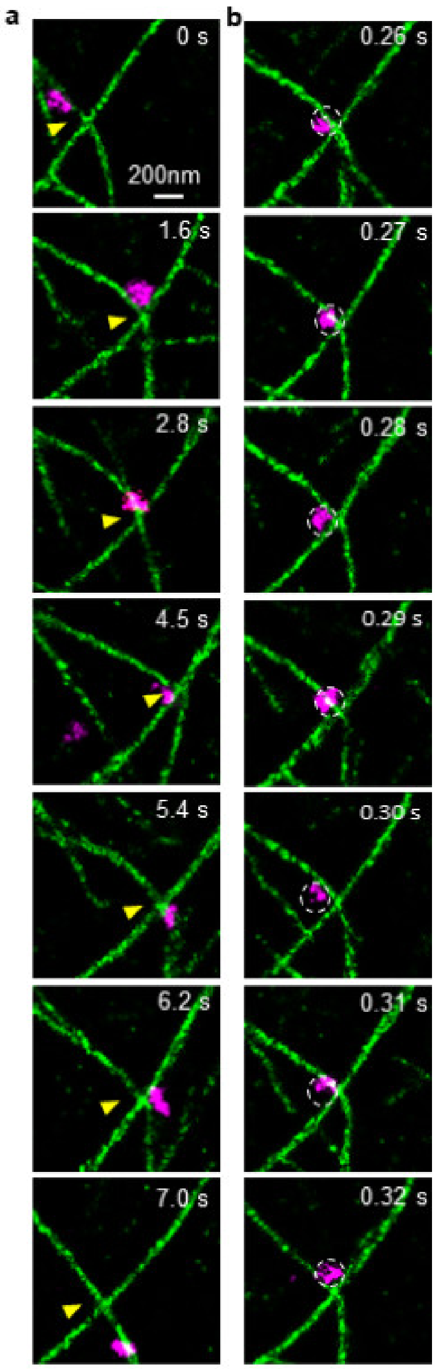

Supplementary Figure 27. Examples of microtubule morphology changes due to vesicle transport. a, The time-lapse images showing that a microtubule(labeled in green) bends to its limited position and returns to the original position. **b**, The time-lapse images showing the unstable vesicle(labeled in magenta) movement.

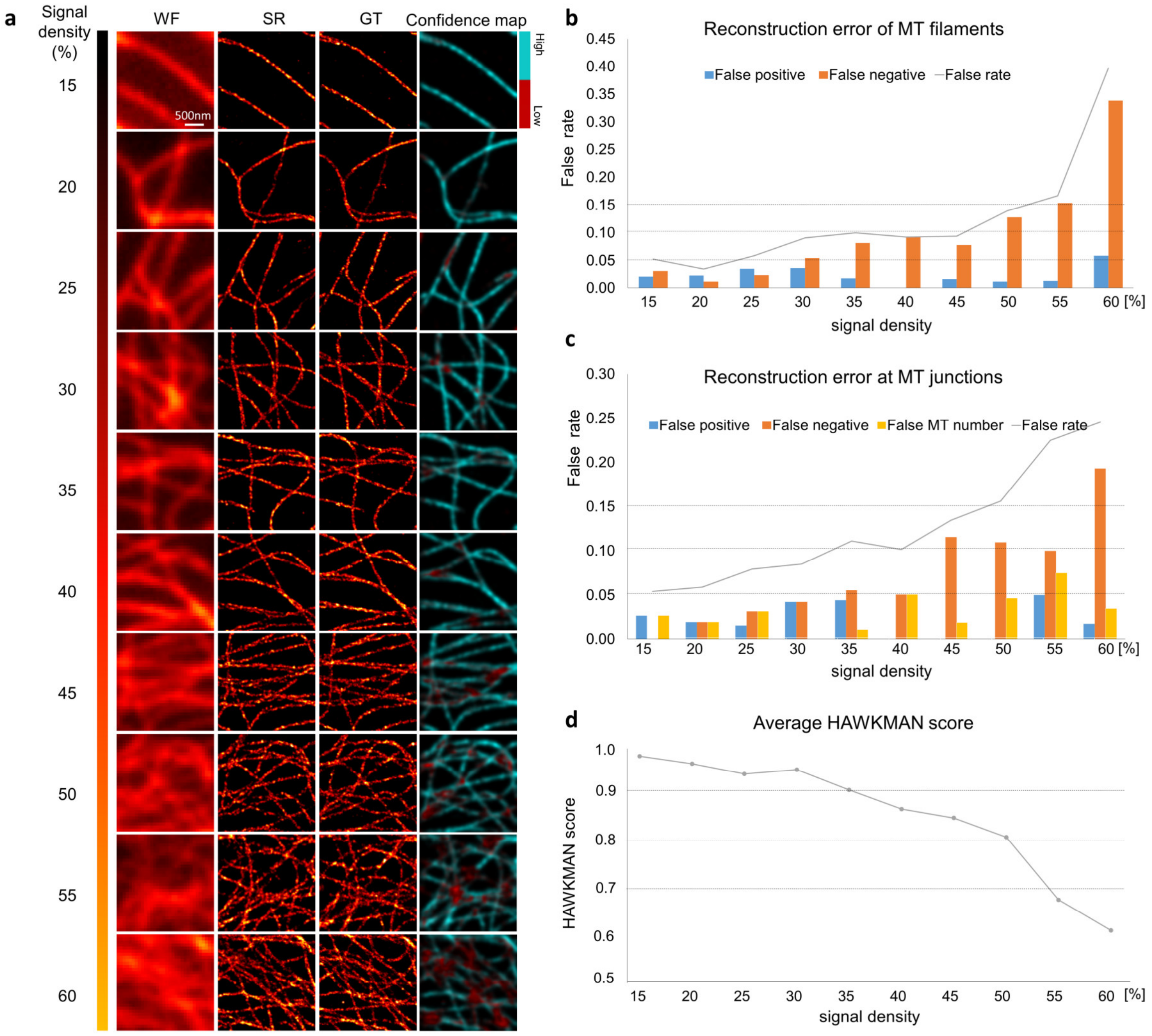

Supplementary Figure 28. Quantitative analysis of the reconstruction errors at different signal densities. **a**, The first column shows the representative WF images of different signal densities; the second column shows network reconstruction results noted as SR images; the third column shows the STORM reconstructions from 20,000 frames of single-molecule images of the same region regarded as the GT images. The signal density is calculated by first applying image binarization to the GT image to extract the structure map and then calculating the ratio of the signal-containing pixels to the total pixels of the whole image; the fourth column shows the confidence maps of the corresponding SR images. **b**, Reconstruction error of single microtubules including reconstructing an artificial microtubule or missing some parts of the microtubule, the overall false rate is plotted as a function of signal densities. When the signal density is lower than 0.5, the reconstruction false rate can be kept lower than 0.15. The false rate rapidly increases to 0.45 when the signal density is higher than 0.6. **c**, Reconstruction error of the microtubule intersections including artificially reconstructed intersection, missing intersection, or a wrong number of microtubules at the intersection. Similar to the false rate of the microtubules, the false rate of the intersections is lower than 0.15 when the signal density is lower than 0.5, and it drastically increases to 0.3 when the signal density increases to 0.6. The main reason for the rapid increase of the false rate at high signal density is that the network has a high possibility to miss some structures when the signal is too dense, the miss of the microtubule will further results in the false reconstruction of the microtubule intersections. **d**, Plot of the HAWKMAN score of the reconstruction result as a function of the signal density, which is inversely correlated to the trend of the false rate.

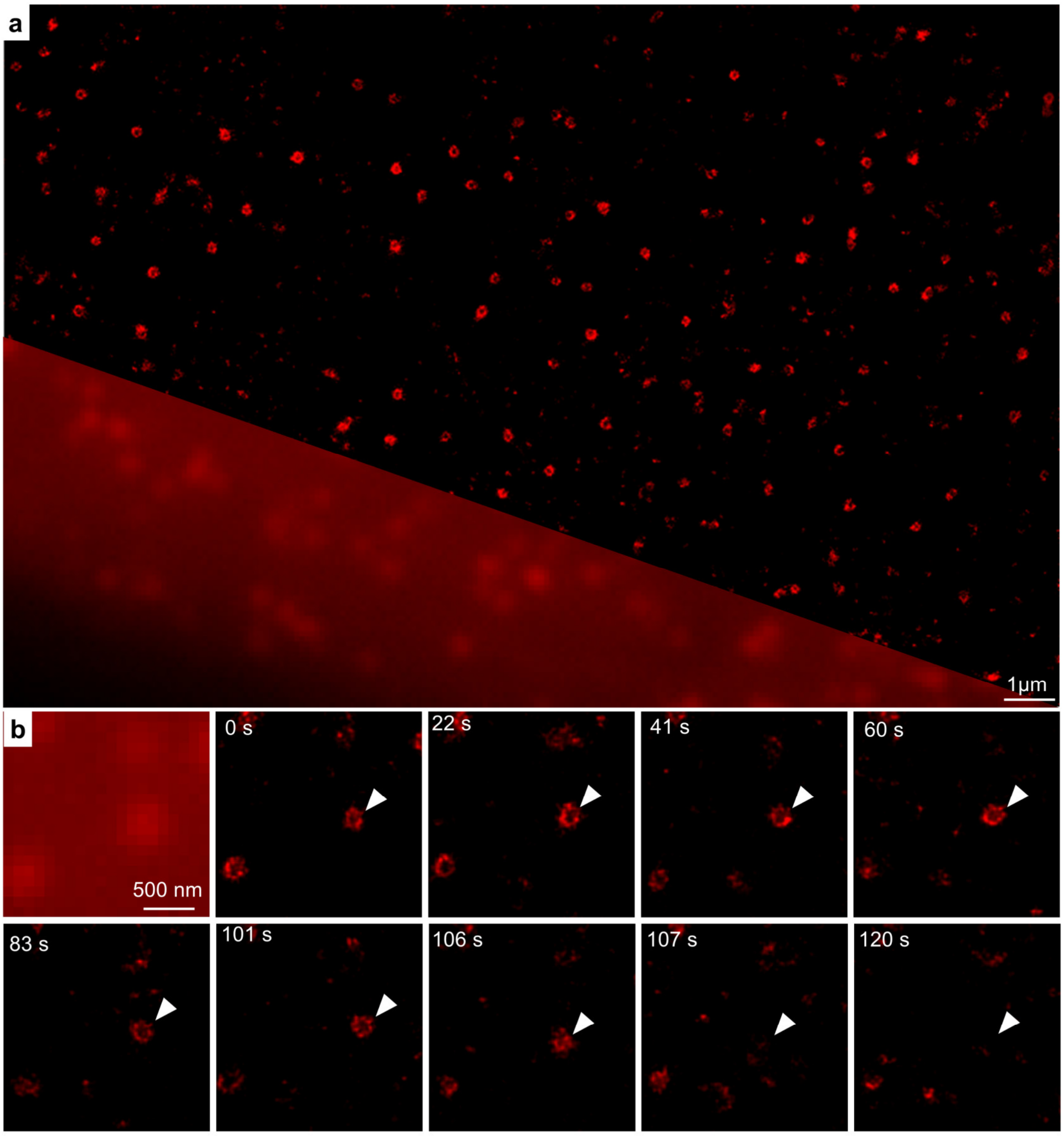

Supplementary Figure 29. SFSRM reconstruction of the clathrin-coated pits (CCPs) in live cells. a, Representative WF and SR image of CCPs in cells expressing Halo-clathrin. **b**, Time-lapse images showing the dynamics of the CCP indicated by white arrows.

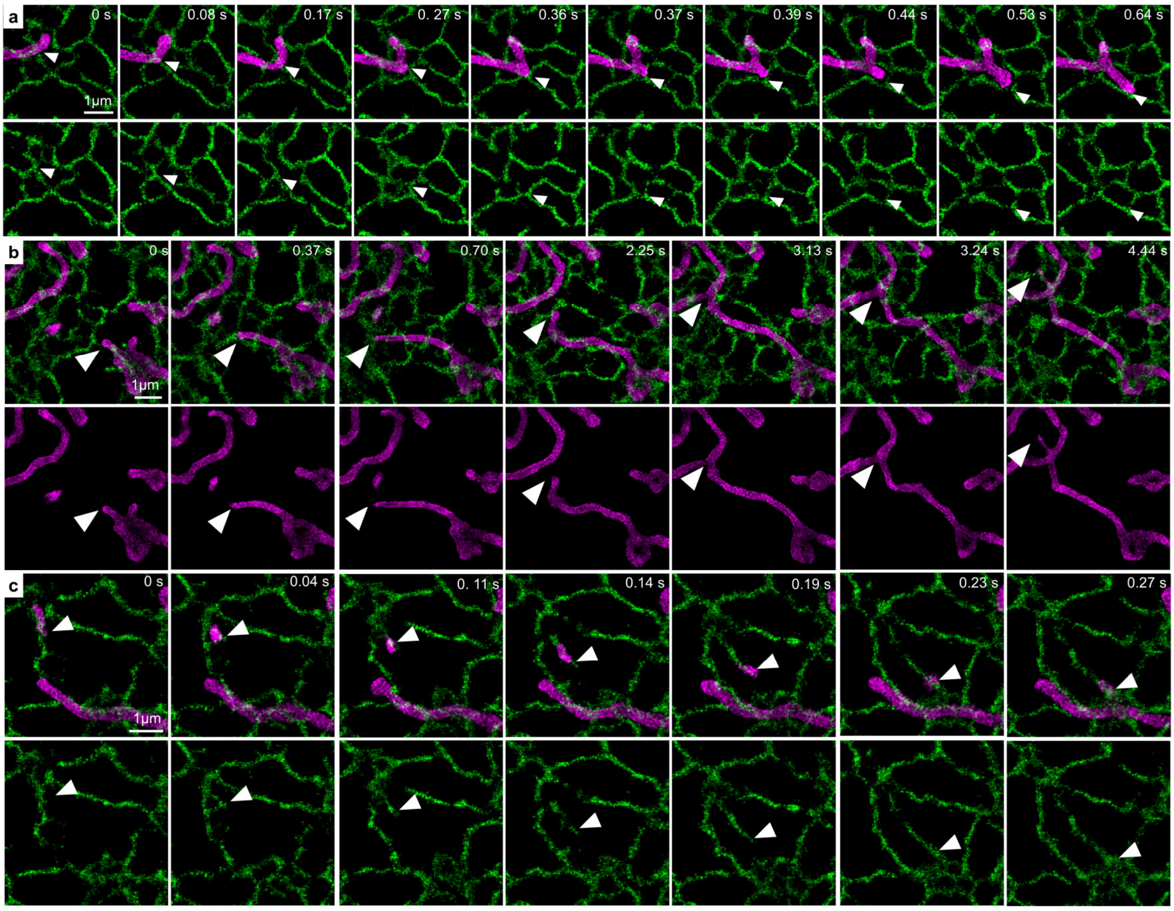

Supplementary Figure 30. SFSRM imaging of interactions between the mitochondria and endoplasmic reticulum (ER) in live cells. **a**, Examples of the mitochondrial branching along the ER tubule. The mitochondria (mito) are rendered in magenta and ER is rendered in green. White arrows indicate the ER-mito contact site. **b**, Examples of the mitochondrial growth along the ER tubules. White arrows indicate the mitochondrial tip position. **c**, Examples of ER tubule growth by hitchhiking on the moving mito. White arrows indicate the ER tip position.

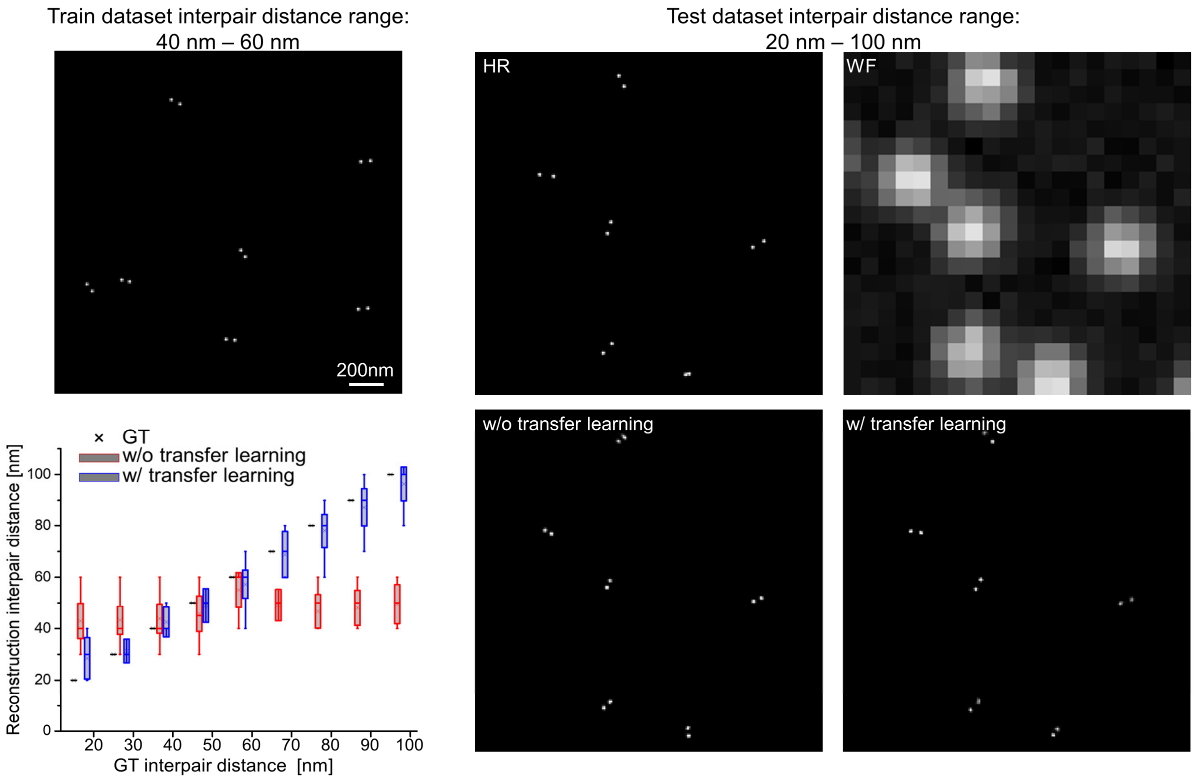

Supplementary Figure 31. Test network performance on data outside the training range using simulated dot pairs. The network is trained by dot pairs with distances between 40 nm and 60 nm. The test data are dot pairs with distances between 20 nm and 100 nm. The quantitative analysis of the reconstructed interpair distances shows that the network cannot correctly reconstruct dot pairs with distance outside the training range. Boxplots are drawn from the 25th to 75th percentile with the horizontal bar at the median and the whiskers extending to the minima and maxima. After fine-tuning the trained network with the dataset that matches the test dataset range for about 10 epochs, the network can output correct reconstruction results

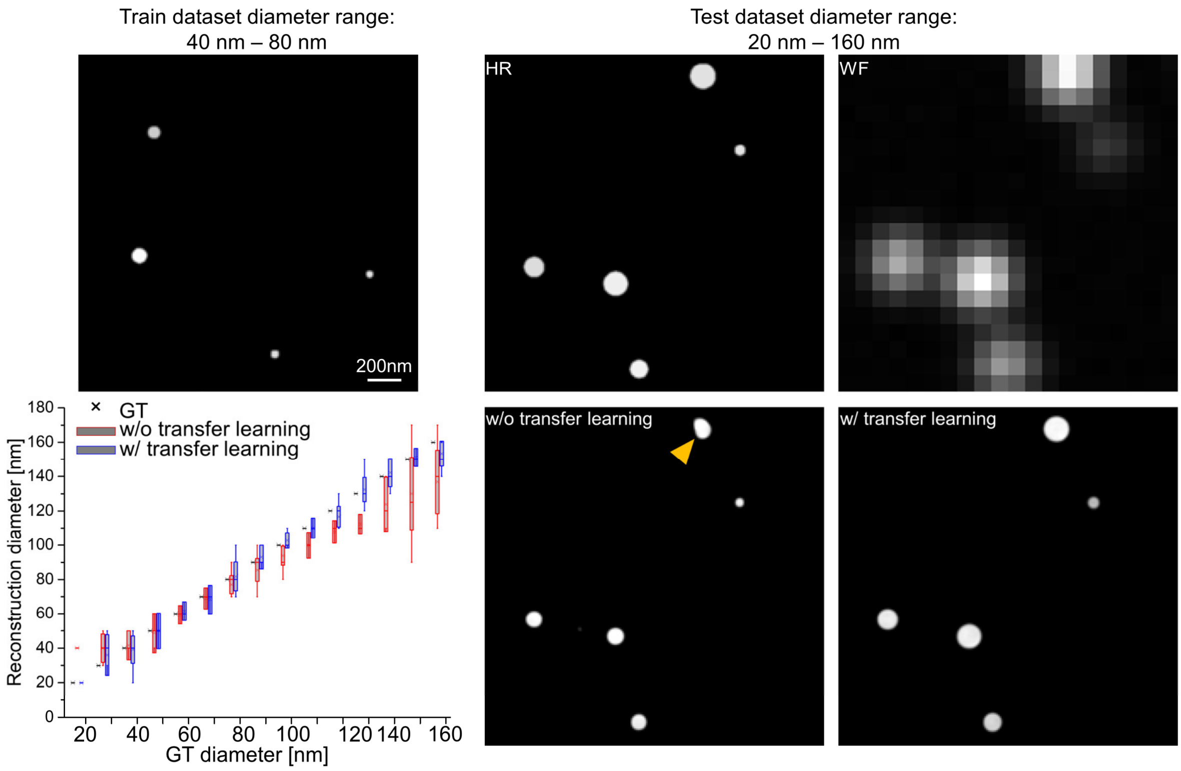

Supplementary Figure 32. Test network performance on data outside the training range using simulated clusters. The network is trained by clusters with diameters between 40 nm and 80 nm. The test data are clusters with diameters between 20 nm and 160 nm. The quantitative analysis of the reconstructed diameters shows that the network cannot recognize the data drift when the input diameter is less than 140 nm and will output biased results; while when the input diameter is larger than 140 nm, the network can recognize the data drift and output abnormal results (indicated by the orange arrow). Boxplots are drawn from the 25th to 75th percentile with the horizontal bar at the median and the whiskers extending to the minima and maxima. After fine-tuning the trained network with the dataset that matches the test dataset range for about 10 epochs, the network can reconstruct clusters with correct diameters.

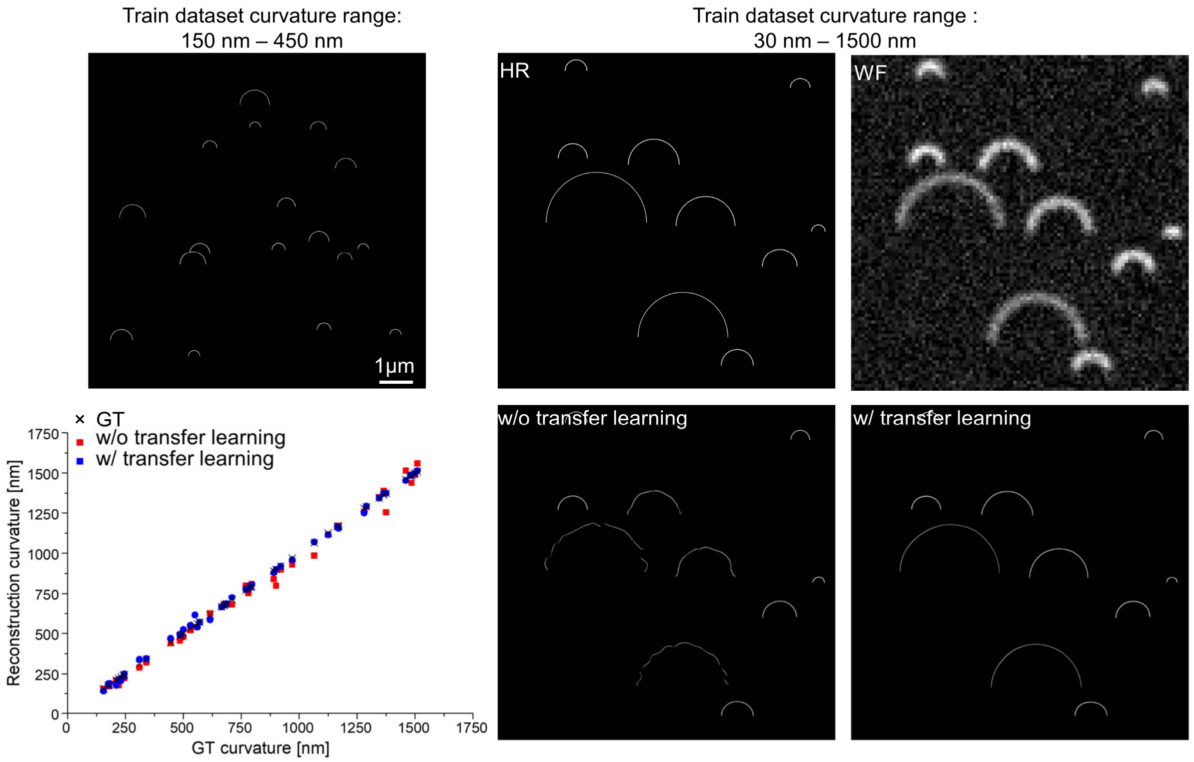

Supplementary Figure 33. Test network performance on data outside the training range using simulated curves. The network is trained by curves with curvatures between 150 nm and 450 nm. The test data are clusters with diameters between 30 nm and 1500 nm. The result shows that the network can reconstruct curves with roughly right curvature when the test curvatures are far beyond the training dataset range. However, the features within the diffraction limit are still not correctly reconstructed. After fine-tuning the trained network with the dataset that matches the test dataset range for about 10 epochs, the network can reconstruct curves with correct curvature and perfect features.

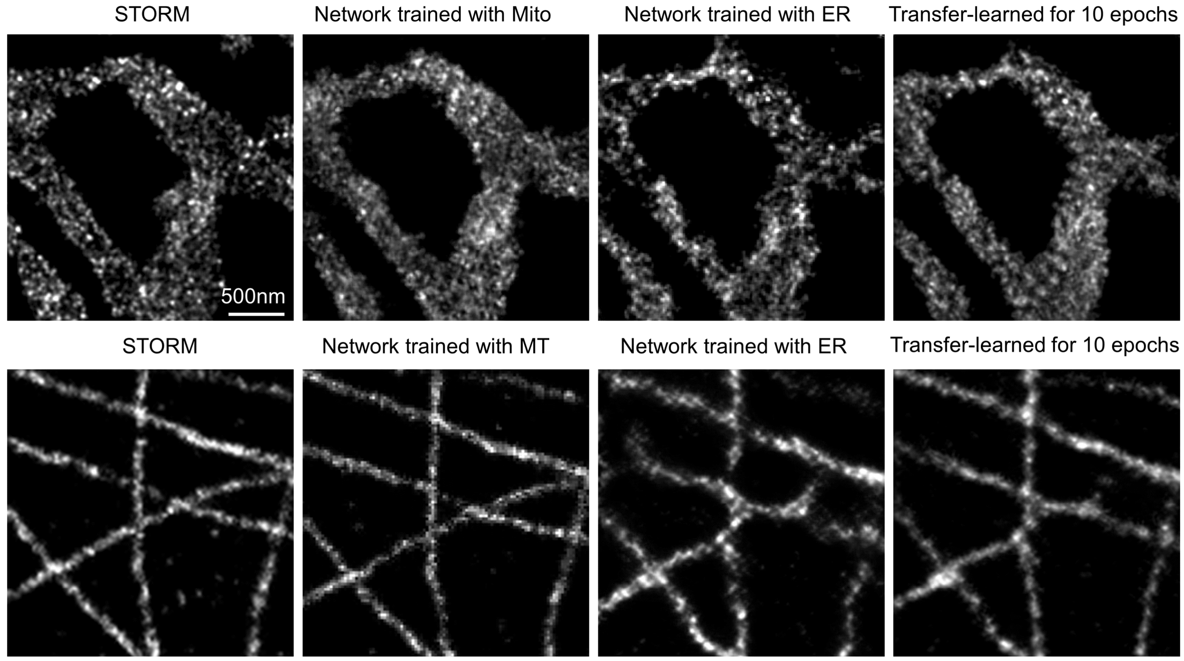

Supplementary Figure 34. Test network performance on mismatched subcellular structures. The first column shows the STORM images of the mitochondria (mito) and microtubules (MT) in fixed cells. The second column shows the reconstruction results of mito and MT using the matched networks. The third column shows the reconstruction results of mito and MT using the network trained by the endoplasmic reticulum (ER). The fourth column shows the reconstruction results of mitochondria and microtubules via networks first trained with ER data and then fine-tuned with corresponding mito/MT data for 10 epochs.

References

1. Zhao, Q., Young, I. T. & De Jong, Jan Geert Sander. Photon budget analysis for fluorescence lifetime imaging microscopy. *J. Biomed. Opt.* **16**, 086007 (2011).

2. Cubitt, A. B., Woollenweber, L. A. & Heim, R. Chapter 2: Understanding Structure—Function Relationships in the Aequorea victoria Green Fluorescent Protein. *Methods Cell Biol.* **58**, 19-30 (1998).

3. Shaner, N. C. *et al*. Improving the photostability of bright monomeric orange and red fluorescent proteins. *Nature methods* **5**, 545 (2008).

4. Wäldchen, S., Lehmann, J., Klein, T., Van De Linde, S. & Sauer, M. Light-induced cell damage in live-cell super-resolution microscopy. *Scientific reports* **5**, 1-12 (2015).

5. Marsh, R. J. *et al*. Sub-diffraction error mapping for localisation microscopy images. *Nature communications* **12**, 1-13 (2021).

6. Kendall, A. & Gal, Y. What uncertainties do we need in bayesian deep learning for computer vision? *Advances in neural information processing systems* **30** (2017).

7. Weigert, M. *et al*. Content-aware image restoration: pushing the limits of fluorescence microscopy. *Nature methods* **15**, 1090-1097 (2018).

8. Descloux, A., Grußmayer, K. S. & Radenovic, A. Parameter-free image resolution estimation based on decorrelation analysis. *Nature methods* **16**, 918-924 (2019).
