# Supplementary material for: Single-frame deep-learning super-resolution microscopy for intracellular dynamics imaging
Source: Nat Commun. 2023 May 18;14:2854. doi: 10.1038/s41467-023-38452-2 (PMC10195829; doi:10.1038/s41467-023-38452-2)
Supplement: Supplementary file 2 — Reporting Summary [file 41467_2023_38452_MOESM2_ESM.pdf]

nature portfolio

Corresponding author(s): Shengwang Du, Yusong Guo, Shuhuai Yao

Last updated by author(s): Apr 19, 2023

Reporting Summary

Nature Portfolio wishes to improve the reproducibility of the work that we publish. This form provides structure for consistency and transparency in reporting. For further information on Nature Portfolio policies, see our Editorial Policies and the Editorial Policy Checklist.

Statistics

For all statistical analyses, confirm that the following items are present in the figure legend, table legend, main text, or Methods section.

n/a	Confirmed	
☐	☒	The exact sample size (*n*) for each experimental group/condition, given as a discrete number and unit of measurement
☐	☒	A statement on whether measurements were taken from distinct samples or whether the same sample was measured repeatedly
☒	☐	The statistical test(s) used AND whether they are one- or two-sided<br>*Only common tests should be described solely by name; describe more complex techniques in the Methods section.*
☒	☐	A description of all covariates tested
☒	☐	A description of any assumptions or corrections, such as tests of normality and adjustment for multiple comparisons
☐	☒	A full description of the statistical parameters including central tendency (e.g. means) or other basic estimates (e.g. regression coefficient) AND variation (e.g. standard deviation) or associated estimates of uncertainty (e.g. confidence intervals)
☒	☐	For null hypothesis testing, the test statistic (e.g. *F*, *t*, *r*) with confidence intervals, effect sizes, degrees of freedom and *P* value noted<br>*Give P values as exact values whenever suitable.*
☒	☐	For Bayesian analysis, information on the choice of priors and Markov chain Monte Carlo settings
☒	☐	For hierarchical and complex designs, identification of the appropriate level for tests and full reporting of outcomes
☐	☒	Estimates of effect sizes (e.g. Cohen's *d*, Pearson's *r*), indicating how they were calculated

Our web collection on statistics for biologists contains articles on many of the points above.

Software and code

Policy information about availability of computer code

Data collection	The training data of fixed cells were obtained from a home-built super-resolution localization microscope based on an inverted microscope (Nikon Ti Eclipse). The live-cell data were acquired from a commercial Zeiss Elyra 7 microscope with a 60×/1.46 oil objective and a Zeiss SP8 confocal microscope with a 63×/1.4 oil objective.
Data analysis	The networks for image reconstruction were built using PyTorch 1.5. Customized Python3 scripts were built to extract edgemap images from low-resolution images. ImageJ (1.53t) and plugins were used for data visualization and quantification. Customized Matlab R2019a scripts were built for data quantification such as quantifying reconstruction error and reconstruction fidelity, and data analysis such as particle tracking and cross-correlation analysis.

For manuscripts utilizing custom algorithms or software that are central to the research but not yet described in published literature, software must be made available to editors and reviewers. We strongly encourage code deposition in a community repository (e.g. GitHub). See the Nature Portfolio guidelines for submitting code & software for further information.

Data

Policy information about availability of data

All manuscripts must include a data availability statement. This statement should provide the following information, where applicable:

- Accession codes, unique identifiers, or web links for publicly available datasets
- A description of any restrictions on data availability
- For clinical datasets or third party data, please ensure that the statement adheres to our policy

The training and test data of the neural networks is available upon the request of the editors or reviewers, and will be publicly available online after publication. Other data that support the findings of this study will be available from the corresponding authors upon reasonable request.

Human research participants

Policy information about studies involving human research participants and Sex and Gender in Research.

Reporting on sex and gender	N/A
Population characteristics	N/A
Recruitment	N/A
Ethics oversight	N/A

Note that full information on the approval of the study protocol must also be provided in the manuscript.

Field-specific reporting

Please select the one below that is the best fit for your research. If you are not sure, read the appropriate sections before making your selection.

[x] Life sciences [ ] Behavioural & social sciences [ ] Ecological, evolutionary & environmental sciences

For a reference copy of the document with all sections, see nature.com/documents/nr-reporting-summary-flat.pdf

Life sciences study design

All studies must disclose on these points even when the disclosure is negative.

Sample size	The sample size of each experiment is provided in the figure legends in the main manuscript and supplementary information files. And we performed at least 3 replications to ensure reproducibility.
Data exclusions	No data were excluded from data analysis.
Replication	The reconstruction quantification on simulation data was repeated for 100 regions. The reconstruction quantification on experimental data was repeated for 50 regions. The observed and analyzed events related to live-cell experiments were provided in the corresponding figure legends. No failed reconstruction was observed.
Randomization	The samples were randomly chosen before imaging experiments. Training and testing datasets for the network model were randomly generated. Validation data was randomly chosen from the experimental dataset.
Blinding	All the performance test of the trained networks were blindly operated on data that were not included in the network training process.

Reporting for specific materials, systems and methods

We require information from authors about some types of materials, experimental systems and methods used in many studies. Here, indicate whether each material, system or method listed is relevant to your study. If you are not sure if a list item applies to your research, read the appropriate section before selecting a response.

Materials & experimental systems

n/a	Involved in the study
☐	☒ Antibodies
☐	☒ Eukaryotic cell lines
☒	☐ Palaeontology and archaeology
☒	☐ Animals and other organisms
☒	☐ Clinical data
☒	☐ Dual use research of concern

Methods

n/a	Involved in the study
☒	☐ ChIP-seq
☒	☐ Flow cytometry
☒	☐ MRI-based neuroimaging

Antibodies

Antibodies used

Primary antibody: mouse anti-α-tubulin antibody (1:500) (Sigma, T6199) ; Anti-EGFR Antibody (R-1) (1:500) (scbt, sc-101); HA-Tag (C29F4) Rabbit mAb (1:500) (Cell Signaling, 3724);anti-clathrin heavy chain antibody(1:200) (Abcam, ab2731);anti-Nup133 antibody (1:100)(Sigma-Aldrich, HPA059767) . Secondary antibody: Alexa Fluor 647 goat anti-mouse IgG (1:500) (Invitrogen, A-21236); goat anti-rabbit Alexa Fluor 647 (1:500) (Sigma-Aldrich, SAB4600184); Alexa Fluor 488 goat anti mouse antibody (1:500) (Abcam, ab150113); Alexa Fluor 568 donkey anti-Mouse IgG (H+L) (1:500) (ThermoFisher, A10037);anti-GFP primary antibody (1:500) (Proteintech, 50430-2-AP)

Validation

(1) mouse anti-α-tubulin antibody
species reactivity: yeast, mouse, amphibian, human, rat, chicken, fungi, bovine
application: immunocytofluorescence, western blot analysis
validation: https://www.sigmaaldrich.com/US/en/product/sigma/t6199
(2) Anti-EGFR Antibody (R-1)
species reactivity: human, rat, mouse
application: immunohistochemistry, immunocytochemistry, western blot,etc
validation: https://www.labome.com/product/Santa-Cruz-Biotechnology/sc-101.html
(3) HA-Tag (C29F4) Rabbit mAb
species reactivity: All Species Expected
application: immunohistochemistry, immunocytochemistry, western blot,etc
validation: https://www.cellsignal.com/products/primary-antibodies/ha-tag-c29f4-rabbit-mab/3724
(4) anti-clathrin heavy chain antibody
species reactivity: Human, Xenopus laevis
application: western blot, immunohistochemistry, immunofluorescence
validation: https://www.abcam.com/products/primary-antibodies/clathrin-heavy-chain-antibody-x22-ab2731.html
(5) anti-Nup133 antibody
species reactivity: Human
application: immunoblotting, immunofluorescence,immunohistochemistry
validation: https://www.sigmaaldrich.com/US/en/product/sigma/hpa059767

Eukaryotic cell lines

Policy information about cell lines and Sex and Gender in Research

Cell line source(s)	The Beas2B cell line was bought from ATCC (CRL-9609).
Authentication	The Beas2B cell line used in this study was not authenticated by our lab.
Mycoplasma contamination	The cell lines have been tested negative for mycoplasma contamination.
Commonly misidentified lines (See ICLAC register)	There were no misidentified cell lines used in this study.
