# Supplementary material for: Single-frame deep-learning super-resolution microscopy for intracellular dynamics imaging
Source: Nat Commun. 2023 May 18;14:2854. doi: 10.1038/s41467-023-38452-2 (PMC10195829; doi:10.1038/s41467-023-38452-2)
Supplement: Supplementary file 3 — Description of Additional Supplementary Files [file 41467_2023_38452_MOESM3_ESM.docx]

**Supplementary Movies**

**Supplementary Movie 1**

**Description:** The real-time imaging of endoplasmic reticulum in a live cell via SFSRM. Beas2B cells were transfected with EGFP-Sec61β plasmids. A region of 25.6 ×25.6 µm^2^ was chosen to record the video at 100 Hz for 5000 time points. The illumination intensity was set as 15 W/cm^2^ during imaging.

**Supplementary Movie 2**

**Description:** The real-time imaging of mitochondria in a live cell via SFSRM. Beas2B cells were transfected with Tomm20-mCherry plasmids. A region of 25.6 ×25.6 µm^2^ was chosen to record the video at 100 Hz for 5000 time points. The illumination intensity was set as 15 W/cm^2^ during imaging.

**Supplementary Movie 3**

**Description:** The real-time imaging of microtubules in a live cell via SFSRM shows the diverse microtubule dynamics such as bend, growth or shrink, and transverse vibration. Beas2B cells were transfected with mEmerald-ensconsin plasmid. A region of 25.6 ×25.6 µm^2^ was chosen to record the video at 100 Hz for 5000 time points. The illumination intensity was set as 15 W/cm^2^ during imaging.

**Supplementary Movie 4**

**Description:** The dual-color real-time imaging of EGF protein and microtubules in live cells via SFSRM reveals the vesicle-microtubule interaction dynamics during the transport process including diffusive motions, nondirected transport, and passing through microtubule intersections. Beas2B cells were transfected with mEmerald-ensconsin plasmid and then incubated with Qdot655-labeled EGF protein to allow EGF endocytosis. For the whole-cell imaging in the first part of the video, a region of 60×50 µm^2^ was chosen to record the video at 20 Hz for 5000 time points. The illumination intensity was set as 3 W/cm^2^ during imaging. While for the high-speed dual-color imaging, a region of 25.6 ×25.6 µm^2^ was chosen to record the video at 100 Hz for 5000 time points. The corresponding illumination intensity was set as 15 W/cm^2^.

**Supplementary Movie 5**

**Description:** The demonstration of applying SFSRM to different microscopes. The video demonstrates the long-term dual-color imaging of EGFR protein and microtubules in live cells, from which we observe that microtubules form grids and participate in endosome transport. The video was recorded for 300 time points at 0.4 Hz in the region of 51.2 ×51.2 µm^2^. The illumination intensity was set as 3 W/cm^2^.

**Supplementary Movie 6**

**Description:** The dual-color real-time imaging of clathrin and EGFR protein in live cells via SFSRM. The video demonstrates clathrin-coated pits (CCPs) dynamics and the clathrin-EGFR interactions during the endosome fusion and fission process. Beas2B cells were transfected with Halo-clathrin and EGFR-EGFP plasmids and then incubated with EGF protein to allow EGF endocytosis. For the single-channel CCP imaging, the video was recorded at 1 Hz for 250 time points. The illumination intensity was set as 15 W/cm2 during imaging. While for the dual-color clathrin-EGFR imaging, the video was recorded at 0.5 Hz for 200 time points. The corresponding illumination intensity was set as 15 W/cm^2^.

**Supplementary Movie 7**

**Description:** The dual-color real-time imaging of endoplasmic reticulum (ER) and mitochondria (mito) in live cells via SFSRM reveals various ER-mito interplays including mitochondrial fusion, growth, branching, and ER tubule hitchhiking on the moving mitochondrion. Beas2B cells were transfected with EGFP-Sec61β and Tomm20-mCherry plasmids. The video was recorded at 100 Hz for 5000 time points. The corresponding illumination intensity was set as 15 W/cm^2^.

**Supplementary Software**

**Supplementary Software 1**

**Description:** Example data and demonstration of SFSRM for single-frame super resolution of microscopy images.
