# Supplementary figures and images for: Single-frame deep-learning super-resolution microscopy for intracellular dynamics imaging
Source: Nat Commun. 2023 May 18;14:2854. doi: 10.1038/s41467-023-38452-2 (PMC10195829; doi:10.1038/s41467-023-38452-2)

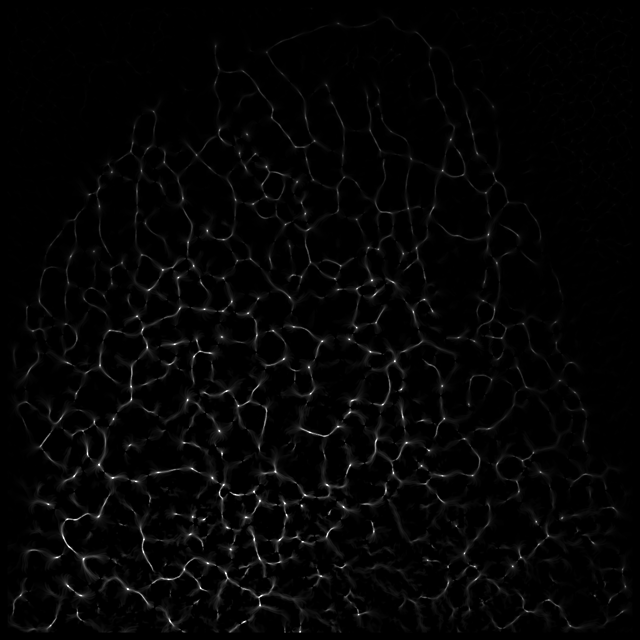

Supplement: Supplementary file 11 — Supplementary Software 1 [file 41467_2023_38452_MOESM11_ESM.zip › SFSRM-main/test_data/endoplasmic_reticulum/edgemap/ER_edgemap.tif]

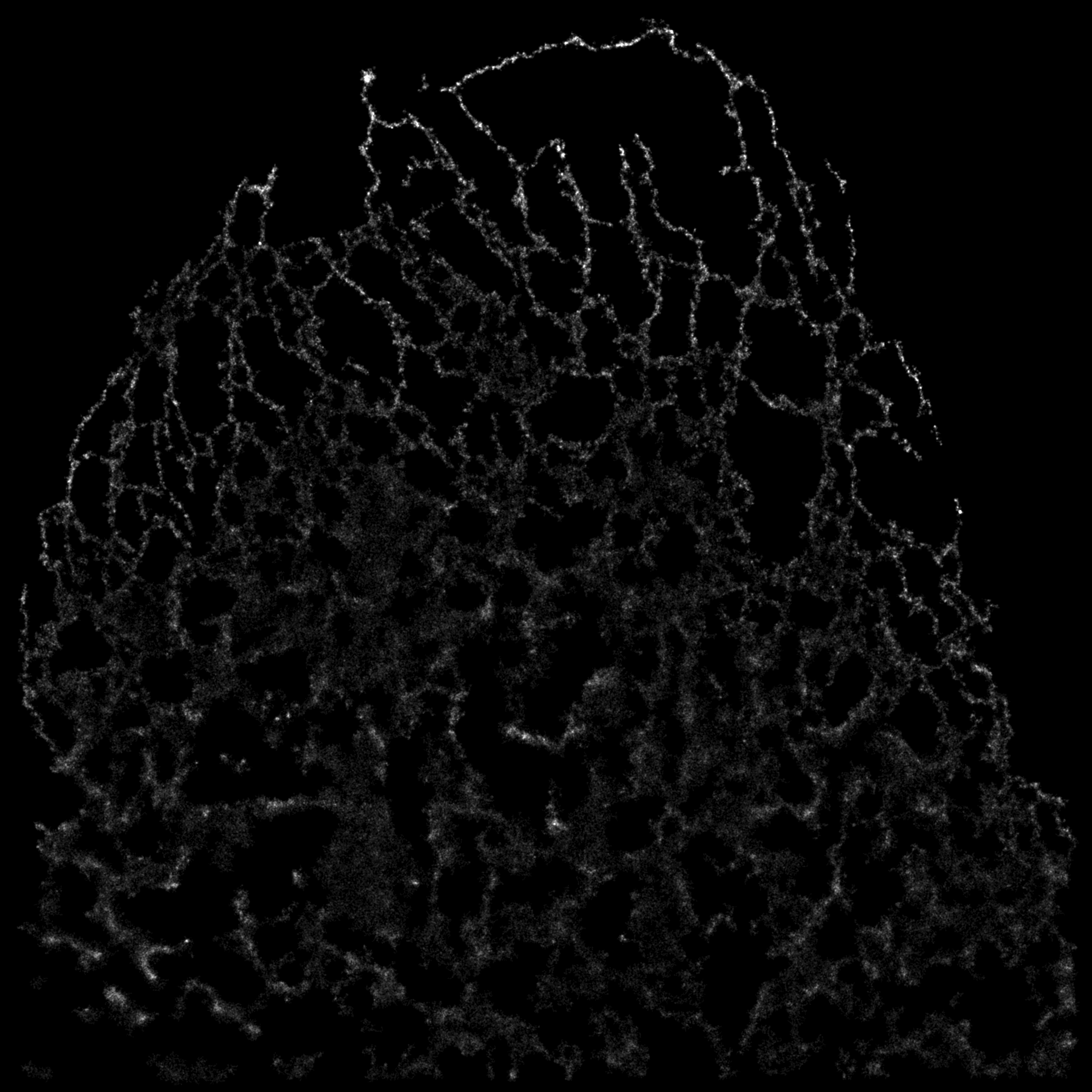

Supplement: Supplementary file 11 — Supplementary Software 1 [file 41467_2023_38452_MOESM11_ESM.zip › SFSRM-main/test_data/endoplasmic_reticulum/reference/ER_STORM.tif]

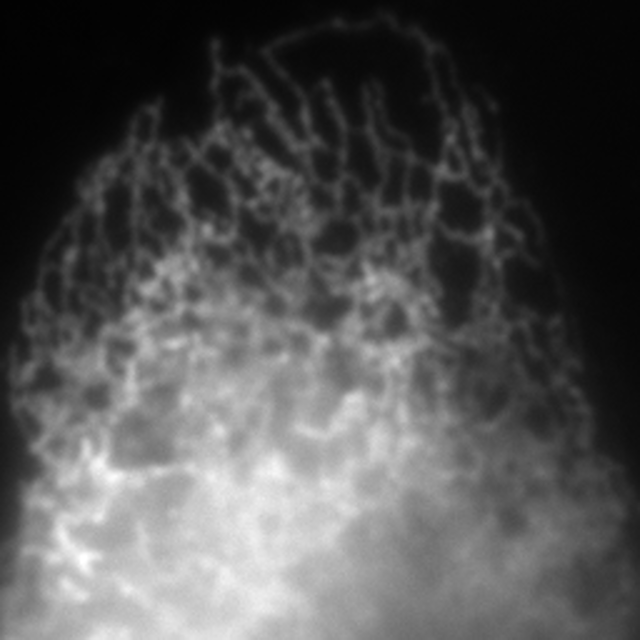

Supplement: Supplementary file 11 — Supplementary Software 1 [file 41467_2023_38452_MOESM11_ESM.zip › SFSRM-main/test_data/endoplasmic_reticulum/widefield/ER_widefield.tif]

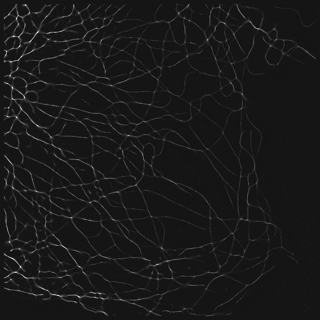

Supplement: Supplementary file 11 — Supplementary Software 1 [file 41467_2023_38452_MOESM11_ESM.zip › SFSRM-main/test_data/microtubule/edgemap/microtubule_edgemap.tif]

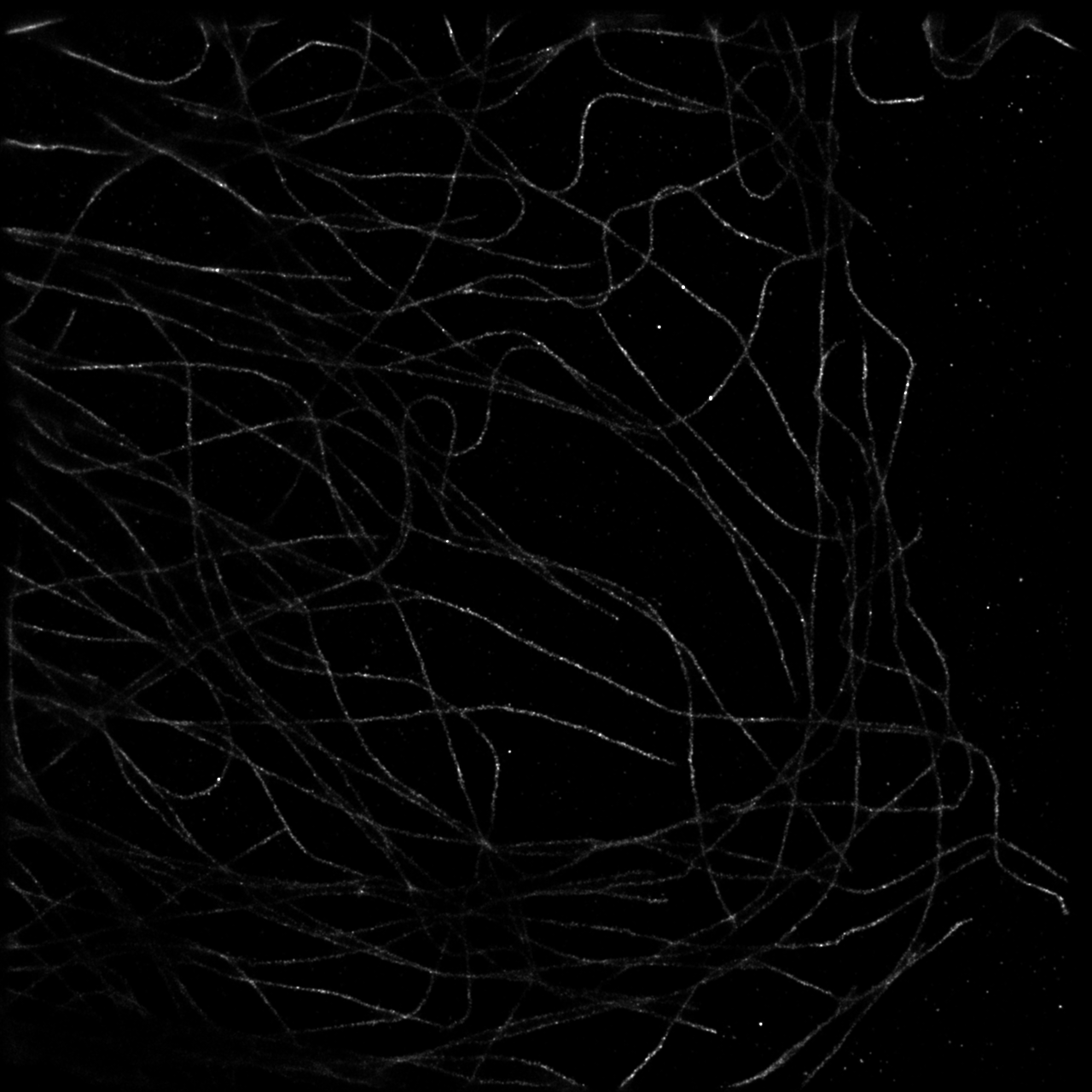

Supplement: Supplementary file 11 — Supplementary Software 1 [file 41467_2023_38452_MOESM11_ESM.zip › SFSRM-main/test_data/microtubule/reference/microtubule_STORM.tif]

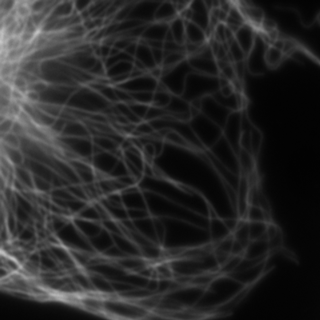

Supplement: Supplementary file 11 — Supplementary Software 1 [file 41467_2023_38452_MOESM11_ESM.zip › SFSRM-main/test_data/microtubule/widefield/microtubule_widefield.tif]

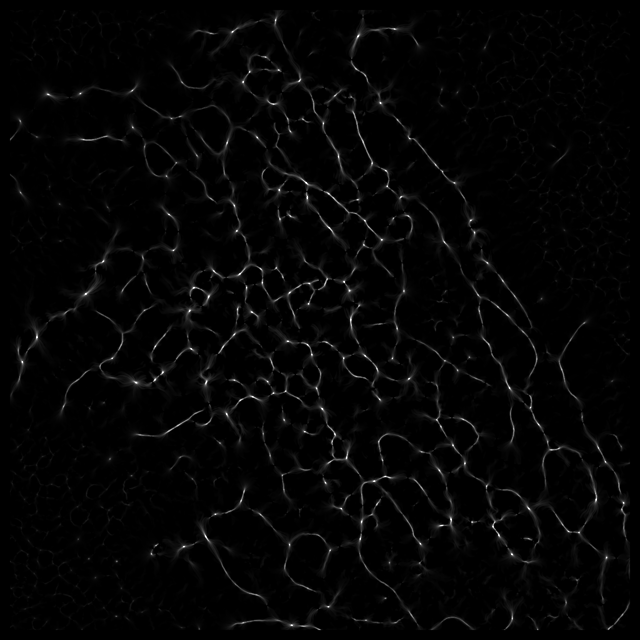

Supplement: Supplementary file 11 — Supplementary Software 1 [file 41467_2023_38452_MOESM11_ESM.zip › SFSRM-main/test_data/mitochondria/edgemap/mitochondria_endgemap.tif]

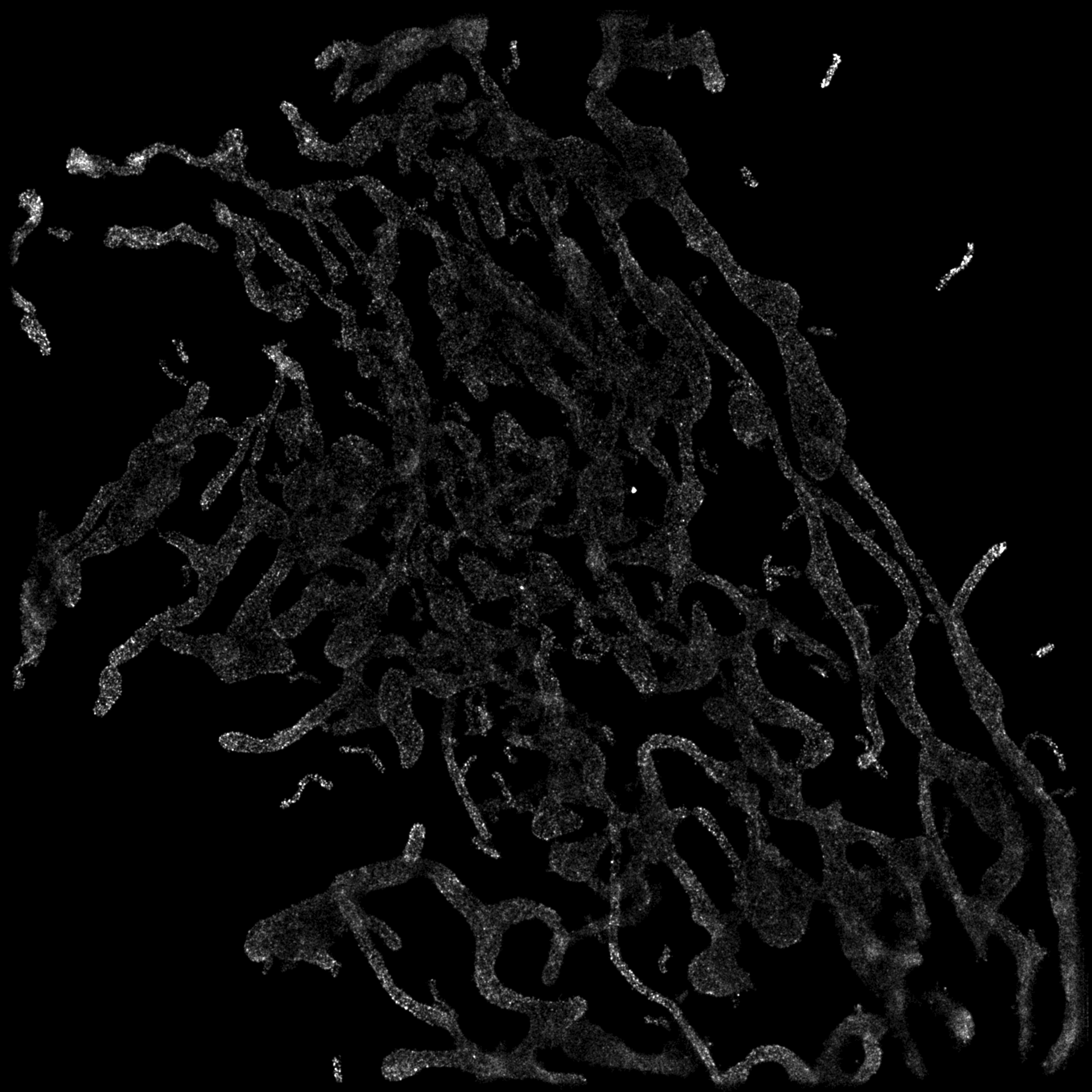

Supplement: Supplementary file 11 — Supplementary Software 1 [file 41467_2023_38452_MOESM11_ESM.zip › SFSRM-main/test_data/mitochondria/reference/mitochondria_STORM.tif]

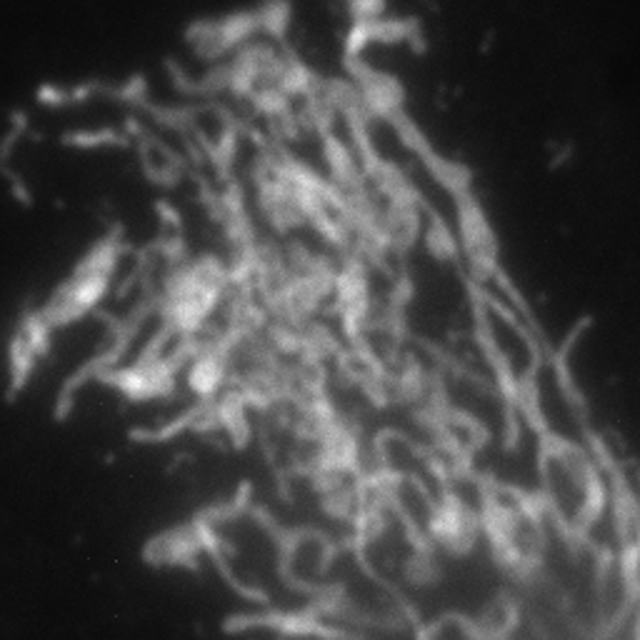

Supplement: Supplementary file 11 — Supplementary Software 1 [file 41467_2023_38452_MOESM11_ESM.zip › SFSRM-main/test_data/mitochondria/widefield/mitochondria_widefield.tif]
